# Mapping overlapping functional elements embedded within the protein-coding regions of RNA viruses

**DOI:** 10.1093/nar/gku981

**Published:** 2014-10-17

**Authors:** Andrew E. Firth

**Affiliations:** Division of Virology, Department of Pathology, University of Cambridge, Cambridge CB2 1QP, UK

## Abstract

Identification of the full complement of genes and other functional elements in any virus is crucial to fully understand its molecular biology and guide the development of effective control strategies. RNA viruses have compact multifunctional genomes that frequently contain overlapping genes and non-coding functional elements embedded within protein-coding sequences. Overlapping features often escape detection because it can be difficult to disentangle the multiple roles of the constituent nucleotides via mutational analyses, while high-throughput experimental techniques are often unable to distinguish functional elements from incidental features. However, RNA viruses evolve very rapidly so that, even within a single species, substitutions rapidly accumulate at neutral or near-neutral sites providing great potential for comparative genomics to distinguish the signature of purifying selection. Computationally identified features can then be efficiently targeted for experimental analysis. Here we analyze alignments of protein-coding virus sequences to identify regions where there is a statistically significant reduction in the degree of variability at synonymous sites, a characteristic signature of overlapping functional elements. Having previously tested this technique by experimental verification of discoveries in selected viruses, we now analyze sequence alignments for ∼700 RNA virus species to identify hundreds of such regions, many of which have not been previously described.

## INTRODUCTION

With the notable exception of smallpox virus, the majority of viruses with the potential to cause acute fatal disease in healthy adult humans are RNA viruses. Such viruses include influenza A virus (IAV), Ebola virus, rabies virus, SARS virus, MERS virus, Japanese encephalitis virus, yellow fever virus, dengue virus, eastern equine encephalitis virus and Lassa virus. Many other human pathogenic viruses are RNA viruses, including poliovirus, hepatitis A virus, hepatitis C virus, hepatitis E virus (HEV), rubella virus, chikungunya virus, Norwalk virus, mumps virus and measles virus. RNA viruses also include important pathogens of livestock, such as bluetongue virus, foot and mouth disease virus, porcine reproductive and respiratory syndrome virus (PRRSV) and Schmallenberg virus. Further, the majority of plant viruses are RNA viruses. The combined impact of RNA viruses—economically and in terms of human suffering—is immense.

RNA viruses have very compact genomes that typically only encode around 10 proteins. Many of the most important RNA viruses were amongst the first genomes to be sequenced, some 30 years ago. Surprisingly, however, ‘hidden’ protein-coding genes are still being discovered even in the most well-studied and economically important RNA viruses including the potyviruses, alphaviruses, flaviviruses, arteriviruses and IAV (1–6). Such genes tend to be very short, often overlap other genes (in an alternative reading frame) and are often expressed via non-canonical translational mechanisms. These features make such genes difficult to identify using conventional bioinformatic or experimental approaches. Current knowledge of non-coding functional elements in RNA virus genomes (e.g. essential replicational, translational and packaging signals) is also far from complete. While such elements have been reasonably well mapped in the untranslated regions (UTRs) of genomes of the most important RNA viruses, RNA virus genomes frequently contain additional non-coding functional elements embedded within the protein-coding regions.

Overlapping features are difficult to identify using experimental approaches. Systematic synonymous-site mutational analyses (7,8) are resource-intensive and can miss functional elements that are only required *in vivo*. High-throughput RNA-structure probing techniques such as SHAPE (selective 2′-hydroxyl acylation analyzed by primer extension) (9–11) and Structure-seq (*in vivo* dimethyl sulphate methylation of unpaired adenine and cytosine residues) (12,13) are often unable to distinguish functionally important RNA elements from incidental features, unless combined with comparative information (e.g. parallel analysis of divergent virus strains). High-throughput translational profiling techniques such as ribosome profiling (14,15) and high-resolution mass spectrometry (16,17)—while extremely powerful—again cannot always distinguish between functional products, regulatory translation (e.g. where translation of small upstream open reading frames, or uORFs, simply modulates translation of downstream protein-coding ORFs) and translational noise. Meanwhile, poorly translated but nonetheless functionally relevant products may be overlooked. Further, high-throughput experimental techniques have only been applied to a few select species and it is impractical to examine all of the hundreds of medically, veterinarily and agriculturally relevant virus species in this way both due to cost and the different complexities of each virus system.

The advent of cheap and rapid sequencing technologies has led to great potential for comparative genomic analysis. This potential has been realized most thoroughly for the human genome. In contrast, far less work has been done on applying comparative genomic techniques to virus genomes (18). This is surprising because many viruses—particularly RNA viruses—are uniquely amenable to comparative genomic analysis. RNA viruses have very small genomes (2–32 kb), and many are of medical or economic importance, both factors which have led to the accumulation of large numbers of sequenced isolates for many species. Furthermore, RNA viruses evolve extremely rapidly so that, even within a single species, different strains often diverge by 10–30% at the nucleotide level. By studying the patterns of substitutions in large numbers of sequences from a single virus species, or groups of related virus species, it is often possible to predict novel functional elements and gain considerable insight into their function. Where resources are limited, comparative computational analyses can be used to efficiently target experimental analysis. In contrast with many high-throughput experimental approaches, comparative genomics allows the direct detection of purifying selection which, to a very large extent, is synonymous with functional importance (19).

One particularly powerful approach is to analyze the rate of nucleotide substitutions at synonymous sites in alignments of related virus protein-coding sequences (20–22). A statistically significant reduction in variability at synonymous sites is indicative of an overlapping functional element such as an overlapping gene or functional RNA structure. Previously, we developed a novel algorithm, herein named synplot2, for this analysis (23,24). Unlike most earlier work, synplot2 simultaneously takes into account phylogeny, calculates statistical significance and does not require a training set. Previously, we have tested this technique by experimentally verifying a number of new features discovered using synplot2. These include the +1 frameshift PA-X gene in IAV (6), the -2 frameshift nsp2TF gene in arteriviruses (5), the -1 frameshift NS1’ gene in *Japanese encephalitis virus* and related flaviviruses (3,23), the -1 frameshift 2B* gene in *Encephalomyocarditis virus* (25), an extended stem-loop structure that stimulates programmed stop codon readthrough in alphaviruses (24), the 5a gene in arteriviruses (4), the non-AUG initiated Px gene in sobemoviruses (26), an unexpected subgenomic RNA (sgRNA) for capsid protein expression in *Solenopsis invicta virus 3* (27) and an essential RNA element in HEV (28).

Following this validation, we now apply the method to all RNA virus species represented in the NCBI RefSeq database that have sufficiently many sequenced isolates to generate statistically useful sequence alignments. To facilitate the analysis, we first reviewed and improved the coding sequence annotation of NCBI RNA virus RefSeqs, making >1000 revisions. We identify a number of potential new overlapping genes and hundreds of other regions with statistically significantly reduced variability at synonymous sites. For many virus species, the number and diversity of sequenced isolates is sufficient for our analysis to produce high-resolution maps giving the locations of functional overlapping elements throughout the coding regions, with implications for fundamental molecular virology, attenuated virus vaccine design by codon-deoptimization and development of virus-based gene expression vectors. The database and software are available on-line at http://www.firthlab.path.cam.ac.uk/virad.html and as Supplementary Data.

## MATERIALS AND METHODS

### Synonymous site conservation analysis

The algorithm behind synplot2 has been described previously only in brief (24). Thus a more detailed description is given here, and a user-friendly package including the source code is included as Supplementary File S1. We wished to develop a method for analyzing variability at synonymous sites that both took into account the underlying phylogeny of a sequence alignment and produced an estimate of the statistical significance of deviations from the mean. Our goal was to develop a simple and fast procedure with minimal parameters (for ease of interpretation). The goal was not to parametrize or study sequence evolution *per se*, but instead to develop a tool for identifying overlapping functional elements, allowing targeted bioinformatic (e.g. RNA structure prediction) and experimental (e.g. knockout mutant phenotype) follow-up analyses.

For a given pair of sequences within a codon-based multiple sequence alignment of a protein-coding region, a codon position was defined as a synonymous site if the same amino acid was encoded in both sequences. A substitution null model was defined such that the relative probability of each possible synonymous codon substitution (including substitution with itself) at such sites may be calculated by assuming that, for synonymous substitutions, the component nucleotides evolve neutrally. Neutral evolution was modelled using a Kimura nucleotide substitution matrix with *κ* = 3 (29). These stipulations account for the differing probabilities of transitions (purine to purine or pyrimidine to pyrimidine) and transversions (purine to pyrimidine or vice versa), and the fact that synonymous substitutions involving just a single nucleotide change (e.g. CUU to CUG leucine) should be more probable than synonymous substitutions involving additional changes (e.g. CUU to UUG leucine). Note that sites that are non-synonymous in the pairwise sequence comparison are not used at any point in the procedure.

For each sequence pair, the divergence parameter *t* was set so that the total expected number of nucleotide substitutions at synonymous sites under the null model was equal to the total observed number. Next, the difference between the expected number (exp) of nucleotide substitutions and the observed number (obs) was calculated at each synonymous site in the pairwise comparison. (The expected number of substitutions at a given synonymous site is calculated using *κ* and *t* and is normally non-integer, while the observed number is 0, 1, 2 or 3.) The expected variance at each site was calculated from the expected probabilities of each possible synonymous codon substitution assuming a multinomial distribution.

Statistics (obs minus exp, and variance) were then summed, at each alignment codon position, over a phylogenetic tree using a fast heuristic method described in (30). First a phylogenetic tree for the alignment was constructed using standard methods. Then the (unrooted) tree was used to select a list of sequence pairs tracing round the outside of an arbitrary two-dimensional representation of the tree (*N* pairs for *N* sequences), and synplot2 statistics were calculated and summed just for this set of sequence pairs. Conceptually, such a set of pairwise comparisons covers every branch of the phylogenetic tree exactly twice, so undue weight is not given to some branches over others as would occur for all-against-all pairwise comparisons. It should be noted that the set of sequence pairs used is not unique as there are many possible two-dimensional representations of a large unrooted tree. After summing at each codon position over the phylogenetic tree and dividing by two (to account for covering each branch twice), the statistics were averaged over a sliding window. An approximate *P*-value (probability that any reduction in synonymous-site variability in the window would be as great as observed if the null model were true) was also calculated, under the assumption of a normal distribution as an approximation to the sum of many independent multinomial distributions.

In principle, codon usage bias may be easily incorporated within this framework; however we chose not to, partly, because the results of applying synplot2 to viral genomes (see Results) made it clear that it was not required, and partly because it would require 60 extra parameters that would be impossible to estimate accurately given the limited genome size of RNA viruses on the one hand, and differences between virus and host codon usage on the other.

Note that in our null model the divergence parameters are determined from the full coding region of the sequence alignment, including regions containing overlapping features. If the alignment contains extensive overlapping features then the neutral divergence rates will be underestimated. Thus the software, as provided, cannot be used to determine theoretical sequence evolution parameters. In principle, the software could easily be modified to determine neutral evolution rates on regions ‘known’ not to contain overlapping features, and then this model could be applied to the full coding region. However, we developed synplot2 as a practical tool to identify overlapping features rather than a theoretical tool to calculate synonymous evolutionary rates. In our experience, even if a high proportion (e.g. 70%) of the sequence alignment comprises overlapping features, synplot2 will still enable detection of such features provided the remainder of the alignment is evolving neutrally at synonymous sites.

### Re-annotation of NCBI virus RefSeqs

In order to apply synplot2 to a large number of RNA virus species, we first had to generate suitable multiple sequence alignments. As a starting point we used all RNA virus RefSeqs (reference sequences) in NCBI GenBank. To analyze synonymous site variation, we first had to define the coding regions of each RefSeq. Current GenBank annotation for RNA virus coding sequences is imperfect, partly due to difficulties with annotating unusual translational phenomena that are so abundantly used by RNA viruses (31), so our first task was to re-annotate the RefSeq translatome.

Sites of programmed transcriptional slippage, ribosomal frameshifting and stop codon readthrough (which are often annotated incorrectly or even omitted) were corrected according to the literature on homologous cases (24,31,32). While -1 frameshift sites are relatively straightforward to annotate due to well-defined associated sequence motifs (31), sites of +1 frameshifting are less well studied and often involve simpler and therefore more-difficult-to-distinguish motifs. Comparative genomic analysis can aid identification of the correct frameshift site as frameshift sites are generally highly conserved between related species. Based on this and the literature, we annotated sites of known or predicted influenza-PAX-like +1 frameshifting in IAV, fijiviruses, *Chronic bee paralysis virus* and related viruses and amalgamaviruses according to (33). Programmed +1 frameshifting in closterovirids was assumed to involve P-site slippage on the GUU_U of a GUU_stop_C sequence that is highly conserved at the ORF1 stop codon in most closterovirids (31,34). In ampeloviruses (family *Closteroviridae*) that lack this motif, +1 P-site slippage was assumed to occur on the UUU_C of a slightly upstream UUU_CGA sequence (PAX-like) that is conserved in many other ampelovirus sequences. Finally in *Citrus tristeza closterovirus*, where ORF1 is extended some 25 codons relative to other closteroviruses, +1 frameshifting was assumed to occur on the GUU_C of a GUU_CGG sequence that aligns to the GUU_stop in other closteroviruses (35). In *Leishmania RNA virus 1*, +1 frameshifting is assumed but the site remains unknown. However, our comparative analysis using several recently available sequences suggested it may involve +1 P-site slippage on a conserved CCC_GAA sequence. Sites of -2 frameshifting in arteriviruses and *Trichomonas vaginalis virus 1*, and -1/+2 slippage in potyvirids, were annotated according to (1), (5) and (36).

We also added many additional coding sequence annotations to RefSeqs where homologous proteins were annotated in related viruses, and we removed some over-zealous annotation (e.g. where every ORF over 100 codons had been annotated without regard as to how non-5’-proximal ORFs would be translated). Initial multiple sequence alignments (see below) were analyzed with the gene-finding program MLOGD (1,30) to identify unannotated coding ORFs, particularly in regions previously assumed to be non-coding (e.g. non-AUG initiated upstream ORFs). MLOGD was originally developed to predict novel overlapping genes, but, in most cases, we find it to be less sensitive for this purpose than synplot2. However, MLOGD can also be used to annotate non-overlapping or partially overlapping genes. In this usage, MLOGD is conceptually similar to the Ka/Ks statistic (ratio of non-synonymous to synonymous substitutions). A small number of additional potential coding ORFs were identified based simply on the presence of a long ORF (statistical significance assessed via sequence shuffling methods similar to those described in (5)) with a plausible translation mechanism. These additional potential coding ORFs were then added to the coding sequence annotations to increase the regions accessible for synplot2 analysis (which is only applicable to the protein-coding regions of a virus genome). Note that, while important for completing the annotation of less-well studied and poorly annotated virus species, predicted coding ORFs (except ones that had been previously experimentally confirmed) were not used to evaluate synplot2 performance.

The revised coding sequence annotations are available on-line at http://www.firthlab.path.cam.ac.uk/virad.html. More than two hundred of these revisions (restricting to, but not exhausting, ones with experimental support in at least one species) have already been submitted to and incorporated into GenBank.

### Virus comparative genomics database

To generate multiple sequence alignments for each RNA virus RefSeq, we extracted and translated the (re-annotated) concatenated coding regions of each RefSeq, and used the resulting amino acid sequence as a tblastn query against a custom blast database (37). The custom blast database comprised all non-redundant RNA virus nucleotide sequences in GenBank as of 26 May 2014, excluding patent, synthetic and environmental sample sequences. Furthermore, sequences with >20 ambiguous nucleotides (e.g. ‘N's) were excluded, as were sequences with keywords ‘UNVERIFIED’, ‘STANDARD_DRAFT’, ‘VIRUS_LOW_COVERAGE’ or ‘VIRUS_AMBIGUITY’. Sequences were selected for alignment if they had ≥95% coverage and ≥75% amino acid identity to the translated concatenated coding regions of the RefSeq (in regions of gene overlap, the reading frame selected for translation was the reading frame of the longest of multiple overlapping ORFs).

Although the relevant parameter for synplot2 is nucleotide divergence, we used amino acid divergence as a proxy for sequence selection (in part because the blast searches are most effectively conducted using amino acid sequences). We chose 75% amino acid identity as the default cut-off so as to be able to detect features specific to single species or clades of closely related species, and also so that automated genome-wide alignments could be produced that mostly maintained the reading frame within coding regions. Maintenance of reading frame within coding regions is important for codon-based evolutionary analyses such as synplot2 and MLOGD. We used the alignment program code2aln version 1.2 (38), which uses both nucleotide and codon scoring metrics, to aid the generation of codon-respecting alignments. Nonetheless, insertion/deletion mutations and/or sequencing errors do occasionally lead to local shifts in reading-frame between different isolates. Thus, in each sequence, we masked regions that aligned locally out of frame with the RefSeq so that they could be excluded from the synplot2 and MLOGD analyses. Potential insertion/deletion sequencing errors in RefSeqs (e.g. where the RefSeq has a region that is locally out of frame with respect to all other sequenced isolates) were also flagged in this way. Using code2aln, sequences were aligned one by one with the RefSeq, and positions in each pairwise alignment that contained a gap character in the RefSeq were excluded. In this way, we built up a multiple sequence alignment mapped to the RefSeq genomic coordinates (if there were >200 full-length sequences, we chose 200 sequences at random; after subsequently removing duplicate sequences this typically resulted in alignments of slightly fewer than 200 sequences). The rationale for mapping sequences onto RefSeq coordinates is so that the coding sequence annotation can be defined by the RefSeq (amongst other things, making the analysis less susceptible to insertion/deletion sequencing errors in the non-reference sequences). The non-reference sequences may be thought of as informing a comparative-genomic annotation of the RefSeq.

In the interests of speed for large alignments, ClustalW version 2.1 (39) was used to generate simple neighbour-joining phylogenetic trees to use for the synplot2 and MLOGD analyses. Trees were calculated using the translated concatenated coding regions extracted from the mapped-to-RefSeq alignments described above, after excluding columns with gaps or masked regions in any sequence. Alignment divergences (mean number of nucleotide substitutions per site over a phylogenetic tree) were calculated with MLOGD (30).

In order to identify overlapping functional elements, the coding regions of multiple sequence alignments were analyzed with synplot2. Where coding sequences overlap, the reading frame corresponding to the longest of multiple overlapping ORFs was used to define synonymous codons for the synplot2 analysis. Since analysis of a genome with a sliding window involves multiple tests, a basic threshold of 0.05 / (length of coding region / window size) was annotated on plots for individual virus genomes. This is an approximate Bonferroni-like correction so that, for a given virus genome, there is an ∼5% probability that one or more regions evolving neutrally at synonymous sites would by chance register a signal beyond this threshold. To correct for multiple testing across the ∼1300 individual alignments analyzed (including separate segment alignments for viruses with multipartite genomes), more stringent criteria were required. Around half of the alignments have sufficiently low sequence divergence that it is not possible for them to register significant (post Bonferroni correction) *P*-values, even in regions of 100% synonymous-site conservation, and such alignments should not contribute to a correction for multiple testing. We used a *P*-value threshold of 10^−6^ (25-codon window size) for selecting the conserved regions reported in Supplementary Dataset S1. We chose this threshold because, summed over the alignments, there are ∼46 000 non-overlapping 25-codon windows coming from alignments with sufficient sequence divergence that 100% synonymous-site conservation in a 25-codon window would register a *P*-value of 10^−6^ or lower, giving rise to an expected probability of 46 000 × 10^−6^, i.e. ∼5% of obtaining a single false positive over the analysis of *all* alignments. It should be noted that this is a very conservative approach and use of more permissive approaches such as false discovery rate can be used to select a larger set of conserved regions.

## RESULTS

### Example applications of synplot2

**Figure 1.**
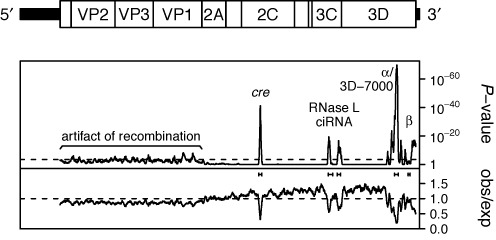
Synonymous-site variability in *Enterovirus C*. Above—map of the poliovirus genome (GenBank accession NC_002058.3; 7440 nt; a member of the species *Enterovirus C*, genus *Enterovirus*, family *Picornaviridae*). The polyprotein-coding sequence is indicated by the subdivided white box, and the 5’ and 3’ UTRs are indicated in black. Like many RNA viruses, poliovirus uses a polyprotein-expression strategy, whereby a large polyprotein is translated from a long ORF and proteolytically cleaved to produce the mature virus proteins including the structural proteins VP1, VP2, VP3 and VP4 (that form virus particles, or virions) and the nonstructural proteins such as 3C (the viral protease) and 3D (the viral RNA-dependent RNA polymerase, or RdRp). Below—analysis of synonymous-site variability in an alignment of 198 *Enterovirus C* sequences. The lower panel (obs/exp) indicates the relative amount of synonymous-site variability as represented by the ratio of the observed number of synonymous substitutions to the expected number, in a 15-codon sliding window. The upper panel shows the corresponding *P*-value (note that *P*-values cannot be compared directly between plots as larger and more diverse alignments provide more statistical power). The dashed line represents a *P*-value of 0.05 / (polyprotein length / window size)—an approximate Bonferroni-like correction for multiple testing; i.e. there is an ∼5% probability that one or more regions evolving neutrally at synonymous sites could by chance register a signal above the dashed line. Functional non-coding RNA elements embedded within the polyprotein-coding sequence—namely the *cre*, the RNaseL ciRNA (two components) and the α/3D-7000 and β elements (see main text)—are labelled and the corresponding sequence regions are indicated below the *P*-value plot. Here, recombination occurring between the regions encoding the structural and nonstructural proteins, and the consequently incorrect assumption of a uniform phylogenetic tree across the alignment, has led to a signal of slightly reduced variability in the 5’ part of the polyprotein-coding sequence.

**Figure 2.**
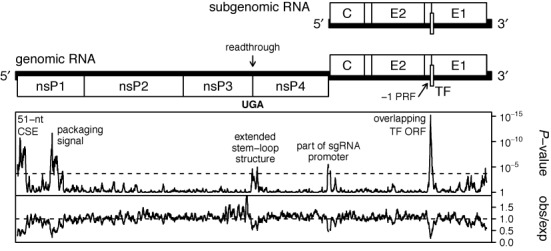
Synonymous-site variability in VEEV. Top—map of the VEEV genome (GenBank accession NC_001449.1; 11 444 nt; genus *Alphavirus*, family *Togaviridae*). The nonstructural polyproteins nsp1-nsp2-nsp3 and, via programmed stop codon readthrough, nsp1-nsp2-nsp3-nsp4 are translated from the genomic RNA. The structural polyproteins C-E3-E2–6K-E1 and, via programmed -1 ribosomal frameshifting (-1 PRF), C-E3-E2-TF, are translated from a sgRNA that is produced during viral infection. Below—analysis of synonymous-site variability in an alignment of 123 VEEV sequences (15-codon sliding window; see the caption to Figure 1 for details). Functional elements overlapping the polyprotein-coding sequences are annotated (see main text). Note that a similar plot, but based on a different sequence alignment, for ORF1 only has been published previously (45).

**Figure 3.**
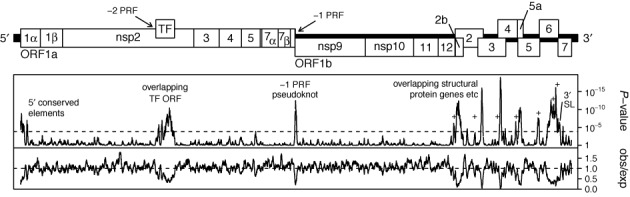
Synonymous-site variability in PRRSV. Top—map of the PRRSV genome (GenBank accession NC_001961.1; 15 428 nt; genus *Arterivirus*, family *Arteriviridae*). The nonstructural polyproteins nsp1–8 and, via programmed -1 ribosomal frameshifting (-1 PRF) nsp1–12 are translated from the genomic RNA. The structural proteins, encoded by ORFs 2b, 2, 3, 4, 5, 5a, 6 and 7, are translated from a series of 3’-coterminal sgRNAs (not shown) that are produced during virus infection. The TF ORF is translated via -2 PRF, resulting, after proteolytic cleavage, in the production of an nsp2TF ‘transframe’ protein. Below—analysis of synonymous-site variability in an alignment of 194 PRRSV type II (‘North American’ genotype) sequences (15-codon sliding window; see the caption to Figure 1 for details). Overlapping features are annotated (see main text). Transcription-regulatory sequences (TRSs) are indicated with ‘+'s. Note that a similar plot, but based on a different sequence alignment, for ORF1a only has been published previously (5).

**Figure 4.**
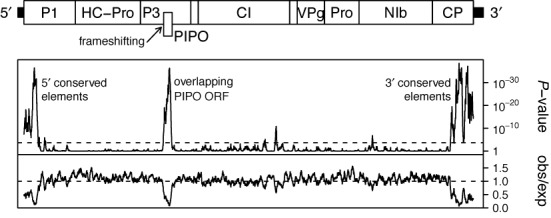
Synonymous-site variability in TuMV. Top—map of the TuMV genome (GenBank accession NC_002509.2; 9835 nt; genus *Potyvirus*, family *Potyviridae*). Most gene products are encoded in a single ORF, but low level frameshifting in the P3 region gives rise to a truncated P1/HC-Pro/P3N-PIPO polyprotein from which the ‘transframe’ P3N-PIPO protein is derived after proteolytic cleavage. Below—analysis of synonymous-site variability in an alignment of 196 TuMV sequences (15-codon sliding window; see the caption to Figure 1 for details). Besides the overlapping PIPO ORF, conserved overlapping elements are detected at the 5’ and 3’ termini of the polyprotein-coding sequence (see main text).

**Figure 5.**
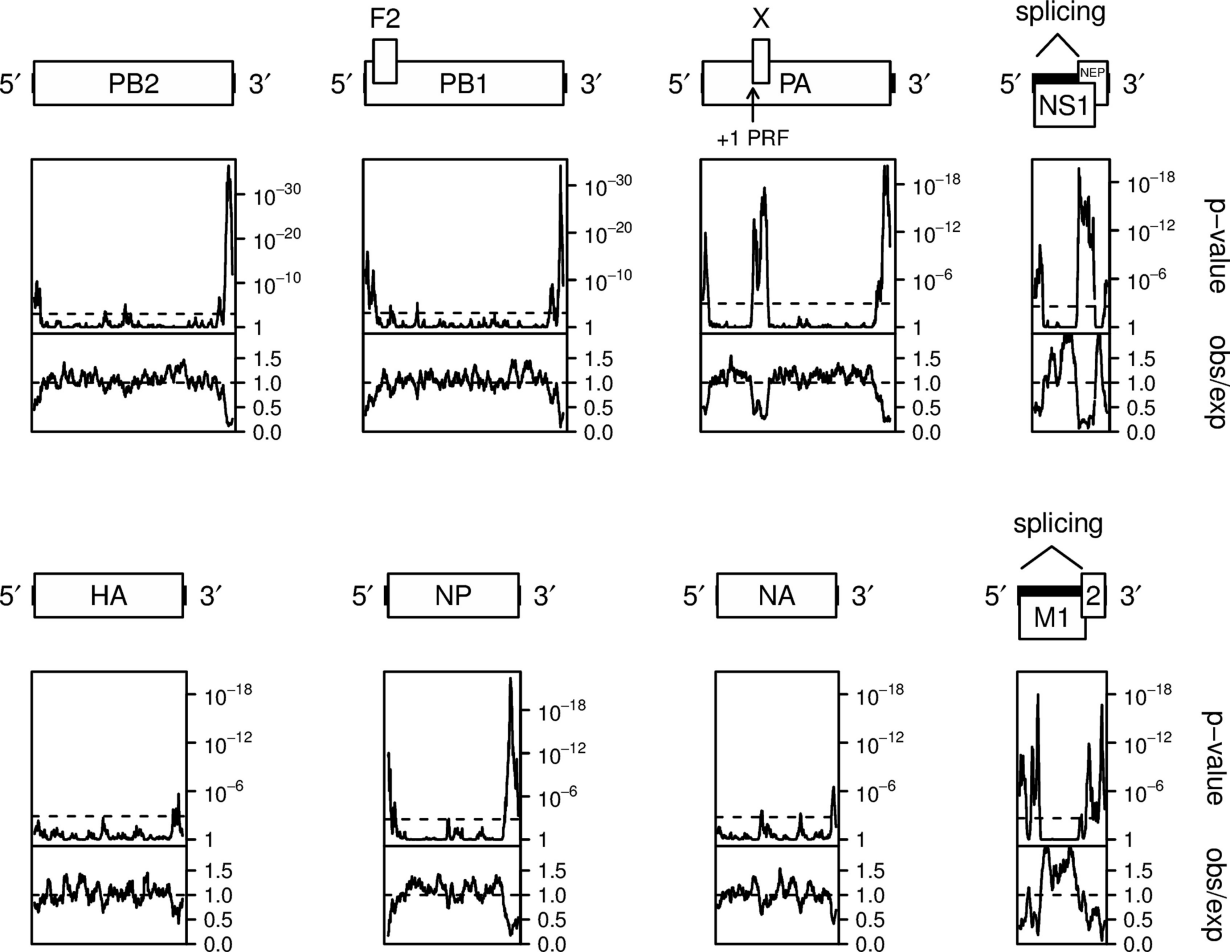
Synonymous-site variability in IAV. The IAV genome comprises eight separate segments. For each of segments 7 and 8, two proteins are encoded, with one being expressed from a spliced transcript. The overlapping X ORF is expressed as a fusion (PA-X) with the N-terminal region of PA via programmed +1 ribosomal frameshifting (+1 PRF). The analysis of synonymous-site variability is based on alignments of ∼180–200 sequences per segment (15-codon sliding window; see the caption to Figure 1 for details). Regions of reduced synonymous-site variability correspond to terminal packaging signals and the PA/X and NS1/NEP dual-coding regions. Note that *P*-values cannot be directly compared between plots as more diverse alignments provide more statistical power. The obs/exp values can, however, be compared between plots. Note that a similar plot, but based on a different sequence alignment, for the PA segment has been published previously (6).

First we applied synplot2 (see Materials and Methods) to a number of well-studied RNA viruses for which large numbers of sequenced isolates are available. Results for *Enterovirus C* (i.e. poliovirus and relatives), *Venezuelan equine encephalitis alphavirus* (VEEV), Porcine reproductive and respiratory syndrome arterivirus (PRRSV), Turnip mosaic potyvirus (TuMV) and IAV are shown in Figures 1–5. These viruses contain a number of known functional elements embedded within the coding sequences (40). For *Enterovirus C* these include the *cre* (*cis*-acting replication element) (41), the RNase L ciRNA (competitive inhibitor of RNase L) (42) and the α/3D-7000 element (7,10). The synplot2 analysis clearly and easily reveals all of these overlapping functional elements (Figure 1). An additional previously characterized feature, the β stem-loop (7), was not well-detected, but regions of statistically significantly reduced variability at synonymous sites were observed immediately adjacent to β, besides elsewhere within the 3D-encoding region. For VEEV, the analysis revealed all of the known functional elements—the 51-nt CSE (conserved sequence element) (43), the packaging signal (44,45), an extended stem-loop structure that mediates stop-codon readthrough (24), the 5’ end of the sgRNA promoter (46) and the overlapping TF ORF and associated -1 frameshift stimulating elements within the 6K region (2) (Figure 2).

The synplot2 analysis of PRRSV revealed regions of reduced synonymous-site variability associated with the overlapping TF ORF that is accessed via -2 frameshifting (5), the RNA pseudoknot structure that directs -1 frameshifting between ORFs 1a and 1b (31), the regions where the different structural-protein-coding ORFs overlap each other (4,47,48), the stem-loop structure within ORF7 (49) and potentially additional 5’-proximal and 3’-proximal elements. The negative-sense templates of PRRSV sgRNAs are synthesized using a discontinuous transcription mechanism that results in an anti-leader sequence, templated by the very 5’ end of the genomic RNA, being appended to the 3’ end of each negative-sense sgRNA template, which is subsequently copied to a 5’ leader sequence on each sgRNA (50). The polymerase skipping occurs at transcription-regulatory sequences (TRSs) positioned upstream of ORFs 2, 3, 4, 5, 6 and 7 (‘+'s in Figure 3). Although the TRSs themselves comprise just a few nucleotides (UAACC or closely related sequences in PRRSV), synplot2 revealed regions of reduced synonymous-site variability at or just upstream of most TRS sites. The analysis of TuMV clearly revealed the overlapping *pipo* ORF (1) but also revealed extensive regions of reduced variability at synonymous sites at the 5’ and 3’ ends of the polyprotein-coding sequence (some of the latter may correspond to functional elements previously identified in related potyviruses) (51,52). Finally the analysis of IAV revealed, as observed previously using different methods (22), reduced synonymous-site variability in regions towards the termini of most segments which is thought to correspond to packaging signals, in a central region of the M1 ORF in segment 7, and in the dual-coding regions where the NS1 and NEP ORFs overlap, and where the PA and X ORFs overlap. In accordance with previous comments (53) we observed relatively weak purifying selection on the overlapping PB1-F2 ORF (54) in pan-IAV alignments.

As external objective test sets, we used a list of experimentally verified overlapping genes in RNA viruses from (55) and a list of experimentally characterized overlapping non-coding elements from (40). To detected overlapping genes we used a sliding window size of 45 codons, while to detect overlapping non-coding elements—which are often much smaller features—we used a sliding window size of 15 codons. A highly conservative threshold of *P* ≤ 10^−6^ (designed to allow approximately one false positive over an analysis of all RNA virus genomes; see Materials and Methods) was used to designate features as ‘detected’. For each feature, we selected a well-sequenced representative virus species containing the feature. Features for which all of the relevant species-based alignments lacked sufficient diversity to allow synplot2 to detect even a 60% reduction in synonymous-site variability (i.e. obs/exp = 0.4) at *P* ≤ 10^−6^ in a window of the given size were not used, as the purpose of the exercise was to determine whether there are known features that synplot2 fails to detect even when provided with sufficient sequence diversity. (Note that the threshold for discarding low diversity alignments is a mean-over-genome statistic and may not correspond precisely to the *P*-value for obs/exp = 0.4 in a specific window.)

Synplot2 detected 20/21 overlapping genes (Table 1) and 16/17 overlapping non-coding features (Table 2). The non-detections (i.e. *P* > 10^−6^) comprised the overlapping VP5 gene in *Infectious pancreatic necrosis virus* (genus *Aquabirnavirus*) which appears to be subject to relatively weak purifying selection (high obs/exp relative to other dual-coding regions; Table 1), and the cardiovirus *cre* where the *P*-value (3.4 × 10^−6^) was just above threshold. Thus, given sufficient sequencing depth, synplot2 appears to easily discover most known functional elements embedded within RNA virus coding sequences.

**Table 1.** Synplot2 results for representative overlapping genes

Taxon	RefSeq	Gene overlap	Genomic location (nt)	obs/exp	*P*-value	Detected
*Picornaviridae*, *Cardiovirus*, *Theilovirus*	NC_001366.1	L/L*	1081–1551	0.16	1.4 × 10^−14^	yes
*Arteriviridae*, *Arterivirus*, *PRRSV*	NC_001961.1	GP2/GP3	12696–12843	0.56	6.4 × 10^−12^	yes
		GP3/GP4	13241–13460	0.44	2.5 × 10^−18^	yes
*Bromoviridae*, *Cucumovirus*, *Cucumber mosaic virus*	NC_002035.1	ORF2a/2b	2419–2660	0.18	2.9 × 10^−19^	yes
*Hepeviridae*, *Hepevirus*, *HEV*	NC_001434.1	CP/ORF3	5123–5453	0.08	7.0 × 10^−206^	yes
*Betaflexiviridae*, *Capillovirus*, *Apple stem grooving virus*	NC_001749.2	replicase-CP/MP	4787–5749	0.06	2.1 × 10^−20^	yes
*Betaflexiviridae*, *Trichovirus*, *Apple chlorotic leaf spot virus*	NC_001409.1	MP/CP	6784–7100	0.07	4.0 × 10^−31^	yes
*Alphaflexiviridae*, *Potexvirus*, *Pepino mosaic virus*	NC_004067.1	TGB2/TGB3	5340–5488	0.35	5.7 × 10^−9^	yes
*Sobemovirus*, *Rice yellow mottle virus*	NC_001575.2	replicase/CP	3447–3607	0.21	4.2 × 10^−10^	yes
*Nodaviridae*, *Betanodavirus*, *Striped jack nervous necrosis virus*	NC_003448.1	replicase/B2	2756–2983	0.07	1.3 × 10^−15^	yes
*Tombusviridae*, *Tombusvirus*, *Tomato bushy stunt virus*	NC_001554.1	MP/p19	3888–4406	0.19	1.3 × 10^−22^	yes
*Birnaviridae*, *Aquabirnavirus*, *Infectious pancreatic necrosis virus*	NC_001915.1	VP5/VP2	120–514	0.65	1.6 × 10^−4^	no
*Birnaviridae*, *Avibirnavirus*, *Infectious bursal disease virus*	NC_004178.1	VP5/VP2	130–533	0.20	1.1 × 10^−20^	yes
*Reoviridae*, *Orthoreovirus*, *Mammalian orthoreovirus 3*	NC_004277.1	σ1/σ1s	71–433	0.29	1.2 × 10^−12^	yes
*Totiviridae*, *Totivirus*, *Saccharomyces cerevisiae virus L-A*	NC_003745.1	gag/pol	1964–2072	0.19	2.1 × 10^−11^	yes
*Bunyaviridae*, *Orthobunyavirus*, *La Crosse virus*	NC_004110.1	N/NSs	101–379	0.31	5.0 × 10^−28^	yes
*Paramyxoviridae*, *Morbillivirus*, *Measles virus*	NC_001498.1	P/C	1829–2389	0.11	2.5 × 10^−12^	yes
		P/V	2499–2705	0.16	1.2 × 10^−11^	yes
*Paramyxoviridae*, *Respirovirus*, *Human parainfluenza virus 3*	NC_001796.2	P/C	1794–2393	0.06	5.4 × 10^−16^	yes
		P/V	2505–2903	0.49	2.8 × 10^−7^	yes
*Paramyxoviridae*, *Rubulavirus*, *Mumps virus*	NC_002200.1	P/V	2442–2653	0.11	2.8 × 10^−13^	yes

The obs/exp and *P*-values (see Figure 1 caption for details) are reported for the highest scoring 45-codon window in the region of overlap. Elements with *P* ≤ 10^−6^ were classified as ‘detected’.

**Table 2.** Synplot2 results for representative overlapping non-coding elements

Taxon	RefSeq	Feature	Genomic location (nt)	obs/exp	*P*-value	Detected
*Picornaviridae*, *Enterovirus*, *Enterovirus C*	NC_002058.3	*cre*	4446–4502	0.31	7.3 × 10^−42^	yes
		RNase L ciRNA 5’ part	5741–5824	0.55	4.4 × 10^−20^	yes
		RNase L ciRNA 3’ part	5906–5969	0.61	2.1 × 10^−17^	yes
*Picornaviridae*, *Enterovirus*, *Human rhinovirus 14*	NC_001490.1	*cre*	2330–2403	0.54	1.6 × 10^−10^	yes
*Picornaviridae*, *Cardiovirus*, *Saffold virus*	NC_009448.2	*cre*	1509–1589	0.60	3.4 × 10^−6^	no
*Picornaviridae*, *Hepatovirus*, *Hepatitis A virus*	NC_001489.1	*cre*	5948–6057	0.21	1.2 × 10^−10^	yes
*Picornaviridae*, *Parechovirus*, *Human parechovirus*	NC_001897.1	*cre*	1375–1425	0.39	7.3 × 10^−17^	yes
*Flaviviridae*, *Hepacivirus*, *Hepatitis C virus*	NC_004102.1	5’ stem-loops	342–508	0.10	2.3 × 10^−60^	yes
		3’ upstream element	9107–9123	0.42	1.0 × 10^−27^	yes
		3’ stem-loops V+VI	9215–9313	0.17	1.5 × 10^−45^	yes
*Togaviridae*, *Alphavirus*, *VEEV*	NC_001449.1	51-nt CSE	134–184	0.36	7.0 × 10^−9^	yes
		packaging signal	875–1148	0.21	2.2 × 10^−12^	yes
*Arteriviridae*, *Arterivirus*, *PRRSV*	NC_001961.1	frameshift site and 3’ pseudoknot	7689–7758	0.21	3.4 × 10^−13^	yes
		ORF7 stem-loop	14933–14959	0.42	4.2 × 10^−7^	yes
*Caliciviridae*, *Norovirus*, *Murine norovirus*	NC_008311.1	ORF1 5’ stem-loop	8–60	0.00	4.8 × 10^−14^	yes
		sgRNA promoter stem-loop	5018–5045	0.05	1.5 × 10^−17^	yes
		ORF3 stem-loop	7166–7302	0.36	1.5 × 10^−9^	yes

The obs/exp and *P*-values (see Figure 1 caption for details) are reported for the highest scoring 15-codon window in the region of overlap. Elements with *P* ≤ 10^−6^ were classified as ‘detected’. Nucleotide coordinates are only approximate as the precise boundaries of non-coding elements are often poorly defined.

### Sensitivity to window size, alignment depth and feature type

**Figure 6.**
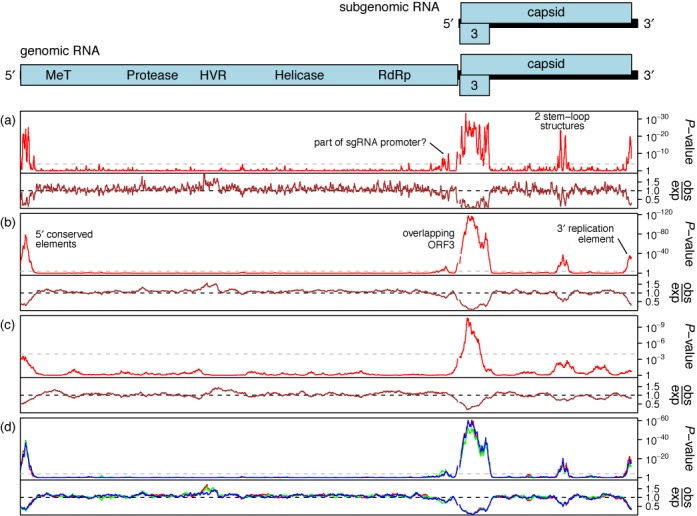
Synonymous-site variability in HEV. Top—map of the HEV genome (GenBank accession NC_001434.1; 7176 nt; genus *Hepevirus*, family *Hepeviridae*). ORF1 encodes the nonstructural protein domains which are translated from the genomic RNA. Both ORF3 (which encodes an accessory protein) and ORF2 (which encodes the capsid protein) are translated from a sgRNA; ORF3 is in the +1 reading frame with respect to ORF2. **(a)** Analysis of synonymous-site variability in an alignment of 192 HEV sequences (see the caption to Figure 1 for details) using a 5-codon sliding window to obtain a high-resolution map of overlapping functional elements. Here the two stem loops in the middle of the capsid-coding ORF are clearly resolved as separate features. Small breaks in the *P*-value and obs/exp lines at the starts of ORF3 and ORF2 indicate, respectively, the intergenic region between ORFs 1 and 3, and the junction between overlapping ORFs 2 and 3 where a partial codon (i.e. 2 nt) has been omitted from the calculations. **(b)** Analysis of the same alignment with a 25-codon sliding window. Larger sliding window sizes are more useful for identifying extended overlapping features such as overlapping genes. Here, the overlapping ORF3 is clearly revealed. **(c)** Provided the sequences are sufficiently divergent, even an alignment of two sequences (here, GenBank accession numbers NC_001434.1 and AB161718.1; 75-codon sliding window) can reveal extended features such as the overlapping ORF3. **(d)** The 192-sequence alignment was arbitrarily split into three 64-sequence alignments, and each alignment was analyzed separately (red, blue, green lines). All three alignments produce similar results. Note that a plot similar to (a), but based on a different sequence alignment, has been published previously (28).

The sliding window size may be adjusted to suit the available sequence data (‘deeper’ alignments allow statistically significant results to be achieved for smaller window sizes than ‘shallow’ alignments) and for the type of feature under consideration (maximum sensitivity is achieved when the window is similar in size to the feature, e.g. 5–15 codons for a typical RNA stem-loop structure, but 40–200 codons for a typical overlapping protein-coding ORF). Figure 6a and b show analyses of HEV with a 5-codon window (which clearly distinguishes the two adjacent stem-loop structures in an internal region of ORF2) and a 25-codon window (which provides a much stronger signal for the overlapping ORF3).

If sequences are too divergent then there will be too few synonymous positions for synplot2 to assess. Conversely, if sequences have very high identity, then there may be too few variations for synplot2 to distinguish the signature of purifying selection, though this issue can be circumvented if sufficiently many low-divergence but non-identical sequences are available. In between these two extremes, synplot2 can produce useful results even with just two sequences. Figure 6c shows an example of synplot2 applied to an alignment of just two divergent HEV isolates—the GenBank RefSeq NC_001434.1 and GenBank accession number AB161718.1 (83% amino acid identity, 75% nucleotide identity, in the coding regions), using a 75-codon sliding window. Even with just two sequences, synplot2 clearly detects the overlapping ORF3. However, much higher resolution is attainable for the larger alignment used for Figure 6a (192 sequences).

**Figure 7.**
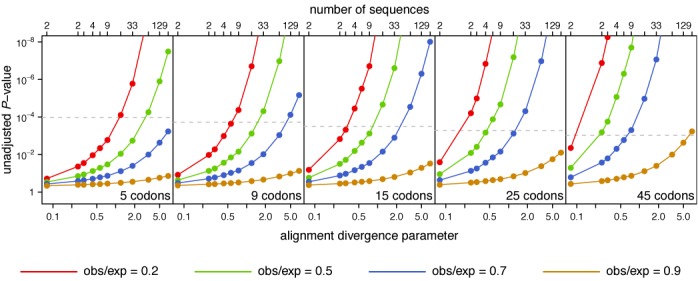
Synplot2 sensitivity as a function of alignment diversity for different window sizes and levels of purifying selection. Synplot2 was applied to alignments of different numbers of HEV sequences randomly selected from the 192-sequence alignment used in Figure 6 (see text). For each alignment, the average *P*-value (filled circles) for the alignment was calculated for a given sliding window size (indicated at bottom right of each panel) and hypothetical level of reduced synonymous-site variability (obs/exp), a calculation which depends only on the null model. The ‘divergence’ of each alignment (measured by the mean number of nucleotide substitutions per site over the phylogenetic tree) is indicated at the bottom, and the number of sequences in each alignment is indicated at the top. The latter may be illustrative but is not a good indicator of alignment diversity, e.g. two 2-sequence alignments were used, one with an alignment divergence of 0.08 nucleotide substitutions per site and the other with an alignment divergence of 0.25 nucleotide substitutions per site. In each panel, the dashed line indicates a *P*-value of 0.05 corrected for multiple testing within the HEV genome (i.e. 0.05 / (HEV coding region size / window size); see Figure 1 caption). Larger features, lower obs/exp and higher alignment divergence correlate with increased synplot2 sensitivity.

To quantify synplot2 sensitivity as a function of alignment diversity, and feature size and level of purifying selection, we constructed alignments of the HEV RefSeq, NC_001434.1, together with 1, 2, 4, 8, 16, 32, 64, 128 or 191 sequences randomly selected from the initial HEV alignment. Since the randomly selected 2-sequence alignment was quite divergent (75% nucleotide identity), we also manually selected a low-divergence 2-sequence alignment (92% nucleotide identity). This provided a range of alignment divergences ranging from 0.08 to 6.85 nucleotide substitutions per site over the phylogenetic tree. For each alignment, we calculated the mean *P*-value across the alignment that would be achieved for various levels of reduced synonymous-site variability (i.e. obs/exp), and for a range of sliding window sizes (Figure 7). The *P*-value for a sliding window of size 9 codons and obs/exp = 0.7 represents the ability of synplot2 to detect an overlapping feature that leads to a 30% decrease in synonymous-site variability in a 9-codon window. As can be seen from Figures 1–6 and Tables 1 and 2, both overlapping genes and overlapping non-coding elements are frequently associated with >50% reductions in synonymous-site variability. For typical one-off analyses (e.g. *P*-value threshold of 0.05 with multiple testing corrections based on a single HEV-sized genome), an alignment divergence of ∼0.25 may be sufficient to identify features of size ≥45 codons, such as many overlapping genes (Figure 7, panel 5). For non-coding elements, smaller window sizes (e.g. 7–25 codons) are generally more appropriate. For a 15-codon sliding window, an alignment divergence of ∼0.85 may be sufficient to identify overlapping features (Figure 7, panel 3). Window sizes of 5 codons are only useful for the most highly sequenced species such as HEV, but are becoming accessible for more species as sequence databases continue to grow. Functional elements even smaller than 5 codons may be detected within 5-codon windows given sufficient reduction in synonymous-site variability, but window sizes smaller than 5 codons are best avoided due to fluctuations in sensitivity as a result of different levels of codon degeneracy (e.g. conserved columns of AUG Met or UGG Trp codons provide no synonymous site variation signal).

### Sensitivity to choice of sequences and phylogenetic tree

To show that synplot2 is not sensitive to the non-uniqueness inherent in the choice of sequence pairs covering the phylogenetic tree (see Materials and Methods), nor the arbitrary selection of sequences for species with >200 sequences available, we randomly split the 192-sequence HEV alignment into three 64-sequence alignments. Figure 6d shows the results of applying synplot2 to each of the three alignments. All three alignments produced similar results, indicating that the plots are not greatly affected by the phylogenetic tree, nor, for large alignments, the exact choice of sequences.

### Sensitivity to recombination

Recombination is a common phenomenon in many RNA viruses (56). Alignments containing recombinants typically contain regions where variability (both synonymous and non-synonymous) is reduced relative to the genome average. Potentially this can be problematic for the synplot2 analysis, where the divergence parameters and phylogenetic tree are assumed to be constant for the whole region analyzed. In the work presented here, we have not checked for nor removed recombinants as we have only rarely found recombination to be a significant issue for synplot2 analyses. Recombination tends to result in distinctive plateaux in the synplot2 *P*-value plots (an example is purposefully included in Figure 1) allowing problematic cases to be identified and reanalysed with recombinants excluded. Alternatively, standard recombination software can be used to pre-screen alignments for recombinants (57). A complementary strategy is to generate alignments for the coding regions of individual proteins (or separate regions of polyproteins) as fixed recombinations occur much more frequently between than within protein-coding domains. We have found synplot2 to be quite effective on alignments as short as 100 codons in length.

### Application of synplot2 to NCBI virus RefSeqs

Next, we generated sequence alignments for all NCBI RNA virus RefSeqs (see Materials and Methods) and analyzed the coding regions from each alignment with synplot2. A database of the synplot2 results for all alignments, together with the MLOGD analysis showing the coding potential in each reading frame, and the positions of stop codons throughout the alignment in each reading frame (for identification of conserved ORFs), is available on-line at http://www.firthlab.path.cam.ac.uk/virad.html. Synplot2 results for 72 representative RNA viruses are shown in Supplementary Figures S1–S16. Statistics illustrating the diversity and synplot2 sensitivity of each sequence alignment used in the figures, tables and supplementary figures are included in Supplementary Table S1. Predicted novel protein-coding ORFs (based on the MLOGD or synplot2 analyses, and/or the presence of a long ORF and plausible translation mechanism) are listed in Supplementary Table S2 (these will be discussed further in future work). Potential and, to our knowledge, previously undescribed genes predicted on the basis of the synplot2 analysis include identifications in *Mamastrovirus* genogroups III (e.g. rodent, porcine, and bovine isolates) and IV (e.g. human astroviruses MLB1, 2 and 3) (potentially, but not necessarily, related to the previously published predicted overlapping gene in genogroup I astroviruses; (58)), cosaviruses, *Sacbrood virus* and some related iflaviruses, the VP3-encoding segment of *Rotavirus G* (likely conserved also in *Rotavirus B*), the S segment of *Capsicum chlorosis virus* and related tospoviruses, and the VP2-encoding segment of *Aquareovirus A*.

Overlapping genes often lead to extended regions of conservation (e.g. Figures 3–6; Supplementary Figures S5.3, S5.5, S6.1, S6.3, S6.5, S7.4, S8.4, S16.1). We frequently observed conservation in places expected for -1 frameshift stimulatory elements (e.g. Figure 3; Supplementary Figures S1.1, S1.6, S6.1) (31,59), readthrough stimulatory elements (e.g. Figure 2; Supplementary Figures S2.2, S6.1, S6.3) (24,60), reinitiation stimulatory elements (e.g. Supplementary Figures S5.4 and S5.5) (61) and sgRNA promoters (e.g. Figure 2; Supplementary Figures S1.1, S5.4, S5.6, S6.8, S7.1, S7.2, S7.4) (62–65). Other conserved regions associated with known internal elements were also observed. Examples include the picornavirus *cre* element, whose location in the genome varies between different picornavirid species (Supplementary Figures S4.1, S4.2, S4.3, S4.5, S4.6, S4.7) (66); distal elements that regulate sgRNA synthesis in the tombusviruses (Supplementary Figure S6.3) (67,68) and alphanodavirus RNA1 (Supplementary Figure S5.7) (69); a 5’ replicational element in alphanodavirus RNA1 (70); 3’ replicational elements in HEV (Figure 6) (71) and in potyviruses (Supplementary Figures S8.1 and S8.2) (51,52); predicted 3’ elements in astroviruses (Supplementary Figure S1.1) (72); the packaging signal in *Saccharomyces cerevisiae virus L-A* (Supplementary Figure S10.1) (73); 5’ replicational and/or translation enhancer elements in *Dengue virus* and *Japanese encephalitis virus* (Supplementary Figures S3.1 and S3.2) (74–76); 5’ and 3’ conserved regions in the coding regions of most reovirid genome segments (Supplementary Figures S11.1, S12.1, S13.1) (77–79); a large 3’-proximal stem-loop structure in *Murine norovirus* (Supplementary Figure S5.3) (80); an uncharacterized element in *Feline calicivirus* (Supplementary Figure S5.4) (64) and numerous regions of conservation in *GB virus C* (Supplementary Figure S3.4) (20).

However, a large number of (to our knowledge) previously uncharacterized conserved regions were also observed. Conserved regions at or near the 5’ and/or 3’ ends of the genomic coding regions were observed in many taxa, including potyviruses (Supplementary Figures S8.1 and S8.4), nepoviruses (Supplementary Figure S9.3), fabaviruses (Supplementary Figure S9.4), betaflexiviruses (Supplementary Figures S6.2, S6.6, S6.8, S7.1, S7.3, S7.4), cucumoviruses (Supplementary Figure S9.2), closteroviruses (Supplementary Figure S7.5), totivirids (Supplementary Figures S10.2, S10.3, S10.4) and birnaviruses (Supplementary Figures S10.5 and S10.6). Other conserved regions were observed within ORF6 of PRRSV (Figure 3) and in the 3’ ORF (RdRp ORF) of the totivirids *Leishmania RNA virus 1* (Supplementary Figure S10.4) and *Trichomonas vaginalis virus 1* (Supplementary Figure S10.3). Three conserved regions of note occurred in segment 10 of *Bluetongue virus* (‘?’ in Supplementary Figure S12.1), segment S4 of *Mammalian orthoreovirus 3* (‘?’ in Supplementary Figure S13.1) and RNA2 of betanodaviruses (‘?’ in Supplementary Figure S5.6). Each of these conserved regions coincides with a nearly conserved absence of stop codons in an alternative reading frame (+2 frame relative to the main coding ORF for the orthoreovirus and +1 frame for the other two), and may therefore indicate a novel overlapping coding ORF. In all three cases there is a suitable conserved AUG codon for AUG-initiated translation of the overlapping ORF. However the strong context of the AUG initiation codon of the main ORF, besides additional AUG codons in some sequences upstream of the AUG codon of the overlapping ORF, would be expected to inhibit leaky scanning, thus making the translational mechanism for these three potential novel ORFs uncertain. Further, the conservation signal in *Bluetongue virus* might also be compatible with a -1 frameshift to access a shorter overlapping ORF in the -1/+2 reading frame. Alternatively these conserved regions may represent novel non-coding elements.

Interestingly, single-stranded negative-sense RNA viruses tend to exhibit evidence for relatively few overlapping functional elements (e.g. Supplementary Figures S15.2 and S15.3). Reduced synonymous-site variability was observed at the 5’ end of the coding regions of some bunyavirid genome segments (e.g. Supplementary Figures S14.1 and S14.2), and at both ends of orthomyxovirus genome segments (e.g. Figure 5), likely corresponding to sorting and packaging signals. However, most regions of reduced synonymous-site variability corresponded to regions of gene overlap—e.g. in orthomyxoviruses (e.g. Figure 5), bunyavirids (segment S) (e.g. Supplementary Figures S14.1 and S14.2), paramyxoviruses (e.g. Supplementary Figures S16.1, S16.2, S16.3, S16.4), *Borna disease virus* and ebolaviruses (Supplementary Figure S15.1). The apparent paucity of RNA structural elements may be due to a characteristic of negative-sense RNA virus replication, namely that the genomic and antigenomic RNAs are always found bound to multiple copies of the nucleocapsid protein (81), thus reducing their capacity to engage in RNA:RNA interactions. Indeed, although terminal sequences base-pair in bunyavirids (82,83), and stem-loop structures may play roles in transcription termination and translation (84,85), extensive RNA structure has been found to be generally lacking in negative-sense RNA viruses (86).

For generic identification of regions of reduced synonymous-site variability, we identified codon positions in alignments where the synplot2 *P*-value for a 25-codon window centred on that codon position was ≤ 10^−6^. For very large and diverse alignments, *P*-values can become significant even for fairly modest reductions in synonymous-site variability; so, in order to focus only on regions with strong purifying selection (a proxy for the degree of functional importance), we further selected only codon positions where the ratio of the observed number to the expected number of synonymous substitutions in the 25-codon window was ≤ 0.65. Adjacent codon positions satisfying these conditions were merged into regions, and adjacent regions were merged if the gap between them was ≤ 24 codons (the rationale for this is that each selected codon position actually represents the midpoint of a 25-codon window). More than 700 such regions of low synonymous-site variability are listed in Supplementary Dataset S1.

## DISCUSSION

We have analyzed hundreds of RNA virus species to reveal hundreds of regions with statistically significantly reduced variability at synonymous sites, indicative of overlapping functional elements. While related methods have been developed by others and used for the analysis of selected virus genomes, including *Hepatitis C virus* (87–90), *GB virus C* (20), some potyvirids (21), pestiviruses and enteroviruses (89), IAV (22), caliciviruses (64), *Human immunodeficiency virus 1* (91,92), *Rotavirus A* (93) and HEV (94), most of this previous work either does not incorporate phylogeny (resulting in a reduced signal) and/or does not involve calculation of *P*-values (so that statistically significant overlapping features cannot easily be distinguished from random variation). We have applied our analysis to hundreds of virus species. Our results also illustrate the utility of the synplot2 software which is easily applicable to nearly any coding-sequence alignment, including alignments of DNA virus, bacterial or eukaryotic coding sequences (95,96). It should be noted that, for eukaryotes, many internal regions of reduced synonymous-site variability are associated with exon-exon junctions and may correspond to exonic splicing enhancer sequences (97–99), rather than overlapping genes or RNA structural elements.

**Figure 8.**
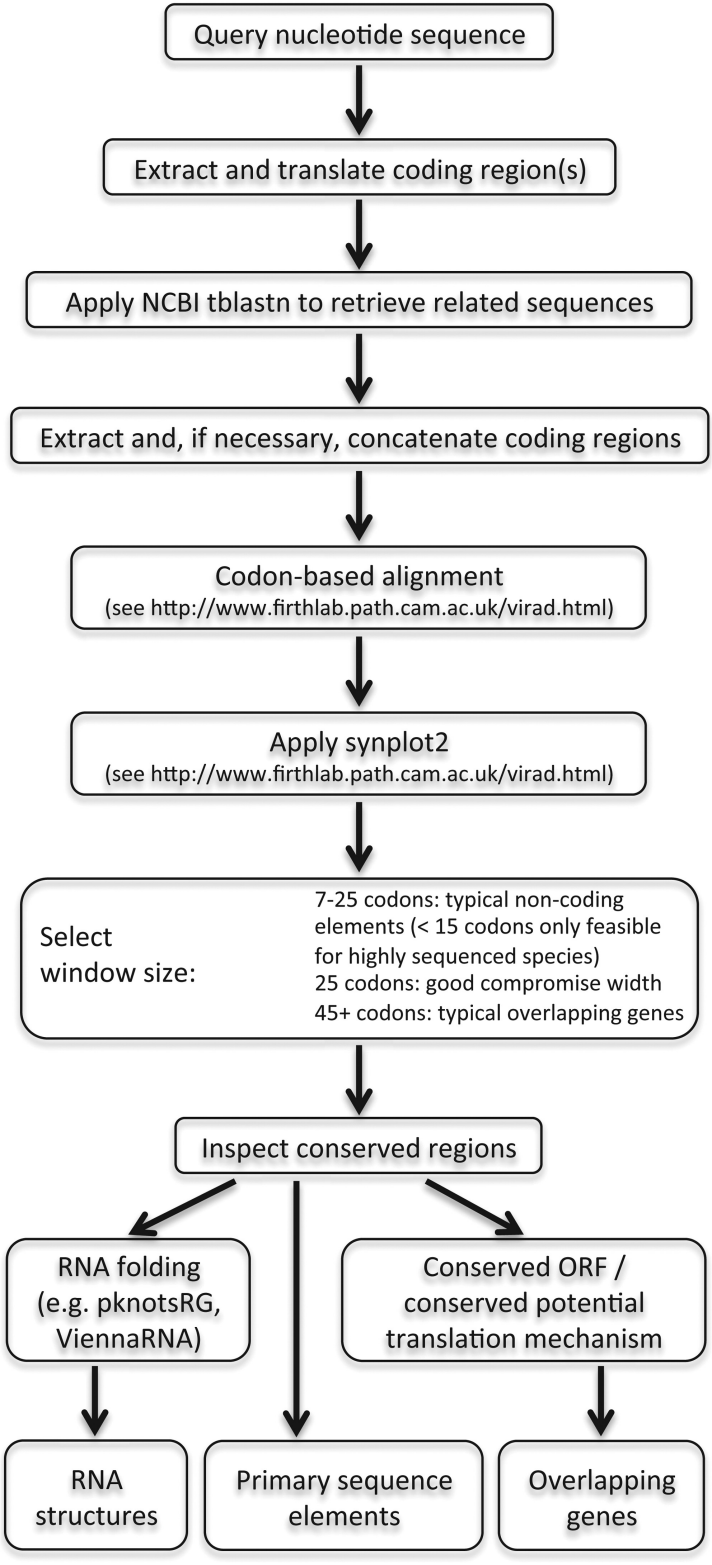
Example workflow for synplot2 analysis. The synplot2 analysis requires a codon-based multiple sequence alignment and a single parameter, the sliding window size. Maximum signal for a specific feature will be achieved when the window size is equal to the size of the feature. A window size of 45 codons works well for many overlapping genes. Functional RNA structural elements often fall in the range 7–25 codons; however window sizes below ∼9–13 codons can produce noisy results and are best avoided except for the most highly sequenced species; a window size of 15 codons works well for many cases. Functional primary sequence elements (for example paramyxovirid transcriptional slippage sites, PAX-like +1 frameshift sites, protein binding sites and nidoviralid TRSs) are often <5 codons and might escape detection, though they are often associated with other features (overlapping genes in the first two cases, potential regulatory sequences in the last) that may be detected.

Although synonymous site conservation cannot itself distinguish between overlapping coding sequences and overlapping non-coding functional elements, subsequent inspection of the corresponding sequences can reveal the presence of a conserved open reading frame and a conserved potential translation mechanism (e.g. sequence motifs associated with ribosomal frameshifting) which can indicate the presence of an overlapping coding sequence. Alternatively, inspection with RNA folding software such as the alignment-based methods in the ViennaRNA package (100) can reveal the presence of compensatory substitutions (i.e. paired substitutions that preserve predicted base-pairings) that can indicate the presence of a functional RNA structural element. An example workflow is illustrated in Figure 8. Given sufficient alignment depth and divergence, these signals may be unambiguous. In other cases they can still provide predictions for experimental investigation.

For a large number of important RNA viruses, there are many sequenced isolates often with inter-isolate nucleotide divergences ranging up to 10–30%. For these viruses, synplot2 can be used on single-species alignments to identify even species-specific features. Where within-species data is limited, one may use alignments of sequences from related species to identify features conserved at that taxonomic level or higher. Limitations arise from very compact features, limited divergence among available sequences and features subject to relatively weak purifying selection, including newly evolving features perhaps present in only some strains of a virus species (101). Future work will focus on extending the analyses to retroviruses and RNA virus species without designated NCBI RefSeqs, and developing the user interface to the database to allow blast querying and on-the-fly sequence selection and alignment generation for user query sequences.

We predict that synplot2 and the on-line database will be a valuable resource for the virology community. The identification of previously overlooked functional elements will advance fundamental molecular virological research. It also has the potential to resolve previous enigmatic results—e.g. where disruption of undetected overlapping features has confounded mutational analyses of the genes they overlap. By providing a reference map of functional elements embedded within virus protein-coding sequences, synplot2 is relevant to the strategy of using large-scale codon-deoptimization to create attenuated live virus vaccines which cannot easily revert to wild type (102), since this strategy can only work if essential replicational elements are excluded from the codon-deoptimization. Similarly, knowing and understanding all of the essential functional elements is important for the rational design of virus-based gene expression vectors (e.g. for cancer therapy and vaccination).

## AVAILABILITY

The database is available on-line at http://www.firthlab.path.cam.ac.uk/virad.html. The synplot2 source code and a webserver interface are available from the same site and the source code is also included as Supplementary File S1.

### SUPPLEMENTARY DATA

Supplementary Data are available at NAR Online.

## Supplementary Material

SUPPLEMENTARY DATA
