# Supplementary material for: Mapping overlapping functional elements embedded within the protein-coding regions of RNA viruses
Source: Nucleic Acids Res. 2014 Oct 17;42(20):12425–39. doi: 10.1093/nar/gku981 (PMC4227794; doi:10.1093/nar/gku981)
Supplement: SUPPLEMENTARY DATA [file supp_42_20_12425__index.html]

Mapping overlapping functional elements embedded within the protein-coding regions of RNA viruses — Mapping overlapping functional elements embedded within the protein-coding regions of RNA viruses — SUPPLEMENTARY DATA 

# Mapping overlapping functional elements embedded within the protein-coding regions of RNA viruses

## SUPPLEMENTARY DATA

**Files in this Data Supplement:**

- SUPPLEMENTARY DATA
- SUPPLEMENTARY DATA
