# Supplementary material for: Mapping overlapping functional elements embedded within the protein-coding regions of RNA viruses
Source: Nucleic Acids Res. 2014 Oct 17;42(20):12425–39. doi: 10.1093/nar/gku981 (PMC4227794; doi:10.1093/nar/gku981)
Supplement: SUPPLEMENTARY DATA [file supp_gku981_nar-02302-z-2014-File010.pdf]

Mapping overlapping functional elements embedded within the protein-coding regions of RNA viruses

Supplementary Data

Table S1 - List of reference sequences and alignment diversity statistics 2

Synplot2 results for representative RNA viruses (Figures S1 to S16) 5

Table S2 - Additional ORFs added to virus genome maps for the synplot2 analysis 22

Dataset S1 - Regions of reduced synonymous site variability in RNA viruses 28

Table S1. List of reference sequences and alignment diversity statistics for species used in the tables and figures

Main text figures

RefSeq	Species	N_{seqs}[1]	aln. div.[2]	$p_{25,0.7}$[3]	$\frac{\mathrm{obs}}{\mathrm{exp}}\vert_{p=10^{-6}}$[4]
NC_002058.3	Enterovirus C	198	7.23	8.0×10^{-16}	0.82
NC_001449.1	Venezuelan equine encephalitis virus	123	1.54	4.4×10^{-4}	0.57
NC_001961.1	Porcine reproductive and respiratory syndrome virus	194	2.33	3.9×10^{-4}	0.58
NC_002509.2	Turnip mosaic virus	196	4.44	2.3×10^{-8}	0.74
NC_002023.1	Influenza A virus segment 1	198	4.00	1.1×10^{-8}	0.74
NC_002021.1	Influenza A virus segment 2	195	3.70	2.8×10^{-8}	0.74
NC_002022.1	Influenza A virus segment 3	194	2.79	4.3×10^{-6}	0.68
NC_002017.1	Influenza A virus segment 4	192	1.71	1.4×10^{-3}	0.52
NC_002019.1	Influenza A virus segment 5	184	2.73	4.3×10^{-6}	0.68
NC_002018.1	Influenza A virus segment 6	178	2.39	4.5×10^{-4}	0.57
NC_002016.1	Influenza A virus segment 7	179	2.23	7.5×10^{-5}	0.62
NC_002020.1	Influenza A virus segment 8	176	2.57	1.0×10^{-4}	0.62
NC_001434.1	Hepatitis E virus	192	6.85	2.1×10^{-13}	0.80

Tables 1-2 and supplementary figures

RefSeq	Species	N_{seqs}[1]	aln. div.[2]	$p_{25,0.7}$[3]	$\frac{\mathrm{obs}}{\mathrm{exp}}\vert_{p=10^{-6}}$[4]
NC_001943.1	Human astrovirus	31	1.28	2.8×10^{-3}	0.48
NC_011400.1	Astrovirus MLB1	9	0.46	4.1×10^{-2}	0.18
NC_001434.1	Hepatitis E virus	192	6.85	2.1×10^{-13}	0.80
GU345042.1	Rat hepatitis E virus	10	0.70	7.7×10^{-3}	0.41
NC_001961.1	Porcine respiratory and reproductive syndrome virus	194	2.33	3.9×10^{-4}	0.58
NC_002532.2	Equine arteritis virus	27	0.31	6.2×10^{-2}	0.07
NC_001449.1	Venezuelan equine encephalitis virus	123	1.54	4.4×10^{-4}	0.57
NC_003899.1	Eastern equine encephalitis virus	112	0.92	4.5×10^{-3}	0.45
NC_001547.1	Sindbis virus	21	0.59	1.7×10^{-2}	0.32
NC_001545.2	Rubella virus	48	0.57	2.9×10^{-2}	0.25
NC_001477.1	Dengue virus 1	200	1.21	3.2×10^{-3}	0.48
NC_001437.1	Japanese encephalitis virus	196	1.96	9.6×10^{-5}	0.62
NC_001710.1	GB virus C	48	2.36	8.3×10^{-6}	0.67
NC_004102.1	Hepatitis C virus genotype 1	198	8.23	2.0×10^{-12}	0.79
NC_002657.1	Classical swine fever virus	79	2.02	1.4×10^{-4}	0.61
NC_001472.1	Human enterovirus B	198	11.26	2.2×10^{-25}	0.86
NC_001489.1	Hepatitis A virus	89	1.33	8.9×10^{-4}	0.54
NC_001612.1	Human enterovirus A	201	3.73	7.8×10^{-8}	0.73
NC_003985.1	Porcine teschovirus 1	44	2.33	1.1×10^{-5}	0.66
NC_008250.2	Duck hepatitis A virus 1	103	1.27	2.4×10^{-3}	0.49
NC_001897.1	Human parechovirus	78	3.02	2.4×10^{-7}	0.72
NC_001490.1	Human rhinovirus 14	61	3.56	4.4×10^{-8}	0.73
NC_004004.1	Foot-and-mouth disease virus	196	4.32	6.4×10^{-8}	0.73
NC_009448.2	Saffold virus	46	2.08	4.0×10^{-5}	0.64
NC_001366.1	Theilovirus	14	0.72	1.2×10^{-2}	0.37
NC_008311.1	Murine norovirus	68	1.62	2.9×10^{-4}	0.58
NC_001481.2	Feline calicivirus	28	1.73	5.5×10^{-5}	0.63
NC_006269.1	Sapovirus - genogroup 1	10	0.61	1.8×10^{-2}	0.32
NC_010624.1	Sapovirus - genogroup 2	10	0.65	1.2×10^{-2}	0.36
NC_004146.1	Flock house virus RNA 1	6	0.31	8.1×10^{-2}	-0.02
NC_004144.1	Flock house virus RNA 2	5	0.35	7.3×10^{-2}	0.02
NC_004750.1	Barley yellow dwarf virus - PAV	76	1.60	2.2×10^{-3}	0.50

RefSeq	Species	$N_{\mathbf{seqs}}$[1]	aln. div.[2]	$p_{25,0.7}$[3]	$\frac{\text{obs}}{\text{exp}}\|_{p=10^{-6}}$[4]
NC_007289.1	Potato virus S	9	0.49	4.0×10^{-2}	0.18
NC_001554.1	Tomato bushy stunt virus	25	1.25	7.8×10^{-3}	0.41
NC_003375.1	Garlic virus A	3	0.33	7.5×10^{-2}	0.01
NC_001575.2	Rice yellow mottle virus	30	0.60	3.2×10^{-2}	0.23
NC_003462.2	Apple stem pitting virus	11	1.18	1.3×10^{-3}	0.53
NC_002692.1	Tomato mosaic virus	90	1.25	2.8×10^{-3}	0.48
NC_020996.1	Cherry rusty mottle associated virus	9	0.86	8.2×10^{-3}	0.41
NC_001409.1	Apple chlorotic leaf spot virus	14	1.17	2.3×10^{-3}	0.50
NC_011620.1	Potato virus X	23	0.60	2.0×10^{-2}	0.30
NC_004067.1	Pepino mosaic virus	46	0.58	2.3×10^{-2}	0.28
NC_003604.2	Grapevine virus A	12	0.99	4.3×10^{-3}	0.46
NC_001749.2	Apple stem grooving virus	16	0.85	9.6×10^{-3}	0.39
NC_007448.1	Grapevine leafroll-associated virus 2	7	0.44	5.0×10^{-2}	0.13
NC_009992.1	Plum bark necrosis and stem pitting-associated virus	6	0.43	5.5×10^{-2}	0.11
NC_002509.2	Turnip mosaic virus	196	4.44	2.3×10^{-8}	0.74
NC_001616.1	Potato virus Y	198	1.56	1.5×10^{-3}	0.52
NC_002634.1	Soybean mosaic virus	104	1.97	2.8×10^{-4}	0.59
NC_001886.1	Wheat streak mosaic virus	16	0.59	2.0×10^{-2}	0.31
NC_014037.1	Sugarcane streak mosaic virus	7	0.27	6.3×10^{-2}	0.07
NC_002034.1	Cucumber mosaic virus RNA 1	113	1.97	2.9×10^{-4}	0.59
NC_002035.1	Cucumber mosaic virus RNA 2	105	1.14	7.8×10^{-3}	0.41
NC_001440.1	Cucumber mosaic virus RNA 3	195	1.94	6.3×10^{-4}	0.56
NC_006057.1	Arabis mosaic virus RNA 1	14	0.92	7.5×10^{-3}	0.41
NC_006056.1	Arabis mosaic virus RNA 2	61	1.97	2.1×10^{-4}	0.59
NC_003003.1	Broad bean wilt virus 2 RNA 1	31	0.94	4.9×10^{-3}	0.45
NC_003004.1	Broad bean wilt virus 2 RNA 2	37	1.28	1.3×10^{-3}	0.53
NC_003745.1	Saccharomyces cerevisiae virus L-A	4	0.37	3.0×10^{-2}	0.24
NC_017084.1	Tianjin totivirus	3	0.31	6.1×10^{-2}	0.08
NC_003824.1	Trichomonas vaginalis virus 1	10	0.74	1.8×10^{-2}	0.32
NC_002063.1	Leishmania RNA virus 1-1	3	0.36	4.1×10^{-2}	0.18
NC_004178.1	Infectious bursal disease virus segment A	114	1.05	7.2×10^{-3}	0.42
NC_004179.1	Infectious bursal disease virus segment B	98	1.00	5.8×10^{-3}	0.43
NC_001915.1	Infectious pancreatic necrosis virus segment A	69	1.08	6.1×10^{-3}	0.43
NC_001916.1	Infectious pancreatic necrosis virus segment B	31	0.60	2.0×10^{-2}	0.31
NC_011507.2	Rotavirus A segment 1	189	2.66	4.2×10^{-6}	0.68
NC_011506.2	Rotavirus A segment 2	179	2.81	2.9×10^{-6}	0.69
NC_011508.2	Rotavirus A segment 3	189	2.89	9.4×10^{-6}	0.67
NC_011510.2	Rotavirus A segment 4	163	5.84	1.9×10^{-10}	0.77
NC_011500.2	Rotavirus A segment 5	11	0.11	2.2×10^{-1}	-0.87
NC_011509.2	Rotavirus A segment 6	183	3.30	1.9×10^{-7}	0.72
NC_011501.2	Rotavirus A segment 7	175	2.94	1.1×10^{-5}	0.67
NC_011502.2	Rotavirus A segment 8	165	2.68	1.4×10^{-5}	0.66
NC_011503.2	Rotavirus A segment 9	194	4.80	1.1×10^{-8}	0.74
NC_011504.2	Rotavirus A segment 10	190	3.75	3.2×10^{-7}	0.71
NC_011505.2	Rotavirus A segment 11	153	2.09	4.2×10^{-4}	0.57
NC_006023.1	Bluetongue virus segment 1	89	1.59	2.7×10^{-4}	0.59
NC_006013.1	Bluetongue virus segment 2	18	0.49	4.2×10^{-2}	0.17
NC_006014.1	Bluetongue virus segment 3	169	4.28	1.6×10^{-9}	0.76
NC_006024.2	Bluetongue virus segment 4	94	1.85	1.7×10^{-4}	0.60
NC_006025.1	Bluetongue virus segment 5	122	1.93	1.2×10^{-4}	0.61
NC_006010.1	Bluetongue virus segment 6	153	4.42	9.3×10^{-10}	0.76
NC_006022.1	Bluetongue virus segment 7	154	4.81	4.9×10^{-11}	0.78
NC_006007.1	Bluetongue virus segment 8	105	1.78	4.3×10^{-4}	0.57
NC_006008.1	Bluetongue virus segment 9	107	1.14	1.8×10^{-2}	0.32
NC_006015.1	Bluetongue virus segment 10	161	3.32	4.5×10^{-7}	0.71
NC_004282.4	Mammalian orthoreovirus 3 segment L1	22	0.70	6.9×10^{-3}	0.42
NC_004275.1	Mammalian orthoreovirus 3 segment L2	26	1.07	1.2×10^{-3}	0.53

RefSeq	Species	N_{seqs} [1]	aln. div. [2]	$p_{25,0.7}$ [3]	$\frac{obs}{exp}\|_{p=10^{-6}}$ [4]
NC_004274.1	Mammalian orthoreovirus 3 segment L3	20	0.66	6.2×10^{-3}	0.43
NC_004280.1	Mammalian orthoreovirus 3 segment M1	30	0.95	5.7×10^{-3}	0.44
NC_004278.1	Mammalian orthoreovirus 3 segment M2	25	0.78	3.8×10^{-3}	0.47
NC_004281.1	Mammalian orthoreovirus 3 segment M3	20	0.78	7.5×10^{-3}	0.41
NC_004277.1	Mammalian orthoreovirus 3 segment S1	49	0.74	1.6×10^{-2}	0.33
NC_004279.1	Mammalian orthoreovirus 3 segment S2	29	1.25	3.6×10^{-4}	0.58
NC_004283.1	Mammalian orthoreovirus 3 segment S3	39	1.33	5.7×10^{-4}	0.56
NC_004276.1	Mammalian orthoreovirus 3 segment S4	50	1.37	3.7×10^{-4}	0.58
NC_003468.2	Andes virus segment L	41	3.26	6.0×10^{-8}	0.73
NC_003467.2	Andes virus segment M	34	2.72	6.5×10^{-7}	0.70
NC_003466.1	Andes virus segment S	156	6.29	1.9×10^{-13}	0.80
NC_004108.1	La Crosse virus segment L	57	1.39	3.8×10^{-4}	0.58
NC_004109.1	La Crosse virus segment M	72	1.79	1.7×10^{-4}	0.60
NC_004110.1	La Crosse virus segment S	125	1.91	1.7×10^{-4}	0.60
NC_005301.3	Crimean-Congo hemorrhagic fever virus segment L	40	0.97	3.2×10^{-3}	0.48
NC_005300.2	Crimean-Congo hemorrhagic fever virus segment M	54	1.72	1.3×10^{-3}	0.53
NC_005302.1	Crimean-Congo hemorrhagic fever virus segment S	69	1.28	9.9×10^{-4}	0.54
NC_002549.1	Zaire ebolavirus	30	0.46	4.1×10^{-2}	0.18
NC_001781.1	Human respiratory syncytial virus	186	0.84	1.8×10^{-2}	0.32
NC_001542.1	Rabies virus	182	4.53	7.0×10^{-9}	0.75
NC_001498.1	Measles virus	58	0.63	3.3×10^{-2}	0.22
NC_002200.1	Mumps virus	50	0.49	3.5×10^{-2}	0.21
NC_001796.2	Human parainfluenza virus 3	97	0.79	1.6×10^{-2}	0.33
NC_002617.1	Newcastle disease virus	200	2.91	1.6×10^{-5}	0.66

Footnotes

1. Number of unique sequences in the alignment used for the synplot2 analysis.
2. Alignment divergence: mean number of nucleotide substitutions per site over the phylogenetic tree (calculated with the MLOGD package).
3. Mean synplot2 p-value that would be obtained for a 25-codon window in which the reduction in synonymous site variability relative to the null model expectation is 30% (i.e. obs/exp = 0.7).
4. Mean synonymous site variability, expressed as obs/exp, necessary to achieve a p-value of 10^{-6} in a 25-codon window for the alignment.

Note that these are mean-over-genome statistics and may not correspond precisely to p-value and obs/exp statistics in a specific window. They also depend on sliding window size, so are not directly applicable to the 45-codon and 15-codon windows used in Tables 1 and 2.

Synplot2 results for representative RNA viruses

Notes:

1. See website for plots for other RNA viruses.
2. All plots herein are based on automated alignments ($\geq$75% amino acid identity to a given reference sequence) and use a 25-codon sliding window. Alignments are mapped onto reference sequence coordinates by removing alignment columns that have a gap in the reference sequence.
3. Although plots are labelled with a specific species, some alignments (e.g. for *Japanese encephalitis virus*) also include sequences from related species that have $\geq$75% amino acid identity to the chosen reference sequence.
4. In each plot, the brown line (obs/exp) indicates the relative amount of synonymous site variability as represented by the ratio of the observed number of synonymous substitutions to the expected number, in a 25-codon window. The red line shows the corresponding p-value. Note that p-values cannot be compared directly between plots as larger and more diverse alignments provide more statistical power.
5. The dashed grey line represents a p-value of 0.05 / (coding length / window size) - an approximate Bonferroni-like correction for multiple testing. I.e. for each plot, there is an $\sim$5% probability that one or more regions evolving neutrally at synonymous sites would by chance register a signal above the dashed grey line.
6. Note that, in regions where the alignment contains gaps in many sequences, it is possible for the brown obs/exp line to register an extreme value which nonetheless has a non-significant p-value because it is based on a much smaller number of sequences compared to other parts of the alignment.
7. It is important to consider both obs/exp and the p-value. For very large and diverse alignments, p-values can be highly significant even for slight decreases in obs/exp (e.g. **S14.4** *Rabies virus*). The p-value represents statistical significance while obs/exp indicates the degree of purifying selection.
8. Where coding ORFs overlap, the reading frame of the longest of multiple overlapping ORFs is used for defining synonymous codons in the overlap region.
9. Small breaks in the red and brown lines indicate non-coding regions and also junctions between overlapping coding ORFs where a partial codon (i.e. 1 or 2 nt) has been omitted from the calculations.
10. Due to the sliding window, and concatenating coding regions for the synplot2 analysis, it is possible to obtain a false conservation signal at one side of a non-coding gap if there is high conservation at the other side.
11. ORFs are offset vertically according to their frame with respect to nucleotide 1 of the reference sequence (frames 0, 1, 2 from bottom to top). Red '*'s in the genome map represent stop codons.
12. These plots use a 25-codon sliding window. This provides a reasonable compromise between detecting larger overlapping features such as overlapping genes and smaller overlapping features such as non-coding RNA elements. However, some features (particularly smaller RNA elements) are more prominent in plots using smaller sliding window sizes.
13. A 75% identity threshold was used to facilitate automated construction of reasonably robust full-genome alignments. In many cases, greater power for detecting overlapping features can be achieved if more divergent alignments (e.g. $\geq$65% amino acid identity to the reference sequence) are used. For this purpose, single-coding-ORF alignments are often more robust than full-genome alignments.
14. Plots similar but not identical to a small number of these plots have been published previously: 3′ ORF of **S1.1** in Firth et al. (2010), **S1.3** in Emerson et al. (2013), 5′ ORF of **S1.5** in Fang et al. (2012), 5′ ORF of **S2.1** in Kim et al. (2011), **S3.2** in Firth & Atkins (2009), **S5.1** and **S5.2** in Loughran et al. (2011), **S5.3**, **S5.5** and **S5.6** in Valles et al. (2014), and **S6.5** in Ling et al. (2013). They are recalculated here using new sequence alignments (see Methods) and included as well-sequenced representatives of different virus groups.
15. A selection of regions of reduced synonymous site variability that correspond to known or predicted elements are annotated. Abbreviations used: sgRNA – subgenomic RNA; PRF – programmed ribosomal frameshifting; RT – stop codon readthrough; cre – *cis*-acting RNA or *cis*-acting replication element; ISL – internal stem-loop; CSE – conserved sequence element; DSCE – distal subgenomic control element; OA – origin of assembly. The term 'sgRNA promoter' is used for elements involved in sgRNA production irrespective of the mechanism (internal initiation by the RdRp on the antigenome, premature termination by the RdRp during negative-strand synthesis, etc).

Fig. S1.1 *Human astrovirus* (*Astroviridae*, *Mamastrovirus*)

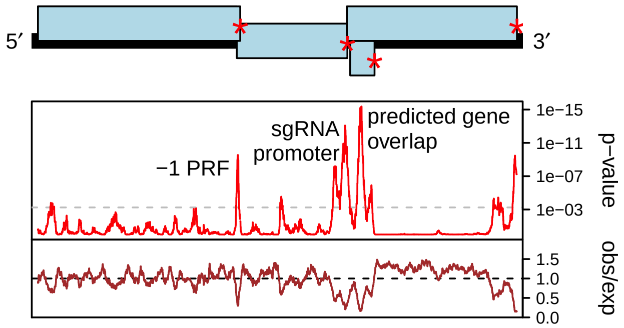

Fig. S1.2 *Astrovirus MLB1* (*Astroviridae*)

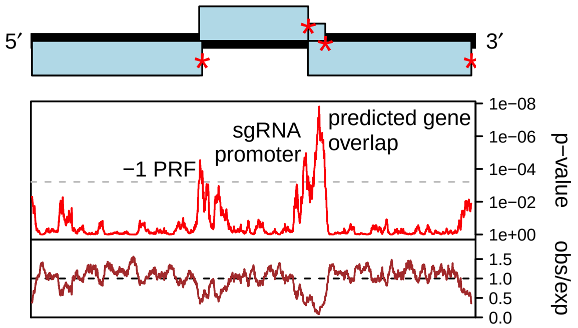

Fig. S1.3 *Hepatitis E virus* (*Hepeviridae*, *Hepevirus*)

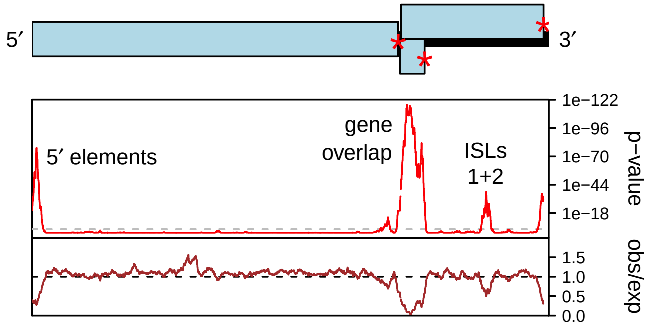

Fig. S1.4 *Rat hepatitis E virus* (*Hepeviridae*, *Hepevirus*)

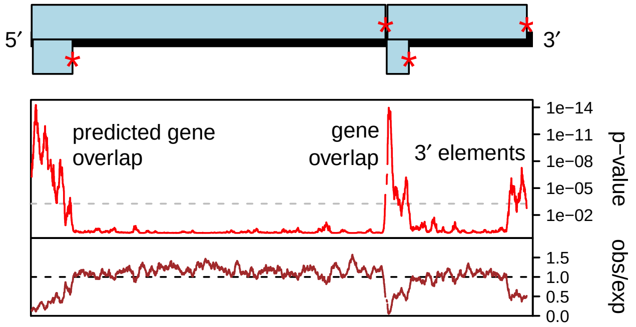

Fig. S1.5 *Porcine respiratory and reproductive syndrome virus* (*Arteriviridae*, *Arterivirus*)

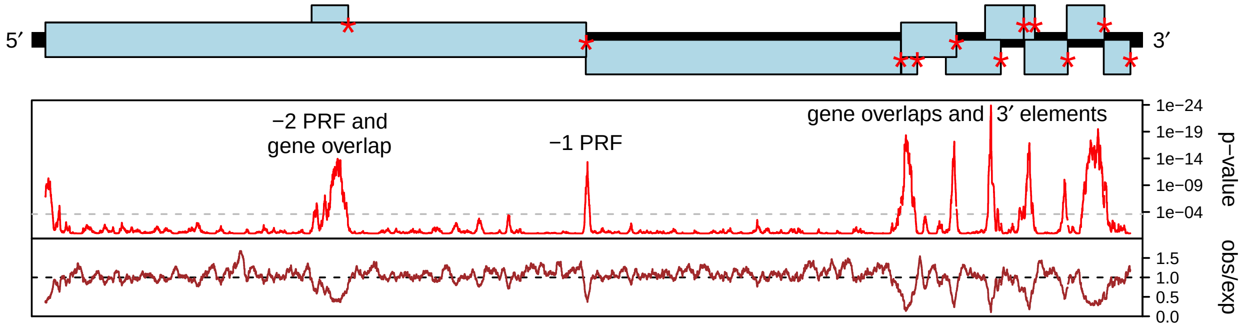

Fig. S1.6 *Equine arteritis virus* (*Arteriviridae*, *Arterivirus*)

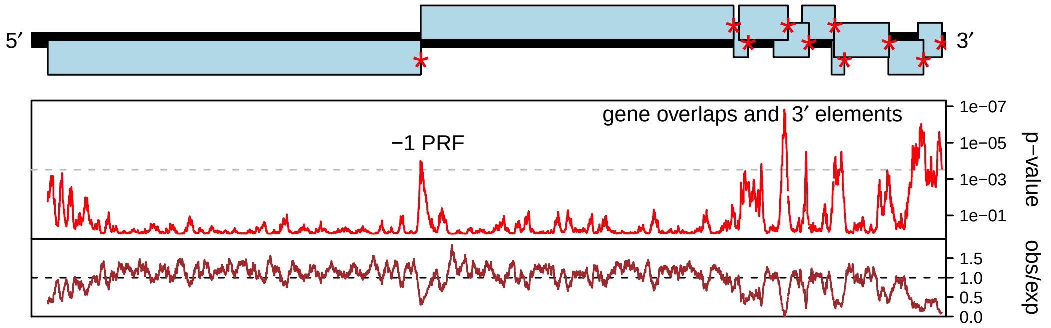

Fig. S2.1 *Venezuelan equine encephalitis virus* (*Togaviridae, Alphavirus*)

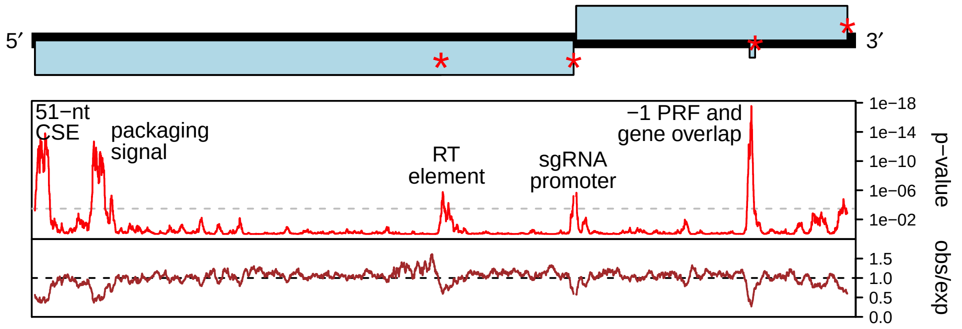

Fig. S2.2 *Eastern equine encephalitis virus* (*Togaviridae, Alphavirus*)

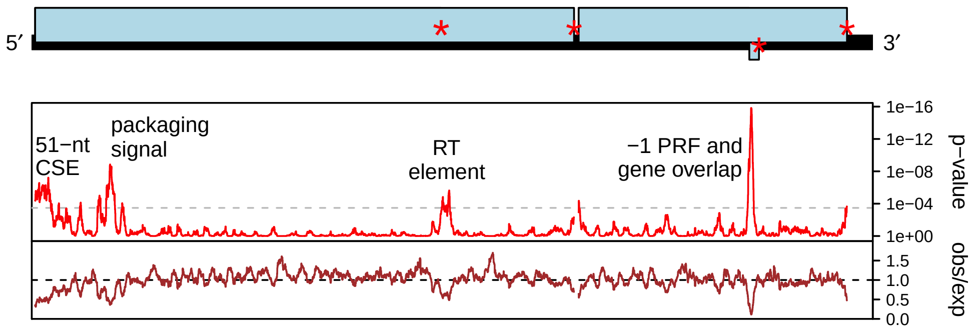

Fig. S2.3 *Sindbis virus* (*Togaviridae, Alphavirus*)

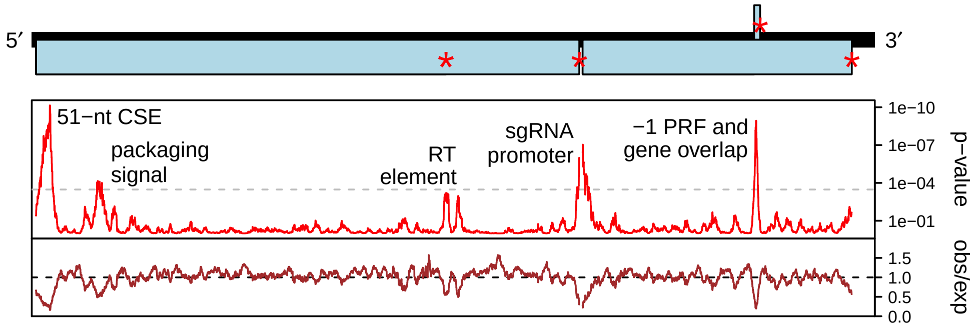

Fig. S2.4 *Rubella virus* (*Togaviridae, Rubivirus*)

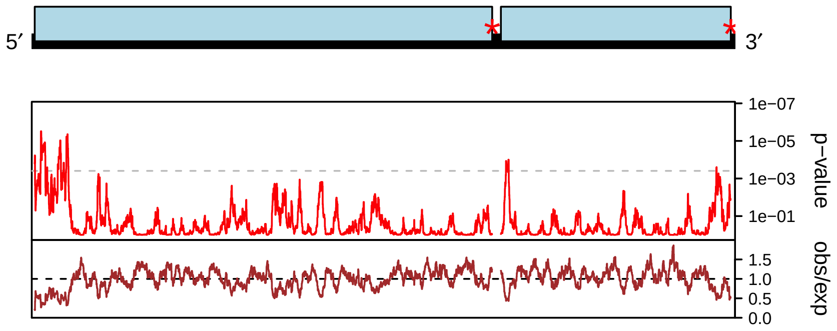

Fig. S3.1 *Dengue virus 1* (*Flaviviridae*, *Flavivirus*)

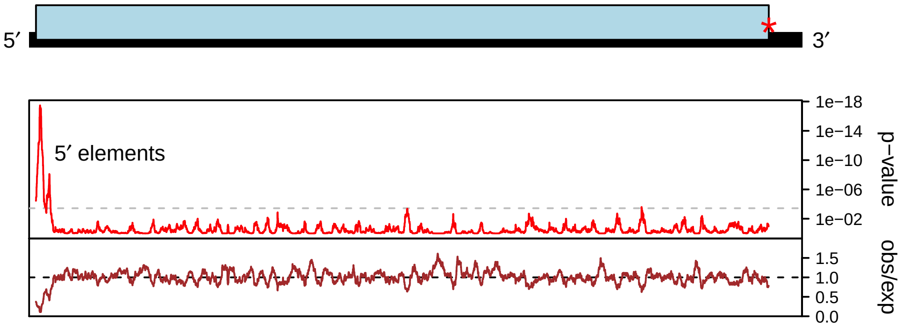

Fig. S3.2 *Japanese encephalitis virus* (*Flaviviridae*, *Flavivirus*)

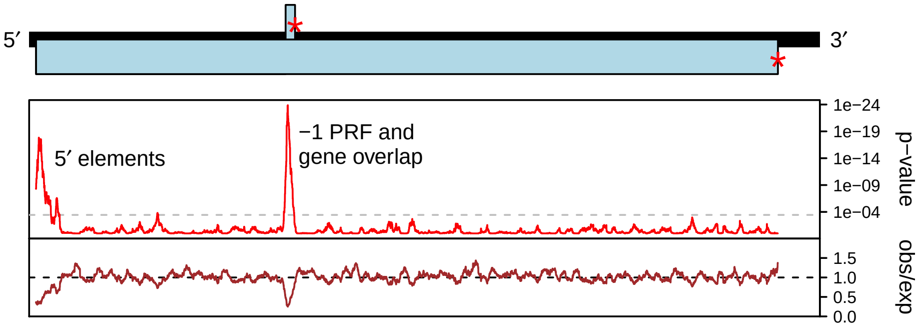

Fig. S3.3 *GB virus C* (*Flaviviridae*, *Pegivirus*)

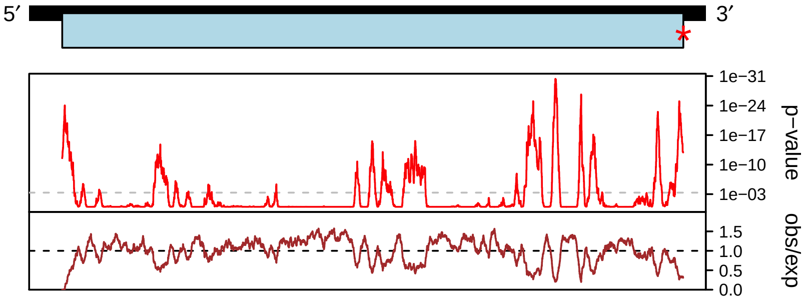

Fig. S3.4 *Hepatitis C virus* (*Flaviviridae*, *Hepacivirus*)

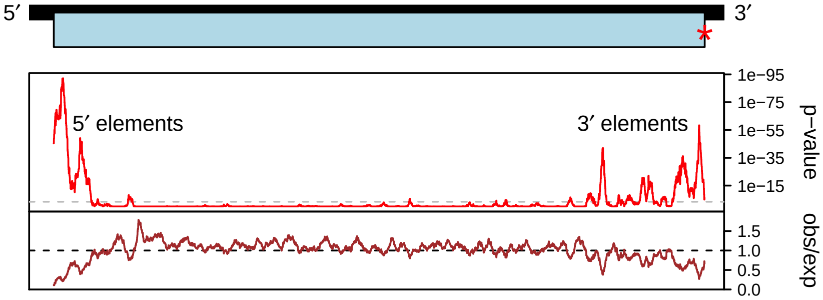

Fig. S3.5 *Classical swine fever virus* (*Flaviviridae*, *Pestivirus*)

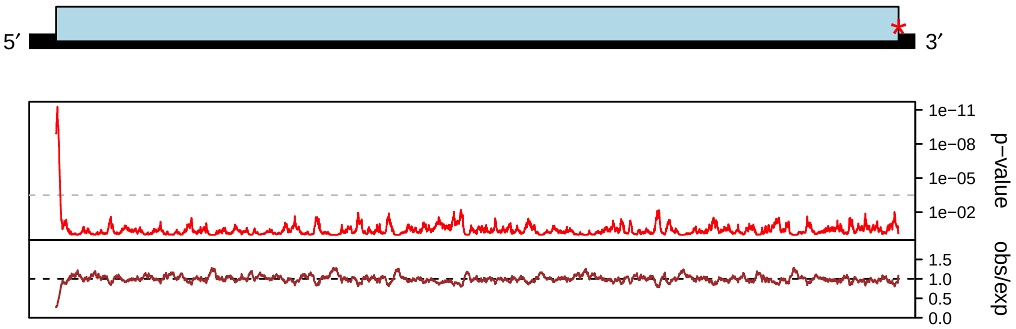

Fig. S4.1 *Human enterovirus B* (*Picornaviridae, Enterovirus*)

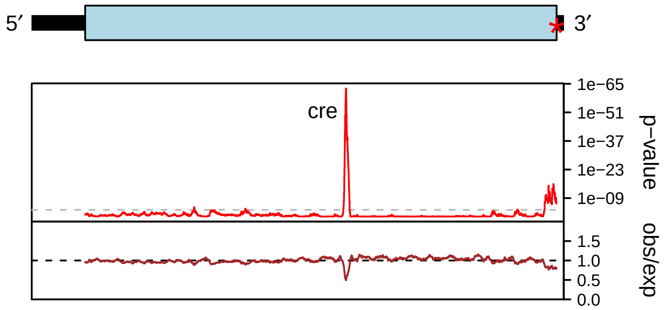

Fig. S4.2 *Hepatitis A virus* (*Picornaviridae, Hepatovirus*)

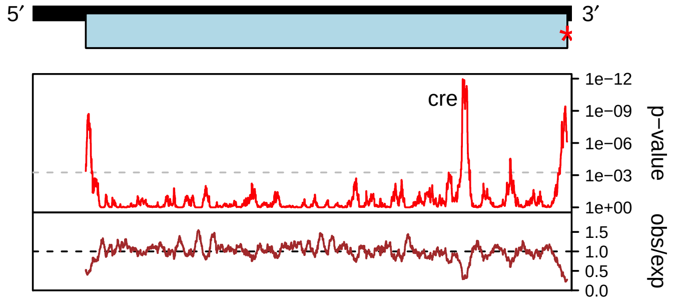

Fig. S4.3 *Human enterovirus A* (*Picornaviridae, Enterovirus*)

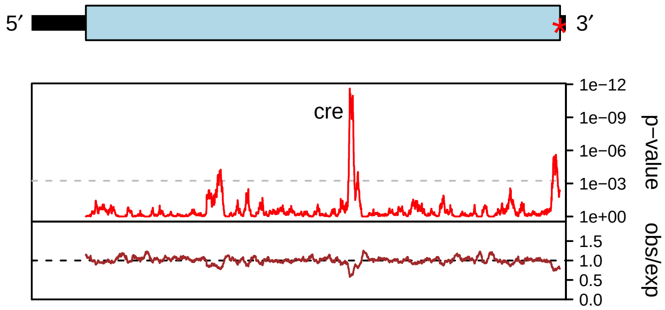

Fig. S4.4 *Porcine teschovirus 1* (*Picornaviridae, Teschovirus*)

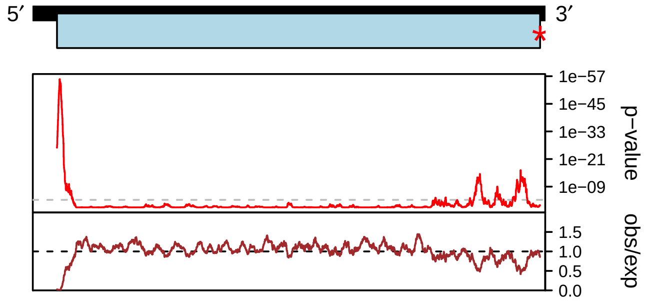

Fig. S4.5 *Duck hepatitis A virus 1* (*Picornaviridae, Avihepatovirus*)

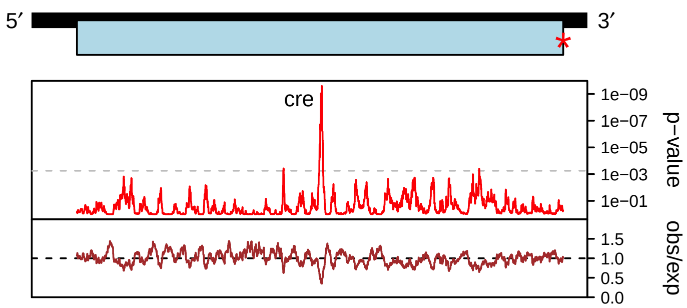

Fig. S4.6 *Human parechovirus* (*Picornaviridae, Parechovirus*)

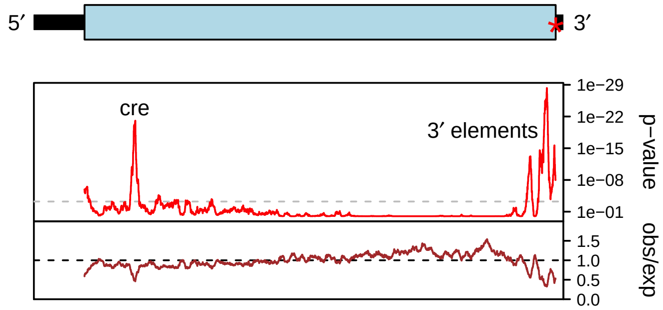

Fig. S4.7 *Human rhinovirus 14* (*Picornaviridae, Enterovirus*)

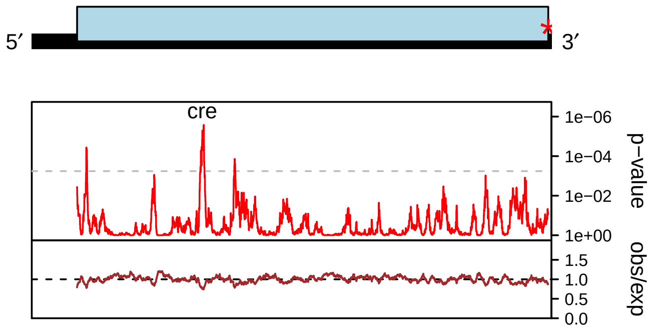

Fig. S4.8 *Foot-and-mouth disease virus* (*Picornaviridae, Aphthovirus*)

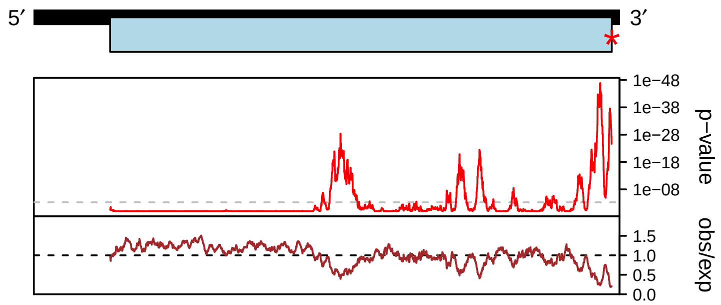

Fig. S5.1 *Saffold virus* (*Picornaviridae, Cardiovirus*)

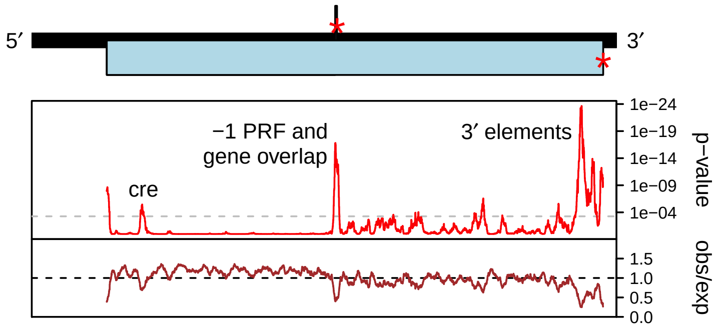

Fig. S5.2 *Theilovirus* (*Picornaviridae, Cardiovirus*)

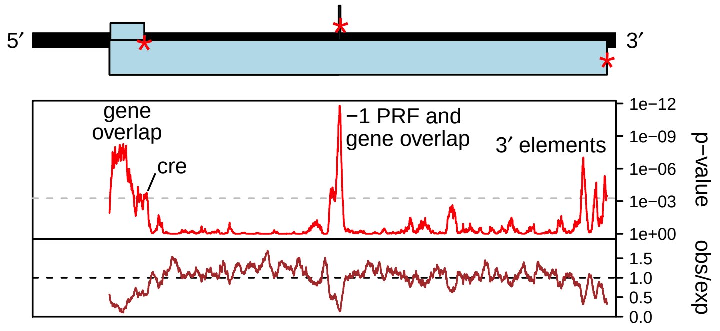

Fig. S5.3 *Murine norovirus 1* (*Caliciviridae, Norovirus*)

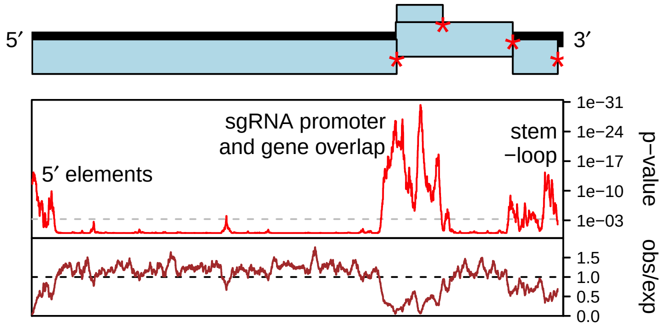

Fig. S5.4 *Feline calicivirus* (*Caliciviridae, Vesivirus*)

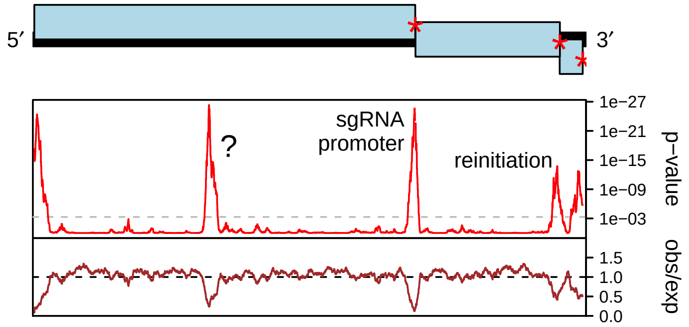

Fig. S5.5 *Sapovirus - genogroup 1* (*Caliciviridae, Sapovirus*)

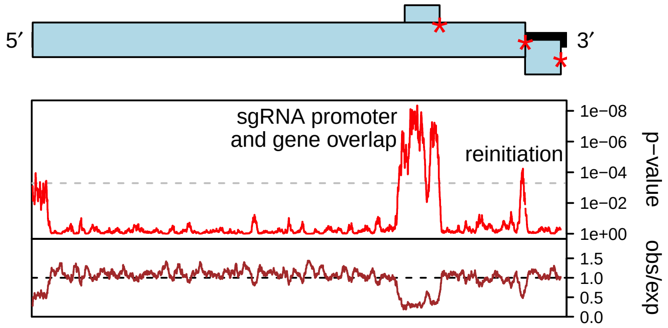

Fig. S5.6 *Sapovirus - genogroup 2* (*Caliciviridae, Sapovirus*)

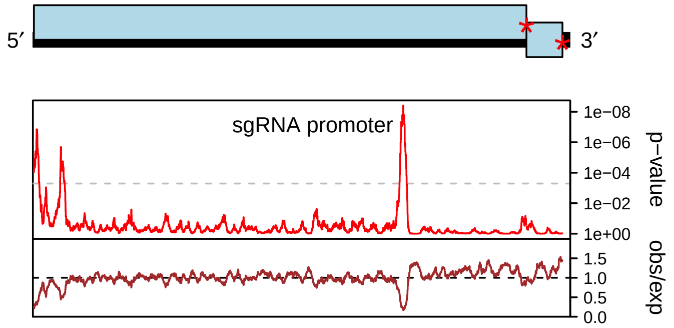

Fig. S5.7 *Flock house virus* (*Nodaviridae, Alphanodavirus*)

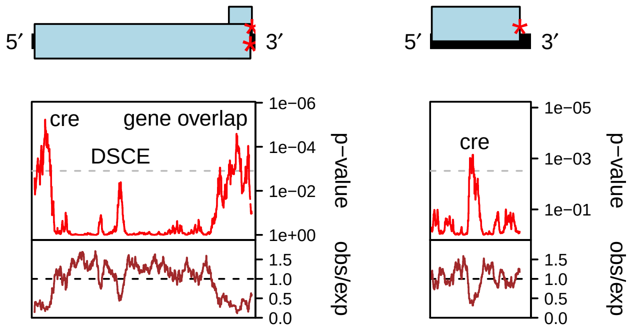

Fig. S5.8 *Striped jack nervous necrosis virus* (*Nodaviridae, Betanodavirus*)

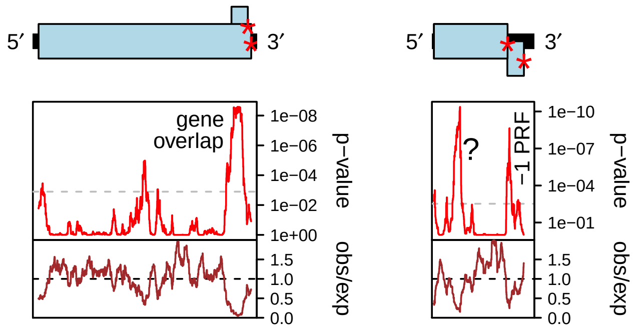

Fig. S6.1 *Barley yellow dwarf virus - PAV* (*Luteoviridae, Luteovirus*)

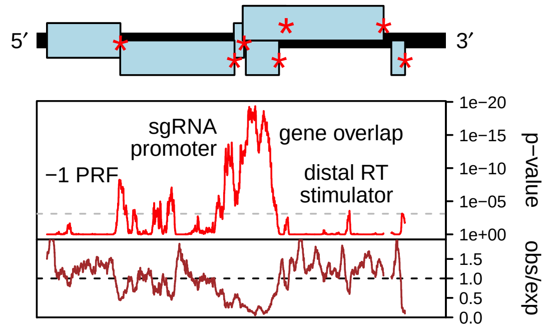

Fig. S6.2 *Potato virus S* (*Betaflexiviridae, Carlavirus*)

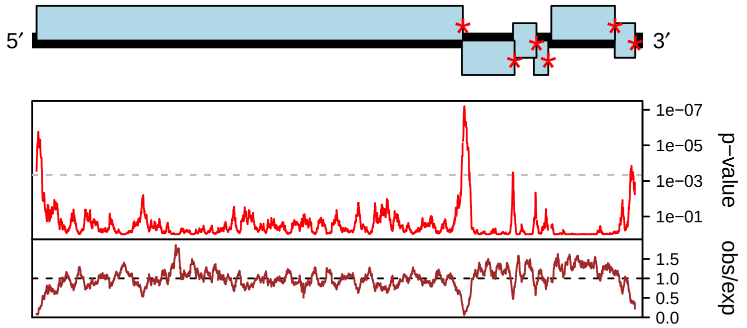

Fig. S6.3 *Tomato bushy stunt virus* (*Tombusviridae, Tombusvirus*)

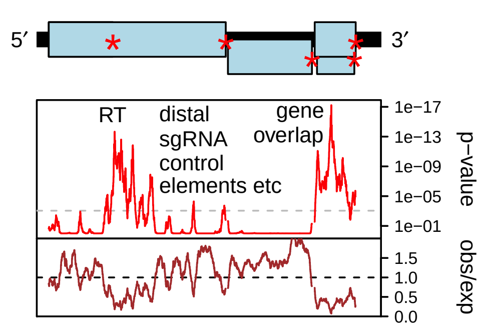

Fig. S6.4 *Garlic virus A* (*Alphaflexiviridae, Allexivirus*)

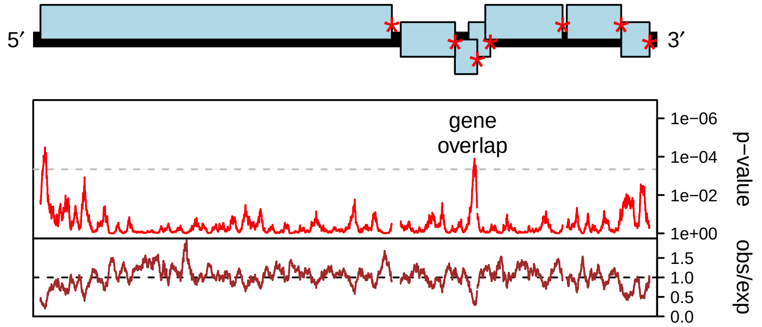

Fig. S6.5 *Rice yellow mottle virus* (*Sobemovirus*)

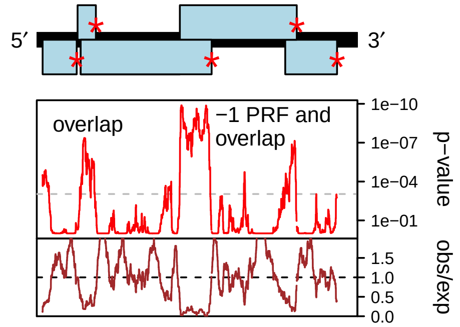

Fig. S6.6 *Apple stem pitting virus* (*Betaflexiviridae, Foveavirus*)

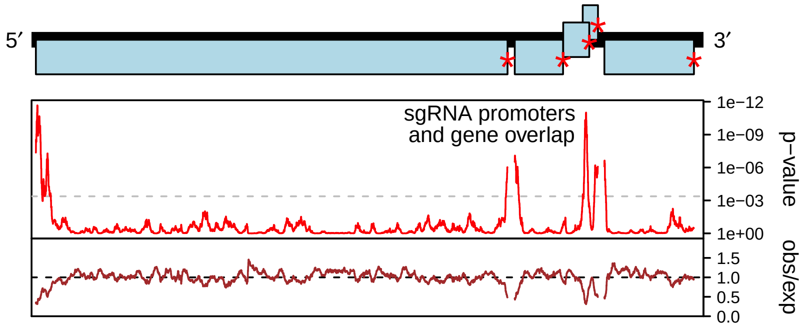

Fig. S6.7 *Tomato mosaic virus* (*Virgaviridae, Tobamovirus*)

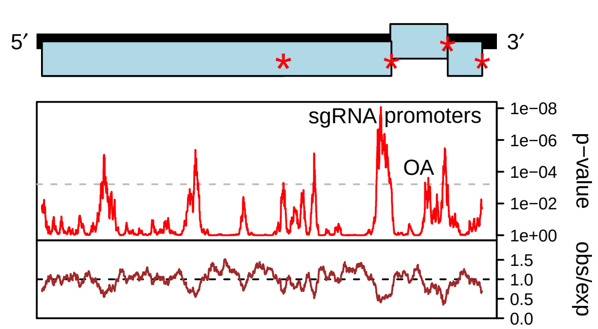

Fig. S6.8 *Cherry rusty mottle associated virus* (*Betaflexiviridae*)

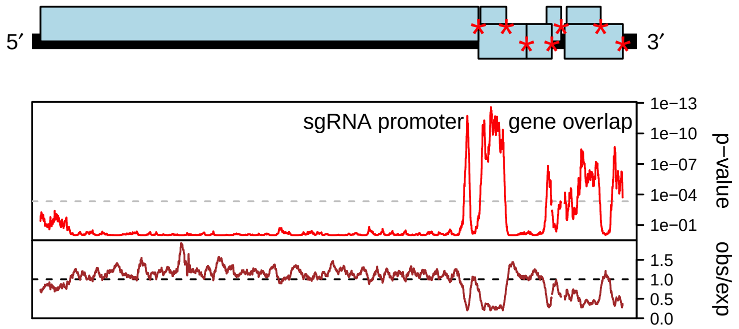

Fig. S7.1 *Apple chlorotic leaf spot virus* (*Betaflexiviridae, Trichovirus*)

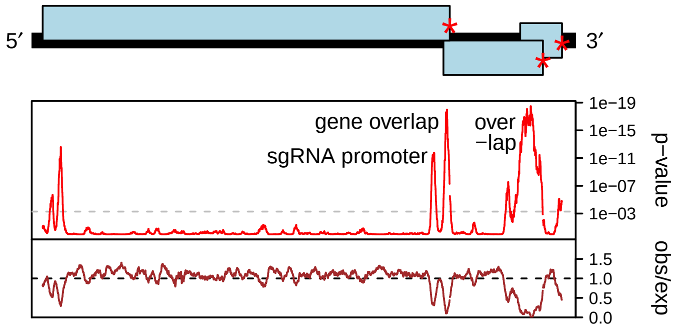

Fig. S7.2 *Potato virus X* (*Alphaflexiviridae, Potexvirus*)

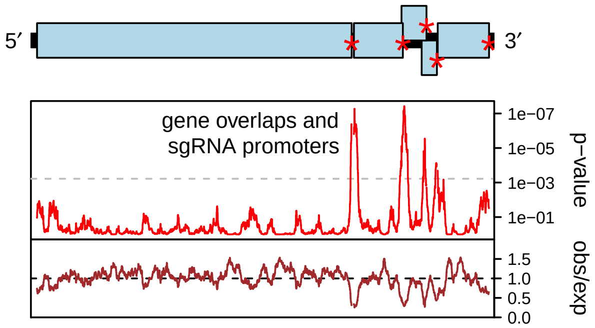

Fig. S7.3 *Grapevine virus A* (*Betaflexiviridae, Vitivirus*)

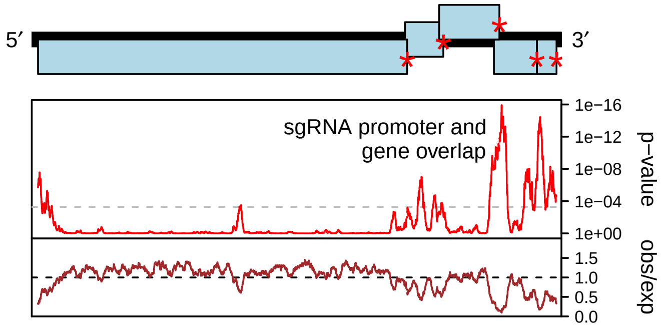

Fig. S7.4 *Apple stem grooving virus* (*Betaflexiviridae, Capillovirus*)

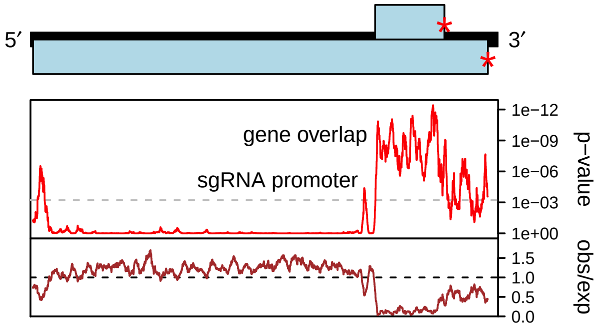

Fig. S7.5 *Grapevine leafroll-associated virus 2* (*Closteroviridae, Closterovirus*)

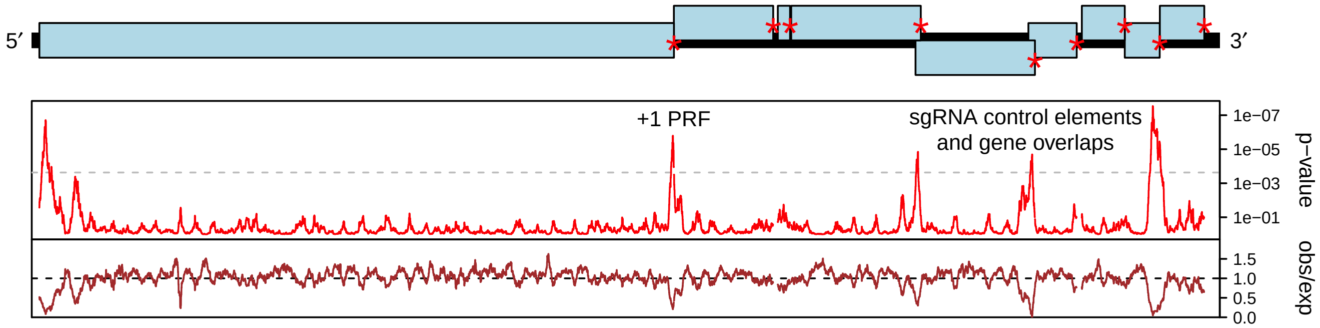

Fig. S7.6 *Plum bark necrosis and stem pitting-associated virus* (*Closteroviridae, Ampelovirus*)

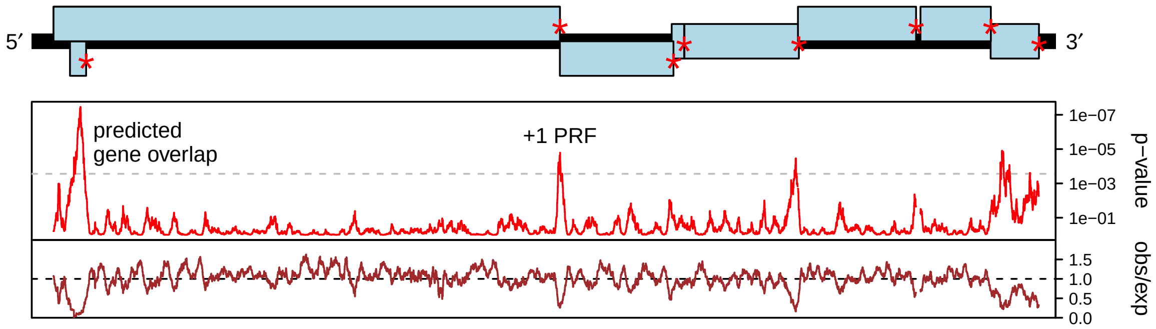

Fig. S8.1 *Turnip mosaic virus* (*Potyviridae, Potyvirus*)

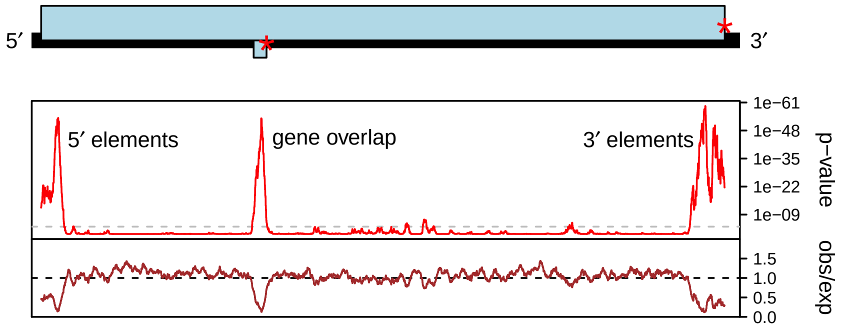

Fig. S8.2 *Potato virus Y* (*Potyviridae, Potyvirus*)

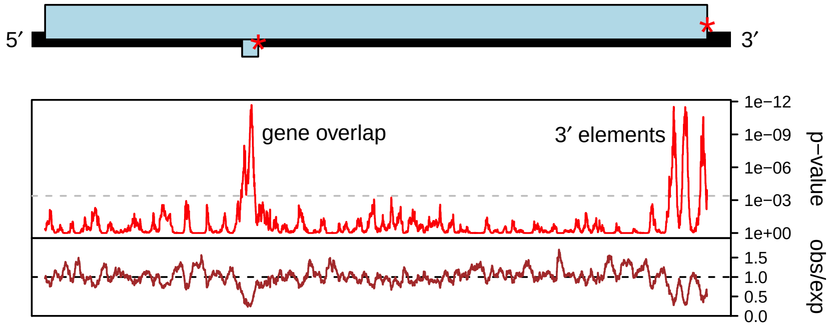

Fig. S8.3 *Soybean mosaic virus* (*Potyviridae, Potyvirus*)

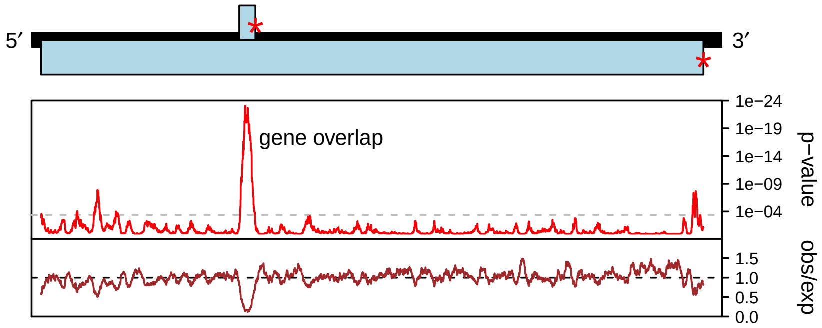

Fig. S8.4 *Wheat streak mosaic virus* (*Potyviridae, Tritimovirus*)

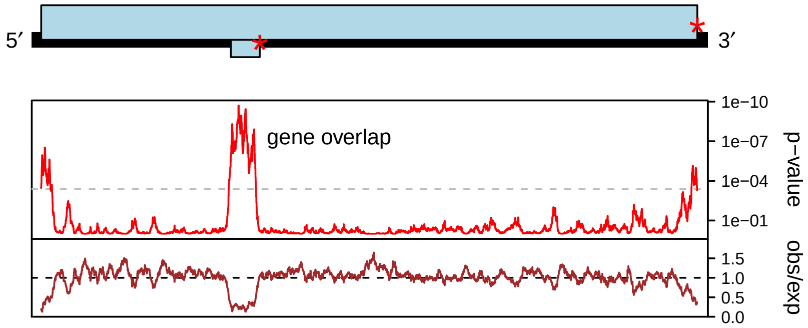

Fig. S9.1 *Sugarcane streak mosaic virus* (*Potyviridae, Poacevirus*)

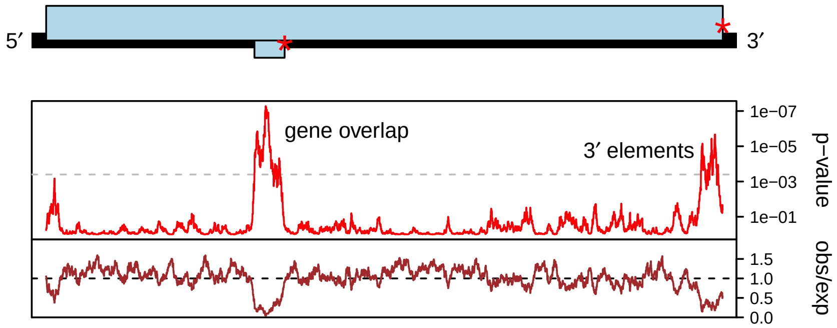

Fig. S9.2 *Cucumber mosaic virus* (*Bromoviridae, Cucumovirus*)

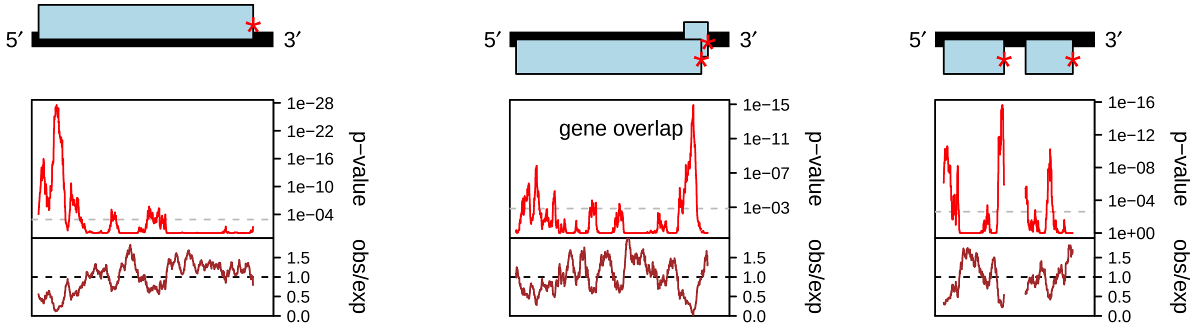

Fig. S9.3 *Arabis mosaic virus* (*Secoviridae, Nepovirus*)

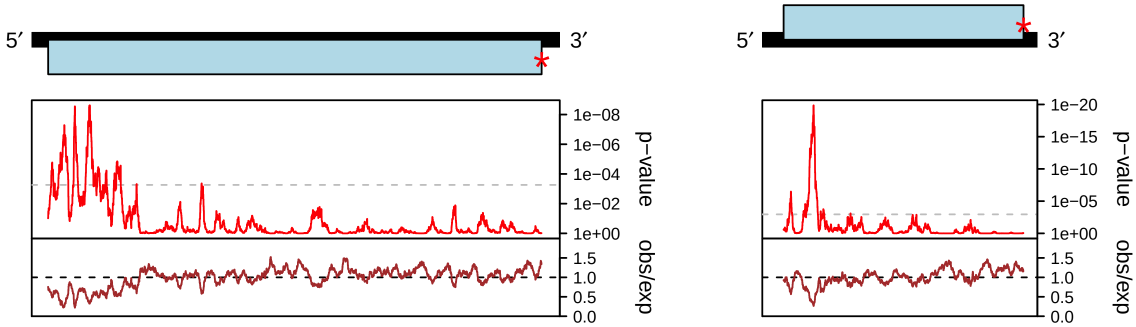

Fig. S9.4 *Broad bean wilt virus 2* (*Secoviridae, Fabavirus*)

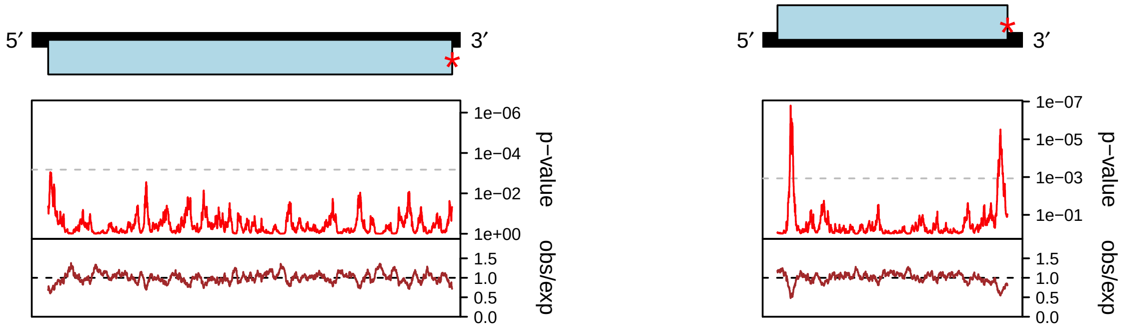

Fig. S10.1 *Saccharomyces cerevisiae virus L-A* (*Totiviridae*, *Totivirus*)

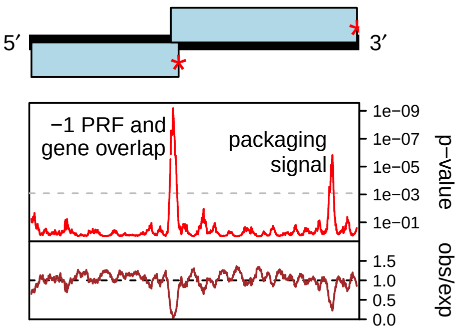

Fig. S10.2 *Tianjin totivirus* (*Totiviridae*)

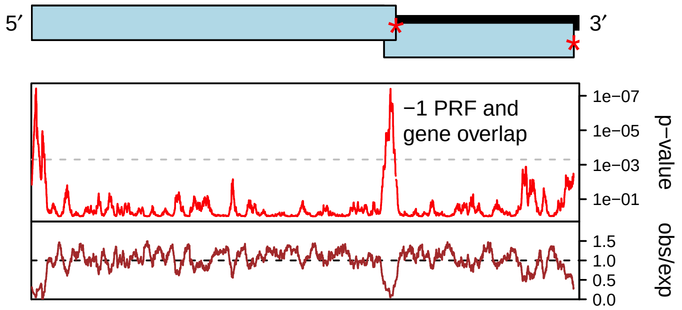

Fig. S10.3 *Trichomonas vaginalis virus 1* (*Totiviridae*, *Trichomonasvirus*)

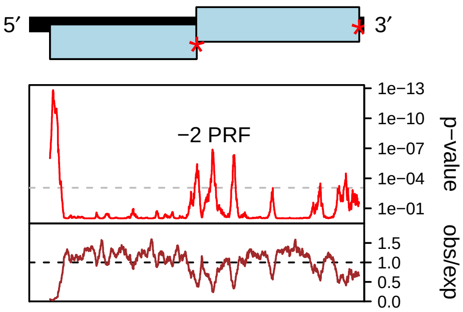

Fig. S10.4 *Leishmania RNA virus 1-1* (*Totiviridae*, *Leishmaniavirus*)

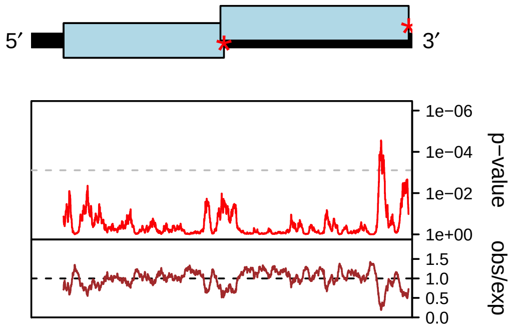

Fig. S10.5 *Infectious bursal disease virus* (*Birnaviridae*, *Avibirnavirus*)

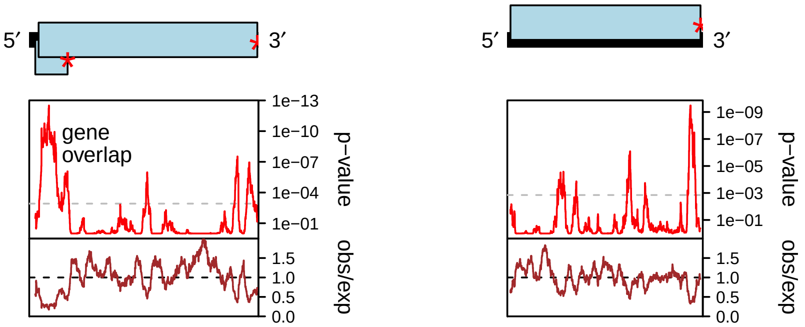

Fig. S10.6 *Infectious pancreatic necrosis virus* (*Birnaviridae*, *Aquabirnavirus*)

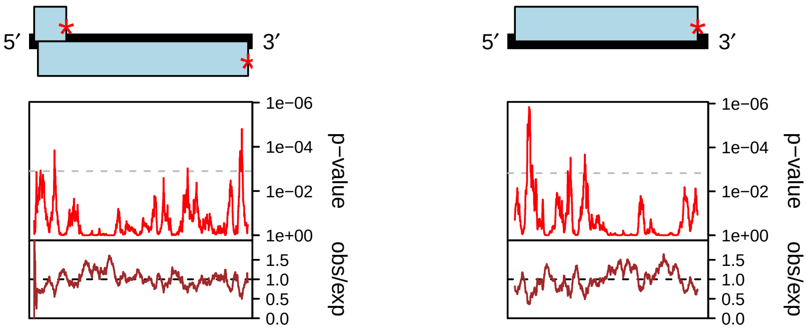

Fig. S11.1 *Rotavirus A* (*Reoviridae, Rotavirus*)

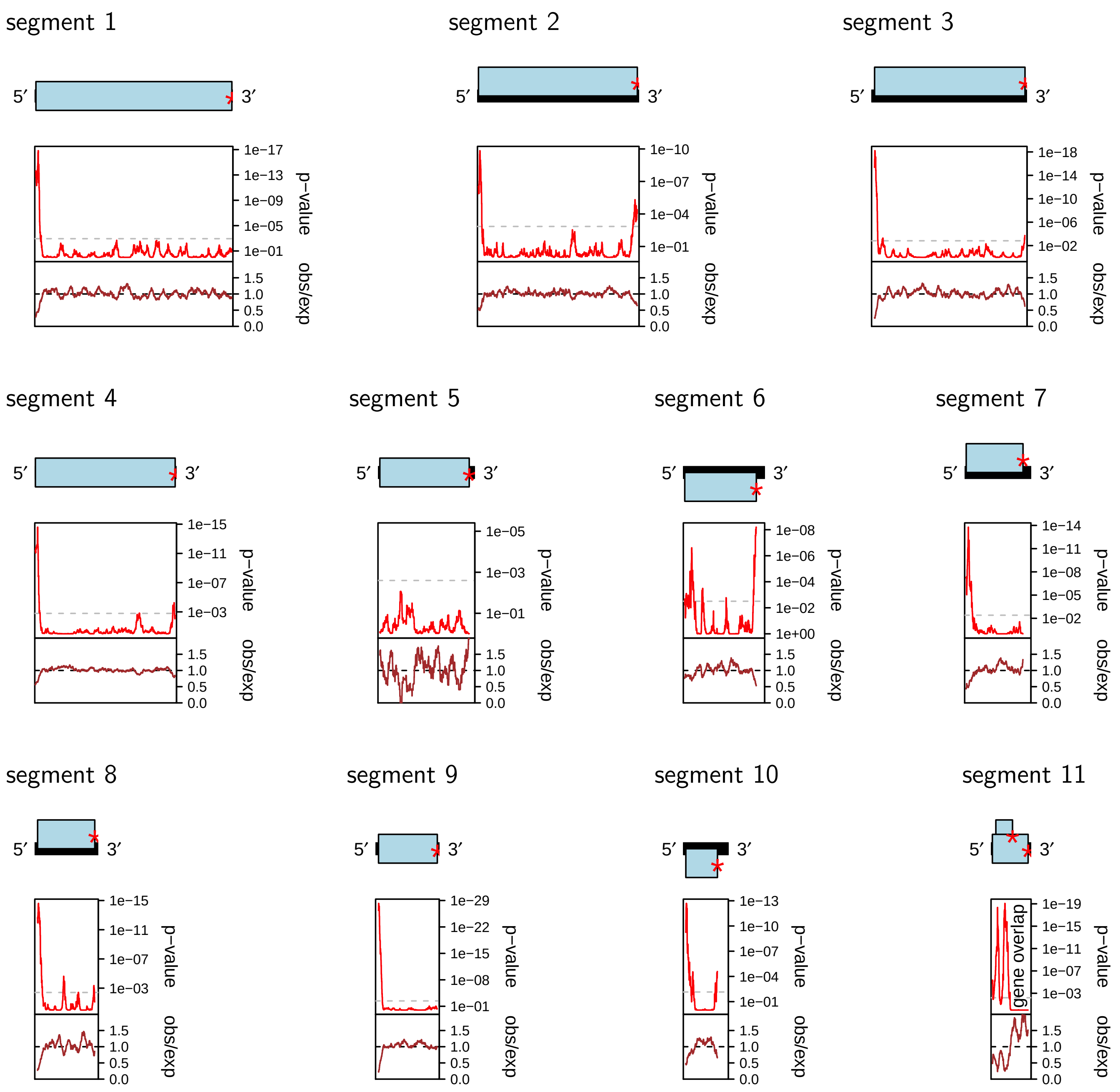

Fig. S12.1 *Bluetongue virus* (*Reoviridae, Orbivirus*)

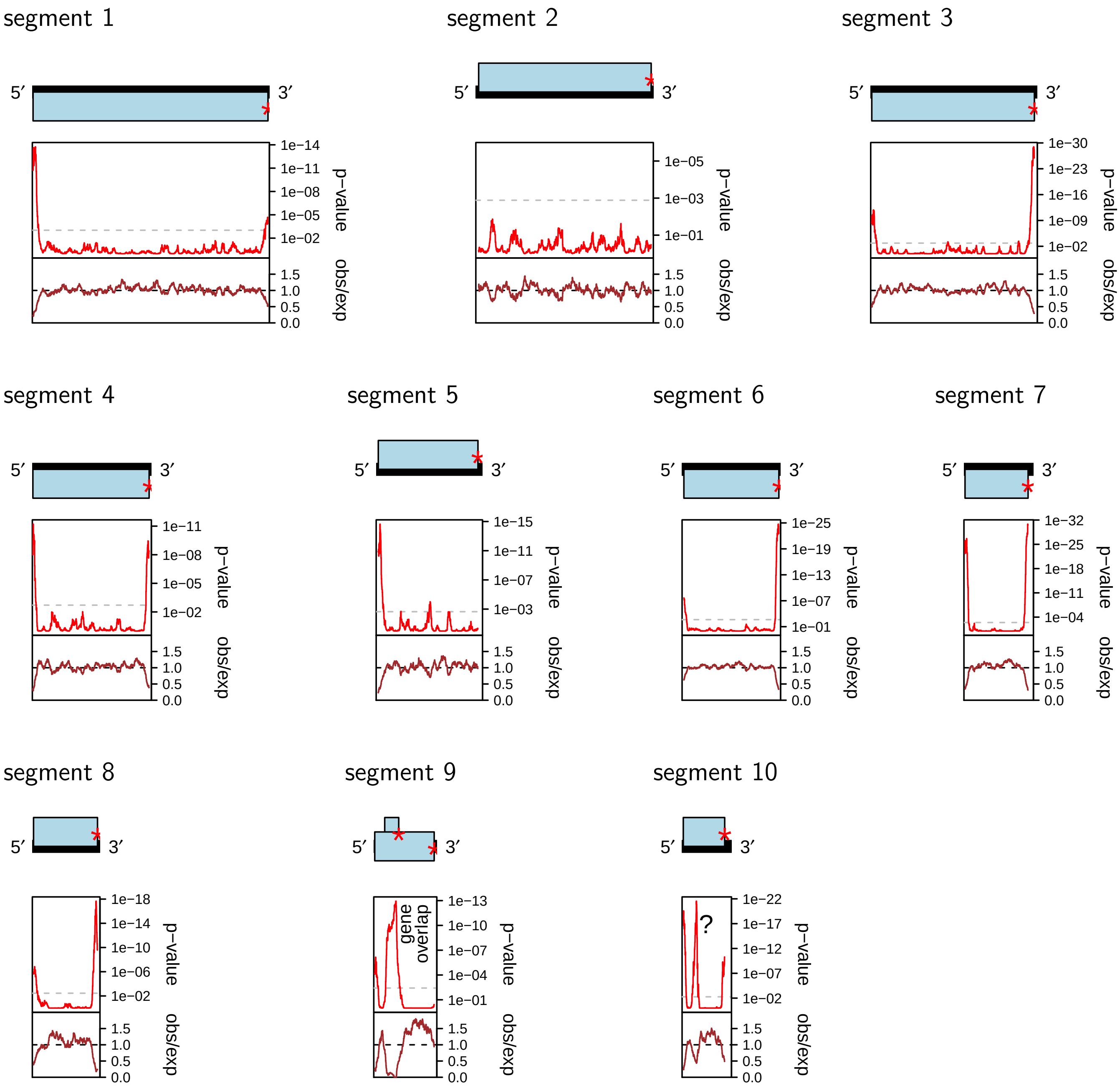

Fig. S13.1 *Mammalian orthoreovirus 3* (*Reoviridae*, *Orthoreovirus*)

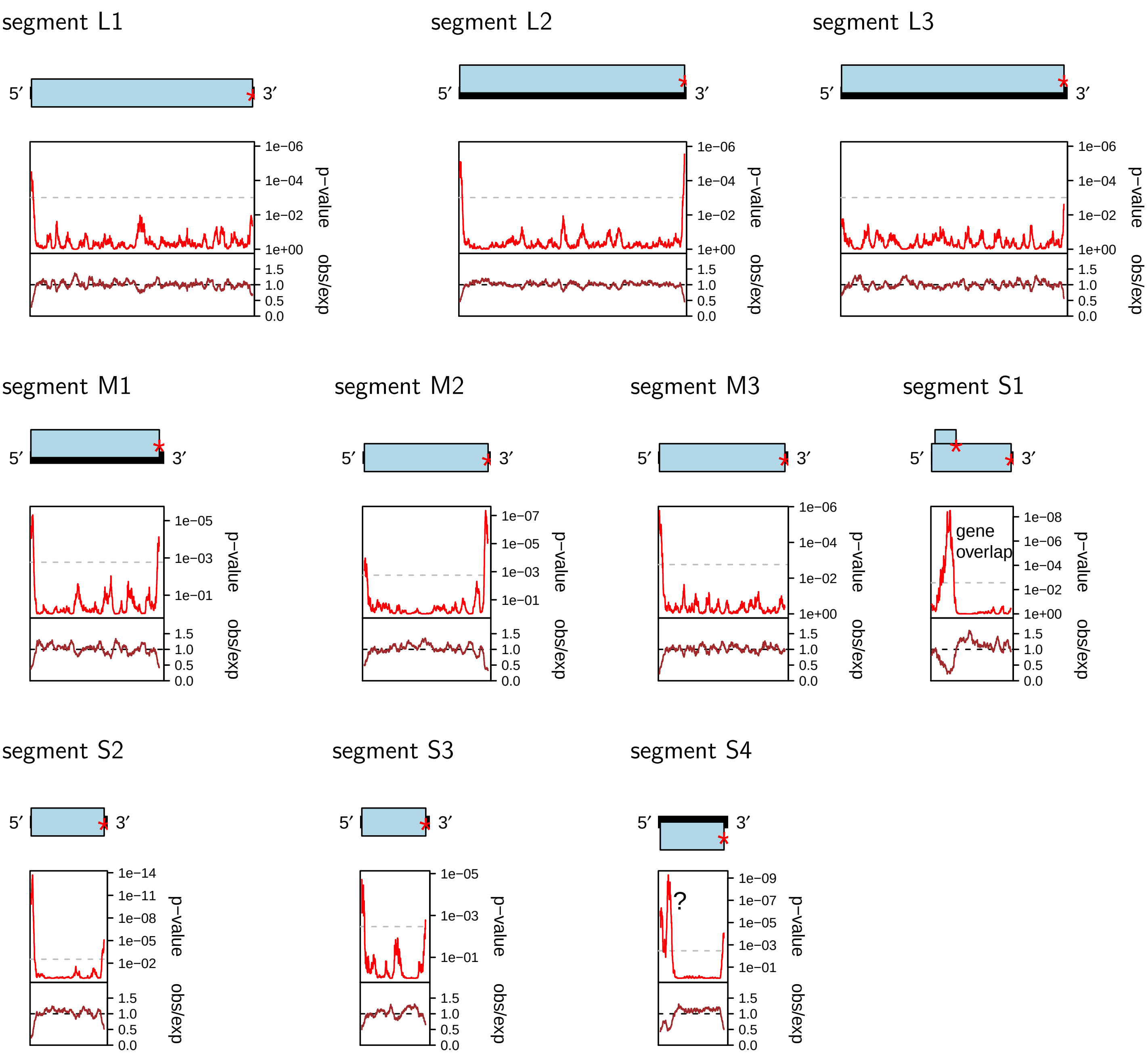

Fig. S14.1 *Andes virus* (*Bunyaviridae, Hantavirus*)

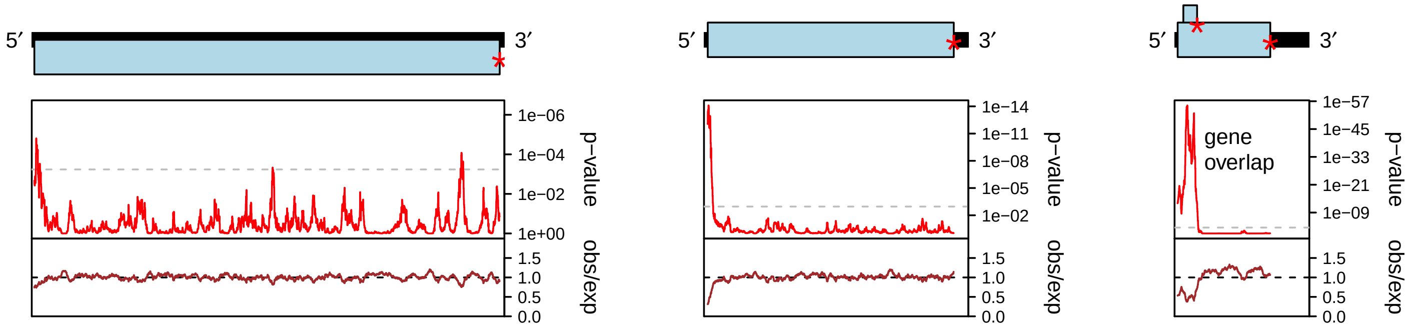

Fig. S14.2 *La Crosse virus* (*Bunyaviridae, Orthobunyavirus*)

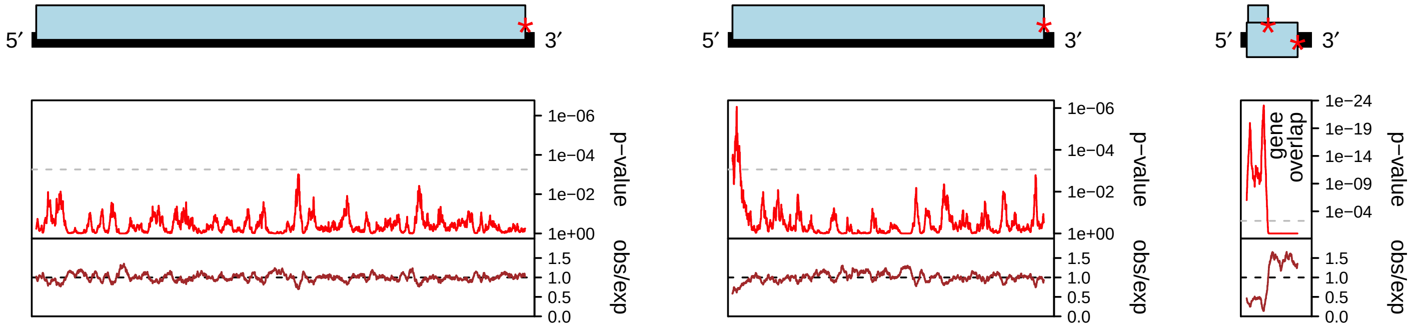

Fig. S14.3 *Crimean-Congo hemorrhagic fever virus* (*Bunyaviridae, Nairovirus*)

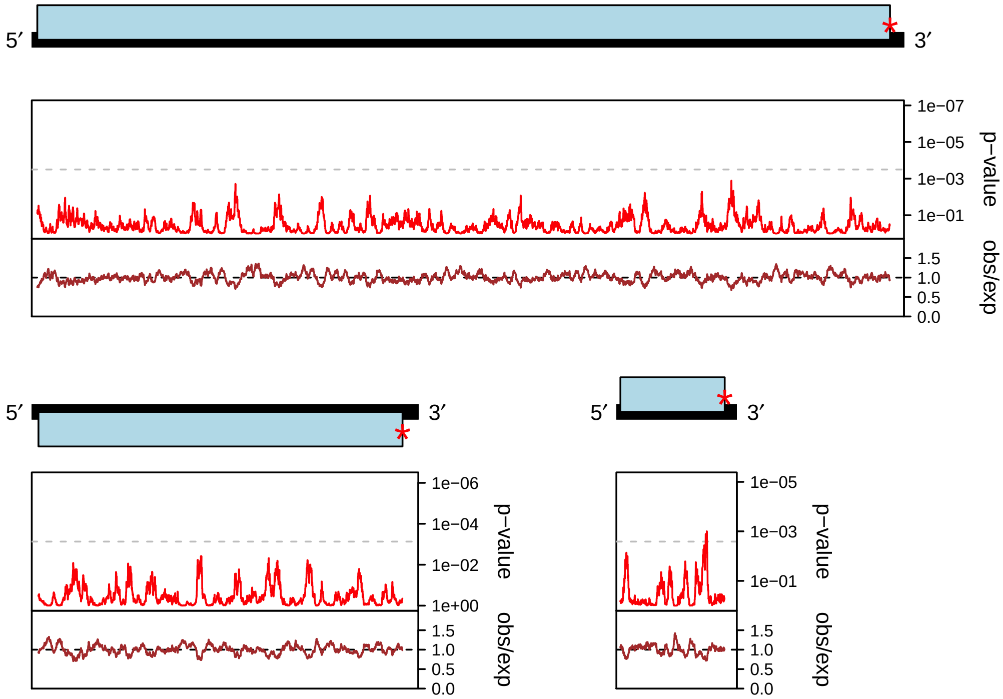

Fig. S15.1 *Zaire ebolavirus* (*Filoviridae, Ebolavirus*)

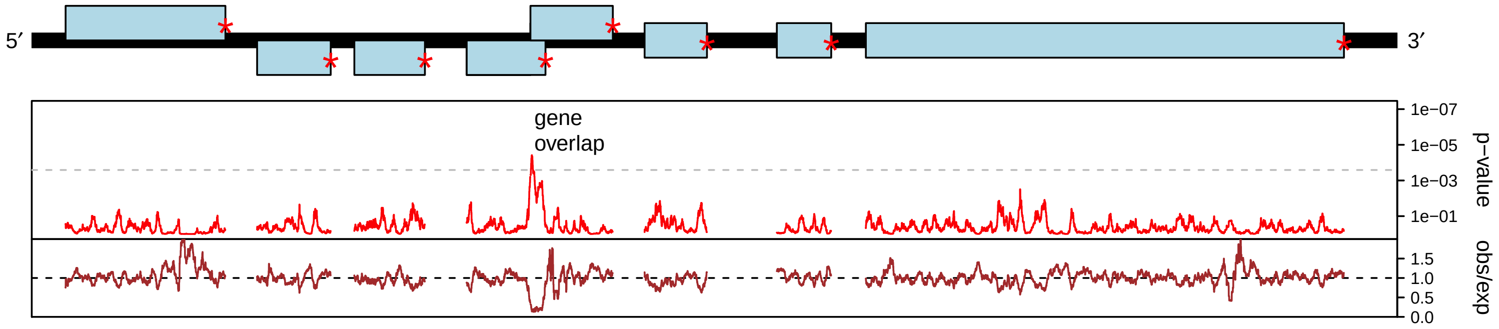

Fig. S15.2 *Human respiratory syncytial virus* (*Paramyxoviridae, Pneumovirus*)

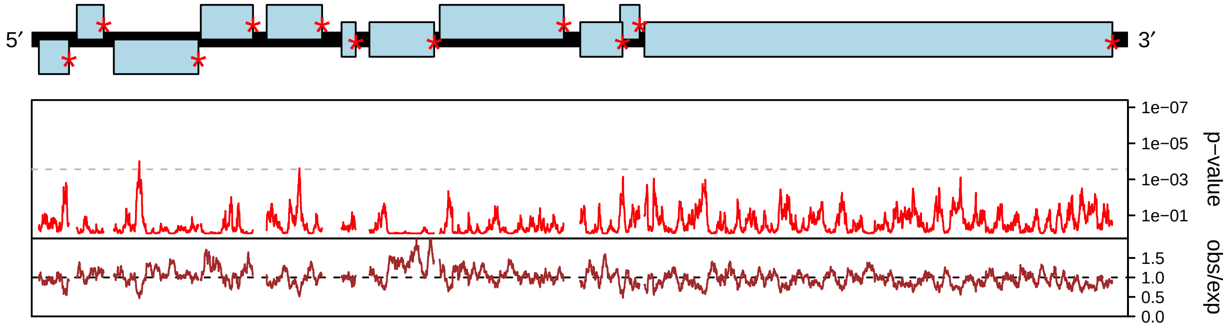

Fig. S15.3 *Rabies virus* (*Rhabdoviridae, Lyssavirus*)

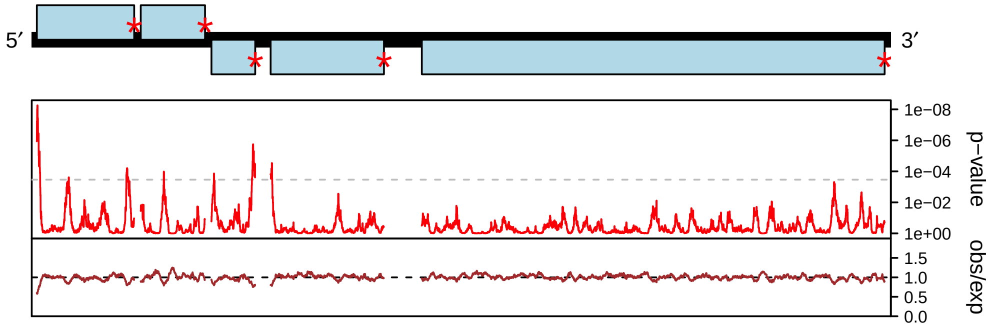

Fig. S16.1 *Measles virus* (*Paramyxoviridae, Morbillivirus*)

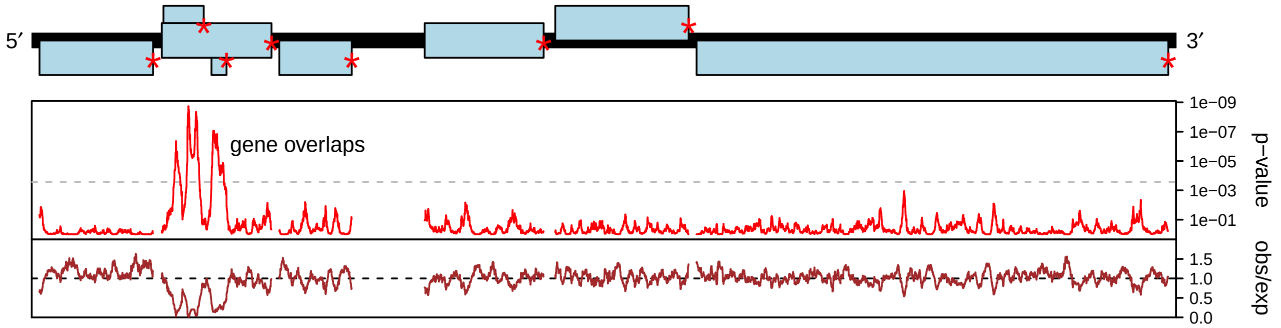

Fig. S16.2 *Mumps virus* (*Paramyxoviridae, Rubulavirus*)

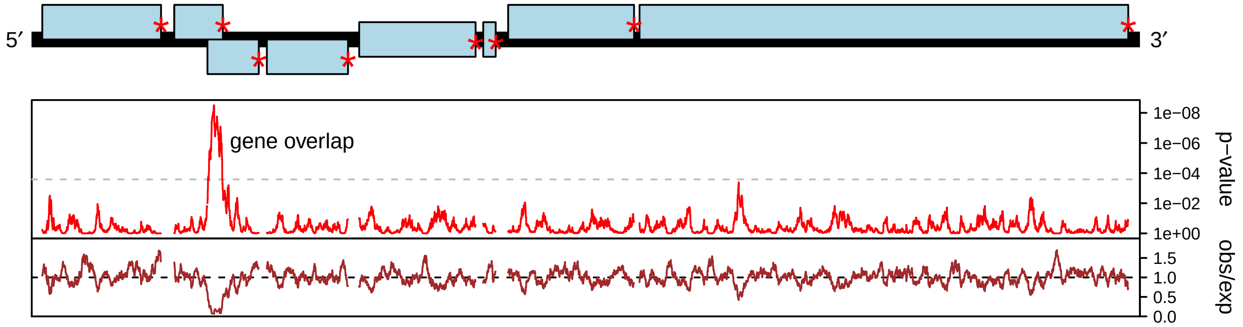

Fig. S16.3 *Human parainfluenza virus 3* (*Paramyxoviridae, Respirovirus*)

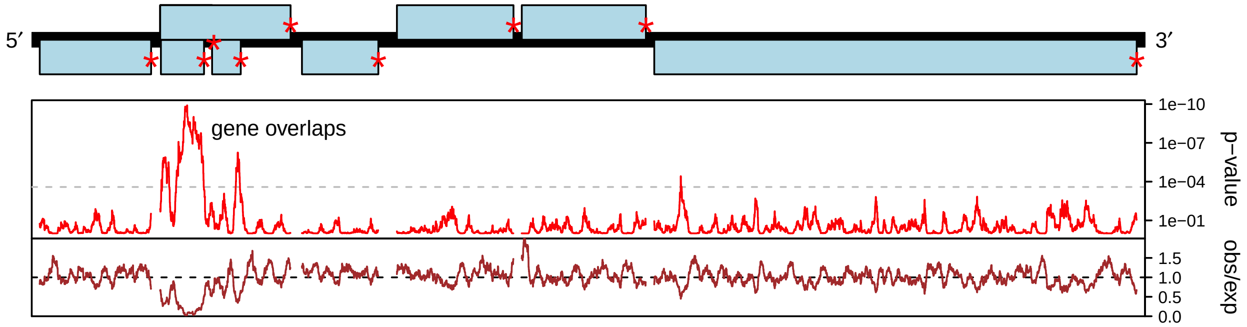

Fig. S16.4 *Newcastle disease virus* (*Paramyxoviridae, Avulavirus*)

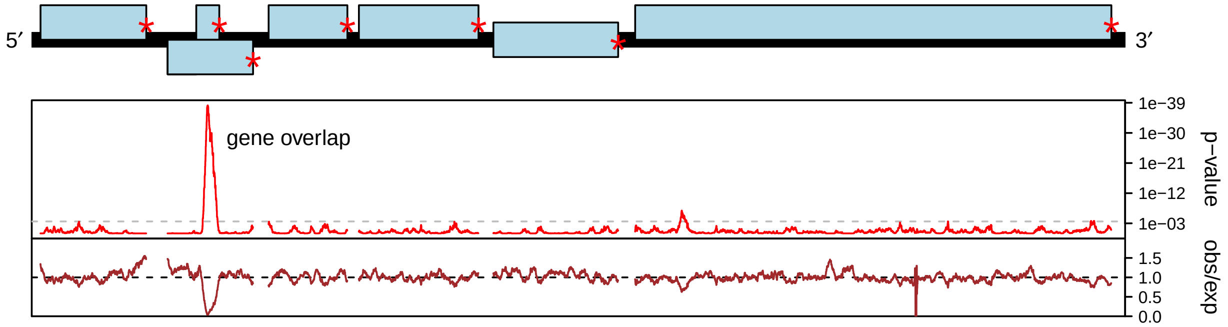

Table S2. Additional ORFs added to virus genome maps for synplot2 analysis

Single-stranded positive-sense RNA viruses

Taxon	Example sequence	Nucleotide coordinates	Product	Translation mechanism[1]	Status	References
Acyrthosiphon pisum virus, *Rosy apple aphid virus*	NC_003780.1	6716..6949	-	Leaky scanning	-	Valles 2014
Acyrthosiphon pisum virus, *Rosy apple aphid virus*	NC_003780.1	6968..7255	-	Leaky scanning	-	Valles 2014
Allexivirus (*Alphaflexiviridae*)	NC_001800.1	5732..5989	TGB3	Non-AUG initiation (CUG?)	verified[2]	Firth 2012a, Kanyuka 1992
Alphavirus (*Togaviridae*)	NC_001449.1	join(9833..9970, 9970..10047)	TF	-1 PRF	verified	Firth 2008a, Snyder 2013
Ampelovirus (*Plum bark necrosis stem pitting-associated virus*)	NC_009992.1	531..755	-	Leaky scanning	-	-
Aparavirus (IAPV, ABPV, KBV, *Formica exsecta virus 1*)	NC_009025.1	6619..6903	X	Dicistrovirus IGR-IRES	verified	Ren 2012
Arteriviridae (PRRSV, LDV, SHFV, but not EAV)	NC_001961.1	join(1339..3888, 3887..4396)	nsp2TF	-2 PRF	verified	Fang 2012
Arteriviridae	NC_001961.1	13778..13933	5a	Leaky scanning	verified	Firth 2011, Johnson 2011
Mamastrovirus - genogroup I[3] (HAstV and related feline, porcine, canine etc isolates)	NC_001943.1	4419..4757	-	Leaky scanning	-	Firth 2010a
Mamastrovirus - genogroup IV[3] (MLB1, MLB2, MLB3)	NC_011400.1	3850..4086	-	Leaky scanning	-	-
Mamastrovirus - genogroup III[3] (murine and related porcine, bovine, deer and rat isolates)	NC_018702.1	4299..4595	-	Leaky scanning	-	-
Betanodavirus (*Nodaviridae*)	NC_003449.1	join(28..1047, 1047..1277)	CP-FSD	-1 PRF	-	-
Cardiovirus (EMCV)	NC_001479.1	join(3966..3998, 3998..4351)	2B*	-1 PRF	verified	Loughran 2011
Cardiovirus (TMEV, RTV, SAFV etc)	NC_001366.1	join(4233..4250, 4250..4276)	2B*	-1 PRF	-	Loughran 2011
Closteroviridae (*Blueberry virus A*)	NC_018519.1	16185..16313	-	Leaky scanning	-	-
Closteroviridae (*Blueberry virus A*)	NC_018519.1	16297..16491	-	Non-AUG initiation (CUG)	-	-
Deltacoronavirus	NC_011549.1	23951..24220	-	Leaky scanning?	-	Firth 2010a
Alphacoronavirus (bat CoVs 1A, 1B, HKU8)	NC_010437.1	25316..25591	-	Leaky scanning	-	Firth 2010a
Cosavirus (*Picornaviridae*)	NC_012800.1	1202..1393	-	IRES-facilitated leaky scanning?	-	-
Flavivirus (CHAOV-LAMV-DGV clade)	NC_017086.1	join(4213..4467, 4467..4790)	NS2B*	-1 PRF	-	Firth 2010b

Taxon	Example sequence	Nucleotide coordinates	Product	Translation mechanism[1]	Status	References
Flavivirus ('Insect-specific' flaviviruses - CxFV, CFAV, KRV, NAKV, PCV, QBV etc)	NC_008604.2	join(2219..3412, 3412..4293)	NS1′?	-1 PRF	partly verified	Firth 2010b
Flavivirus (WESSV, SEPV)	NC_012735.1	join(3488..3733, 3733..3756)	NS2A*	-1 PRF?	-	-
Flavivirus (JEV-WNV-MVEV-USUV-ALFV clade)	NC_001437.1	join(2478..3560, 3560..3691)	NS1′	-1 PRF	verified	Melian 2010, Firth 2009a
Hepevirus (rat HEV, ferret HEV)	GU345042.1	27..578	-	Leaky scanning	-	Johne 2010
Iflavirus (*Sacbrood virus*, *Halyomorpha halys virus*, *Lygus lineolaris virus 1*)	NC_002066.1	204..530	-	IRES-facilitated leaky scanning?	-	-
Ilarvirus - subgroup 2 and some subgroup 1 and 4 (*Bromoviridae*)	NC_003845.1	1204..2055	CP-RTD	Stop codon readthrough	-	cf. Tzanetakis 2005
Luteoviridae (*Polerovirus*, *Luteovirus*, but not *Enamovirus*)	NC_004750.1	2734..2877	P3a	Non-AUG initiation	-	-
Megrivirus (*Picornaviridae*)	NC_023857.1	8877..9221	-	Reinitiation?	-	Boros 2014
Nebovirus (*Caliciviridae*)	NC_007916.1	5093..5329	-	Leaky scanning	-	Simmonds 2008
Okavirus (*Roniviridae*)	NC_010306.1	20680..20940	-	Leaky scanning	-	Firth 2009b
Picornaviridae (*Pigeon picornavirus B*)	NC_015626.1	405..719	-	IRES-facilitated leaky scanning?	-	cf. Kofstad 2011
Potyviridae (all genera)	NC_002509.2	join(2591..3082, 3085..3261)	P3N-PIPO	Undefined slippage	verified	Chung 2008
Potyvirus (SPFMV, SPVC, SPVG, SPV2)	NC_001841.1	join(118..1389, 1389..2081)	P1N-PISPO	Undefined slippage	-	Clark 2012
Sapovirus - genogroup I (*Caliciviridae*)	NC_006269.1	5180..5665	-	Leaky scanning	-	Liu 1995, Simmonds 2008
Secoviridae (*Black raspberry necrosis virus*)	NC_008182.1	412..663	-	Leaky scanning	-	-
Sobemovirus	NC_001575.2	566..820	Px	Non-AUG initiation	verified	Ling 2013
Tobamovirus (*Passion fruit mosaic virus*, *Maracuja mosaic virus*)	NC_015552.1	4896..5489	-	Leaky scanning	-	Stobbe 2012
Tobamovirus (*Cucumber green mottle mosaic virus*, *Cucumber mottle virus*)	NC_001801.1	5142..5426	-	Leaky scanning	-	-
Torovirus (*Coronaviridae*)	NC_007447.1	520..762	-	Non-AUG initiation (CUG)	-	-
Torovirus (*Coronaviridae*)	NC_007447.1	774..1550	-	Non-AUG initiation (CUG)	-	Firth 2009c
Turdivirus 3 (*Picornaviridae*)	NC_014413.1	603..1205	-	Non-AUG initiation (ACG?)	-	Firth 2012a
Waikavirus (*Secoviridae*)	NC_001632.1	807..1073	-	Leaky scanning?	-	Firth 2008b

Double-stranded RNA viruses

Taxon	Example sequence	Nucleotide coordinates	Product	Translation mechanism[1]	Status	References
Aquareovirus A - VP2 segment (*Atlantic salmon reovirus*, *Scophthalmus maximus reovirus*, *Chum salmon reovirus*)	EF434978.1[4]	116..436	-	Leaky scanning	-	-
Aquareovirus A and *B* - segment 11 (*Threadfin reovirus*, *Scophthalmus maximus reovirus*, *Chum salmon reovirus*, *Green River chinook virus*)	AF524892.1[5]	89..460	-	Leaky scanning	-	-
Aquareovirus A, *B*, *C* and *G* - segment 7	NC_005172.1[6]	195..518	-	Leaky scanning (CUG-initiation in *Aquareovirus A*)	-	-
Coltivirus - VP12 segment	NC_004190.1	315..590	-	Leaky scanning	-	-
Cypovirus - VP1 segment (CPV-1, CPV-5, CPV-14, etc)	NC_003016.1	77..265	-	Leaky scanning	-	Firth 2008c
Cypovirus - RdRp segment (CPV-14)	NC_003006.1	55..351	-	Leaky scanning?	-	-
Cypovirus - RdRp segment (CPV-14, CPV-15)	NC_003006.1	357..605	-	Leaky scanning?	-	-
Cypovirus - segment 6 (CPV-5)	NC_010665.1	10..414	-	Leaky scanning	-	-
Entomobirnavirus - segment A	NC_016518.1	join(103..1896, 1896..2699)	-	-1 PRF?	-	Chung 1996, Marklewitz 2012, Cook 2013
Fijivirus - segment 5 (plant-infecting fijiviruses)	NC_003736.1	join(16..2424, 2426..3073)	-	PAX-like +1 PRF?	-	Firth 2009d, Firth 2012b; but cf. Yang 2014
Magnaporthe oryzae chrysovirus 1 and *3* - segment 2	NC_023042.1	54..1004	-	Leaky scanning	-	-
Orbivirus - VP6 segment	NC_006008.1	182..415	-	Leaky scanning?	verified	Firth 2008d, Ratinier 2011, Belhouchet 2011
Orbivirus - segment 2 (*Stretch Lagoon virus*, *Umatilla virus*)	NC_012755.1	8..271	-	Leaky scanning	-	Cf. Belaganahalli 2011
Rotavirus B and *G* - VP3 segment	NC_021589.1	154..474	-	Leaky scanning	-	-
Seadornavirus - VP7 segment	NC_004204.1	71..229	-	Leaky scanning	-	Firth 2010a

Single-stranded negative-sense RNA viruses

Taxon	Example sequence	Nucleotide coordinates	Product	Translation mechanism[1]	Status	References
Cytorhabdovirus (LNYV, LYMoV)	NC_007642.1	1647..1955	P′	Leaky scanning	-	Dietzgen 2006, Heim 2008, Firth 2010a
Cytorhabdovirus (NCMV)	NC_002251.1	6465..6623	-	Standard	-	Cf. Tanno 2000
Influenza A virus (*Orthomyxoviridae*) - segment 3	NC_002022.1	join(25..597, 599..784)	PA-X	Low level +1 PRF	verified	Jagger 2012, Firth 2012b
Orchid fleck virus, *Citrus necrotic spot virus* (unclassified)	NC_009608.1	2429..2590	-	Reinitiation? (or slippage?)	-	Cf. Kondo 2014
Tospovirus - segment S (WSMoV-CaCV-GBNV clade)	NC_008301.1	110..322	-	Leaky scanning	-	-

Footnotes

1. 'Leaky scanning' for the 'Translation mechanism' entry can indicate that either the novel ORF may be translated via leaky scanning, or the novel ORF initiation site is permissive of leaky scanning to the downstream initiation site of another ORF. 'PRF' stands for 'Programmed ribosomal frameshifting'.
2. Verified by homology to TGBs in related viruses.
3. Astrovirus genogroups follow the scheme of Yokoyama et al. (2012).
4. Note that RefSeq NC_007583.1 has framing issues.
5. Note that RefSeq NC_007591.1 has framing issues.
6. Note that RefSeq NC_007587.1 has framing issues. The ORF is different from the CUG-initiated p22 of Racine et al. (2009); segment 7 predicted to be tricistronic.

References

Belaganahalli MN, Maan S, Maan NS, Tesh R, Attoui H, Mertens PP (2011) Umatilla virus genome sequencing and phylogenetic analysis: identification of stretch lagoon orbivirus as a new member of the Umatilla virus species. PLoS One 6:e23605.

Belhouchet M, Mohd Jaafar F, Firth AE, Grimes JM, Mertens PP, Attoui H (2011) Detection of a fourth orbivirus non-structural protein. PLoS One 6:e25697.

Boros Á, Pankovics P, Knowles NJ, Nemes C, Delwart E, Reuter G (2014) Comparative complete genome analysis of chicken and Turkey megriviruses (family picornaviridae): long 3′ untranslated regions with a potential second open reading frame and evidence for possible recombination. J Virol 88:6434-6443.

Chung HK, Kordyban S, Cameron L, Dobos P (1996) Sequence analysis of the bicistronic Drosophila X virus genome segment A and its encoded polypeptides. Virology 225:359-368.

Chung BY, Miller WA, Atkins JF, Firth AE (2008) An overlapping essential gene in the Potyviridae. Proc Natl Acad Sci U S A 105:5897-5902.

Clark CA, Davis JA, Abad A, Cuellar WJ, Fuentes S, Kreuze JF, Gibson RW, Mukasa SB, Tugume AK, Tairo FD, Valkonen JPT (2012) Sweetpotato Viruses: 15 Years of Progress on Understanding and Managing Complex Diseases. Plant Disease 96:168-185.

Cook S, Chung BY, Bass D, Moureau G, Tang S, McAlister E, Culverwell CL, Glücksman E, Wang H, Brown TD, Gould EA, Harbach RE, de Lamballerie X, Firth AE (2013) Novel virus discovery and genome reconstruction from field RNA samples reveals highly divergent viruses in dipteran hosts. PLoS One 8:e80720.

Dietzgen RG, Callaghan B, Wetzel T, Dale JL (2006) Completion of the genome sequence of Lettuce necrotic yellows virus, type species of the genus Cytorhabdovirus. Virus Res 118:16-22.

Fang Y, Treffers EE, Li Y, Tas A, Sun Z, van der Meer Y, de Ru AH, van Veelen PA, Atkins JF, Snijder EJ, Firth AE (2012) Efficient -2 frameshifting by mammalian ribosomes to synthesize an additional arterivirus protein. Proc Natl Acad Sci U S A 109:E2920-2928.

Firth AE, Chung BY, Fleeton MN, Atkins JF (2008a) Discovery of frameshifting in Alphavirus 6K resolves a 20-year enigma. Virol J 5:108.

Firth AE, Atkins JF (2008b) Bioinformatic analysis suggests that a conserved ORF in the waikaviruses encodes an overlapping gene. Arch Virol 153:1379-1383.

Firth AE, Atkins JF (2008c) Bioinformatic analysis suggests that the Cypovirus 1 major core protein cistron harbours an overlapping gene. Virol J 5:62.

Firth AE (2008d) Bioinformatic analysis suggests that the Orbivirus VP6 cistron encodes an overlapping gene. Virol J 5:48.

Firth AE, Atkins JF (2009a) A conserved predicted pseudoknot in the NS2A-encoding sequence of West Nile and Japanese encephalitis flaviviruses suggests NS1′ may derive from ribosomal frameshifting. Virol J 6:14.

Firth AE, Atkins JF (2009b) Evidence for a novel coding sequence overlapping the 5′-terminal approximately 90 codons of the gill-associated and yellow head okavirus envelope glycoprotein gene. Virol J 6:222.

Firth AE, Atkins JF (2009c) A case for a CUG-initiated coding sequence overlapping torovirus ORF1a and encoding a novel 30 kDa product. Virol J 6:136.

Firth AE, Atkins JF (2009d) Analysis of the coding potential of the partially overlapping 3′ ORF in segment 5 of the plant fijiviruses. Virol J 6:32.

Firth AE, Atkins JF (2010a) Candidates in Astroviruses, Seadornaviruses, Cytorhabdoviruses and Coronaviruses for +1 frame overlapping genes accessed by leaky scanning. Virol J 7:17.

Firth AE, Blitvich BJ, Wills NM, Miller CL, Atkins JF (2010b) Evidence for ribosomal frameshifting and a novel overlapping gene in the genomes of insect-specific flaviviruses. Virology 399:153-166.

Firth AE, Zevenhoven-Dobbe JC, Wills NM, Go YY, Balasuriya UB, Atkins JF, Snijder EJ, Posthuma CC (2011a) Discovery of a small arterivirus gene that overlaps the GP5 coding sequence and is important for virus production. J Gen Virol 92:1097-1106.

Firth AE, Brierley I (2012a) Non-canonical translation in RNA viruses. J Gen Virol 93:1385-1409.

Firth AE, Jagger BW, Wise HM, Nelson CC, Parsawar K, Wills NM, Napthine S, Taubenberger JK, Digard P, Atkins JF (2012b) Ribosomal frameshifting used in influenza A virus expression occurs within the sequence UCC_UUU_CGU and is in the +1 direction. Open Biol 2:120109.

Heim F, Lot H, Delecolle B, Bassler A, Krczal G, Wetzel T (2008) Complete nucleotide sequence of a putative new cytorhabdovirus infecting lettuce. Arch Virol 153:81-92.

Jagger BW, Wise HM, Kash JC, Walters KA, Wills NM, Xiao YL, Dunfee RL, Schwartzman LM, Ozinsky A, Bell GL, Dalton RM, Lo A, Efstathiou S, Atkins JF, Firth AE, Taubenberger JK, Digard P (2012) An overlapping protein-coding region in influenza A virus segment 3 modulates the host response. Science 337:199-204.

Johne R, Heckel G, Plenge-Bönig A, Kindler E, Maresch C, Reetz J, Schielke A, Ulrich RG (2010) Novel hepatitis E virus genotype in Norway rats, Germany. Emerg Infect Dis 16:1452-1455.

Johnson CR, Griggs TF, Gnanandarajah J, Murtaugh MP (2011) Novel structural protein in porcine reproductive and respiratory syndrome virus encoded by an alternative ORF5 present in all arteriviruses. J Gen Virol 92:1107-1116.

Kanyuka KV, Vishnichenko VK, Levay KE, Kondrikov DYu, Ryabov EV, Zavriev SK (1992) Nucleotide sequence of shallot virus X RNA reveals a 5′-proximal cistron closely related to those of potexviruses and a unique arrangement of the 3′-proximal cistrons. J Gen Virol 73:2553-2560.

Kofstad T, Jonassen CM (2011) Screening of feral and wood pigeons for viruses harbouring a conserved mobile viral element: characterization of novel Astroviruses and Picornaviruses. PLoS One 6:e25964.

Kondo H, Maruyama K, Chiba S, Andika IB, Suzuki N (2014) Transcriptional mapping of the messenger and leader RNAs of orchid fleck virus, a bisegmented negative-strand RNA virus. Virology 452-453:166-174.

Ling R, Pate AE, Carr JP, Firth AE (2013) An essential fifth coding ORF in the sobemoviruses. Virology 446:397-408.

Liu BL, Clarke IN, Caul EO, Lambden PR (1995) Human enteric caliciviruses have a unique genome structure and are distinct from the Norwalk-like viruses. Arch Virol 140:1345-1356.

Loughran G, Firth AE, Atkins JF (2011) Ribosomal frameshifting into an overlapping gene in the 2B-encoding region of the cardiovirus genome. Proc Natl Acad Sci U S A 108:E1111-1119.

Marklewitz M, Gloza-Rausch F, Kurth A, Kümmerer BM, Drosten C, Junglen S (2012) First isolation of an Entomobirnavirus from free-living insects. J Gen Virol 93:2431-2435.

Melian EB, Hinzman E, Nagasaki T, Firth AE, Wills NM, Nouwens AS, Blitvich BJ, Leung J, Funk A, Atkins JF, Hall R, Khromykh AA (2010) NS1′ of flaviviruses in the Japanese encephalitis virus serogroup is a product of ribosomal frameshifting and plays a role in viral neuroinvasiveness. J Virol 84:1641-1647.

Racine T, Hurst T, Barry C, Shou J, Kibenge F, Duncan R (2009) Aquareovirus effects syncytiogenesis by using a novel member of the FAST protein family translated from a noncanonical translation start site. J Virol 83:5951-5955.

Ratinier M, Caporale M, Golder M, Franzoni G, Allan K, Nunes SF, Armezzani A, Bayoumy A, Rixon F, Shaw A, Palmarini M (2011) Identification and characterization of a novel non-structural protein of bluetongue virus. PLoS Pathog 7:e1002477.

Ren Q, Wang QS, Firth AE, Chan MM, Gouw JW, Guarna MM, Foster LJ, Atkins JF, Jan E (2012) Alternative reading frame selection mediated by a tRNA-like domain of an internal ribosome entry site. Proc Natl Acad Sci U S A 109:E630-639.

Simmonds P, Karakasiliotis I, Bailey D, Chaudhry Y, Evans DJ, Goodfellow IG (2008) Bioinformatic and functional analysis of RNA secondary structure elements among different genera of human and animal caliciviruses. Nucleic Acids Res 36:2530-2546.

Snyder JE, Kulcsar KA, Schultz KL, Riley CP, Neary JT, Marr S, Jose J, Griffin DE, Kuhn RJ (2013) Functional characterization of the alphavirus TF protein. J Virol 87:8511-8523.

Stobbe AH, Melcher U, Palmer MW, Roossinck MJ, Shen G (2012) Co-divergence and host-switching in the evolution of tobamoviruses. J Gen Virol 93:408-418.

Tanno F, Nakatsu A, Toriyama S, Kojima M (2000) Complete nucleotide sequence of Northern cereal mosaic virus and its genome organization. Arch Virol 145:1373-1384.

Tzanetakis IE, Martin RR (2005) New features in the genus Ilarvirus revealed by the nucleotide sequence of Fragaria chiloensis latent virus. Virus Res 112:32-37.

Valles SM, Bell S, Firth AE (2014) Solenopsis invicta virus 3: mapping of structural proteins, ribosomal frameshifting, and similarities to Acyrthosiphon pisum virus and Kelp fly virus. PLoS One 9:e93497.

Yang J, Zhang HM, Ying L, Li J, Lv MF, Xie L, Li PP, Liu XY, Liang-Ying D, Chen JP (2014) Rice black-streaked dwarf virus genome segment S5 is a bicistronic mRNA in infected plants. Arch Virol 159:307-314.

Yokoyama CC, Loh J, Zhao G, Stappenbeck TS, Wang D, Huang HV, Virgin HW, Thackray LB (2012) Adaptive immunity restricts replication of novel murine astroviruses. J Virol 86:12262-12270.

Dataset S1. Regions of reduced synonymous site variability in RNA viruses

For generic identification of regions of reduced synonymous site variability, we identified codon positions in alignments where the synplot2 p-value for a 25-codon window centred on that codon position was $\leq 10^{-6}$ and the ratio of the observed number to the expected number (obs/exp) of synonymous substitutions in the 25-codon window was ≤ 0.65. Adjacent codon positions satisfying these conditions were merged into regions, and adjacent regions were merged if the gap between them was ≤ 24 codons. Regions are indicated by their nucleotide coordinates in the given RefSeq, e.g. "NC_014320.1: 3975..4046" represents nucleotides 3975 to 4046 of GenBank accession NC_014320.1. Note that, in general, a larger number of statistically significantly conserved regions are found in RefSeqs for which larger more diverse alignments could be generated; the additional limit obs/exp ≤ 0.65 ensures that, even for the largest alignments, only regions subject to strong purifying selection are reported. Note that additional statistically significantly conserved regions are identifiable with different window sizes (e.g. smaller window sizes for compact RNA structures, larger window sizes for extended overlapping genes), and/or with more divergent sequence alignments (the alignments used here were built from full-length sequences with $\geq$75% amino acid identity to the RefSeq). Note that $p \leq 10^{-6}$ is a very conservative threshold, designed to have an expected probability of $\sim$5% of obtaining a single false positive over the analysis of *all* alignments. Note, however, that recombinant sequences can give rise to conserved regions that do not represent overlapping functional elements. Alignments were not systematically screened for recombinants, though three obviously problematic alignments were removed (viz. those for NC_020439.1, NC_016416.1, NC_016081.1; see website).

Single-stranded positive-sense RNA viruses

Astroviridae -
Astrovirus MLB1 HK05 -
    NC_014320.1: 3975..4046
Astrovirus MLB1 -
    NC_011400.1: 3975..4046
Astrovirus MLB2 -
    NC_016155.1: 3975..4046
Astrovirus MLB3 -
    NC_019028.1: 3975..4046

Astroviridae - Mamastrovirus
Mamastrovirus 1 -
    NC_001943.1: 2849..2872, 4189..4396, 4520..4612, 6692..6733

Bromoviridae - Cucumovirus
Cucumber mosaic virus -
    RNA1 NC_002034.1: 104..460, 536..649
    RNA2 NC_002035.1: 357..377, 2442..2591
    RNA3 NC_001440.1: 120..215, 309..320, 876..956, 1569..1619
Gayfeather mild mottle virus -
    RNA1 NC_012134.1: 107..655
Peanut stunt virus -
    RNA1 NC_002038.1: 242..325, 413..439

Bromoviridae - Ilarvirus
Asparagus virus 2 -
    RNA2 NC_011809.1: 2386..2388
Prunus necrotic ringspot virus -
    RNA3 NC_004364.1: 1157..1159

Caliciviridae -

Calicivirus isolate TCG -
NC_006875.1: 5247..5300
Canine calicivirus -
NC_004542.1: 3357..3407
Walrus calicivirus -
NC_004541.1: 10..60, 202..204, 331..384, 2557..2559, 5224..5232, 5620..5696, 8054..8107

Caliciviridae - Lagovirus
European brown hare syndrome virus -
NC_002615.1: 12..110, 5256..5294
Rabbit calicivirus Australia 1 MIC-07 -
NC_011704.1: 109..111, 5263..5316
Rabbit hemorrhagic disease virus -
NC_001543.1: 109..111, 5278..5331

Caliciviridae - Nebovirus
Calicivirus strain NB -
NC_004064.1: 5247..5300
Newbury agent 1 -
NC_007916.1: 5248..5301

Caliciviridae - Norovirus
Murine norovirus 1 -
NC_008311.1: 6..155, 237..302, 4866..5682, 6628..6746, 6846..6857, 7113..7286
Norwalk virus -
NC_001959.2: 5..7, 5312..5384

Caliciviridae - Sapovirus
Sapovirus C12 -
NC_006554.1: 49..63, 5110..5154
Sapovirus Hu-Dresden-pJG-Sap01-DE -
NC_006269.1: 5143..5166, 5251..5448, 5542..5637
Sapovirus Mc10 -
NC_010624.1: 50..64, 5108..5161

Caliciviridae - Vesivirus
Feline calicivirus -
NC_001481.2: 20..190, 2393..2560, 5213..5358, 7216..7325, 7521..7628
Rabbit vesivirus -
NC_008580.1: 32..67, 218..220, 353..397, 2555..2575, 5240..5245, 5630..5721, 8061..8114
Steller sea lion vesivirus -
NC_011050.1: 32..67, 218..220, 356..394, 2555..2575, 5243..5248, 5633..5724, 8073..8126
Vesicular exanthema of swine virus -
NC_002551.1: 26..79, 365..412, 2555..2578, 5243..5248, 5633..5715, 8049..8105

Closteroviridae - Ampelovirus
Plum bark necrosis stem pitting-associated virus -
NC_009992.1: 641..703

Closteroviridae - Closterovirus
Citrus tristeza virus -
NC_001661.1: 9420..9461, 13777..13812, 15251..15292, 17071..17082
Grapevine leafroll-associated virus 2 -
NC_007448.1: 181..198, 15547..15630
Grapevine rootstock stem lesion associated virus -
NC_004724.1: 182..199, 15572..15655

Flaviviridae - Flavivirus Dengue virus group
Dengue virus 1 -
NC_001477.1: 104..289

Dengue virus 2 -
  NC_001474.2: 124..183, 268..285
Dengue virus 3 -
  NC_001475.2: 104..289
Dengue virus 4 -
  NC_002640.1: 114..176

Flaviviridae - Flavivirus Japanese encephalitis virus group
Japanese encephalitis virus -
  NC_001437.1: 96..296, 378..392, 3552..3662
Murray Valley encephalitis virus -
  NC_000943.1: 96..296, 378..395, 3549..3659
St. Louis encephalitis virus -
  NC_007580.2: 102..173
Usutu virus -
  NC_006551.1: 97..300, 3550..3657
West Nile virus - lineage 1 -
  NC_009942.1: 97..297, 379..381, 3544..3651
West Nile virus - lineage 2 -
  NC_001563.2: 97..297, 3532..3642

Flaviviridae - Flavivirus Ntaya virus group
Bagaza virus -
  NC_012534.1: 101..196
Ntaya virus -
  NC_018705.3: 101..196
Tembusu virus -
  NC_015843.2: 101..196

Flaviviridae - Flavivirus Spondweni virus group
Zika virus -
  NC_012532.1: 158..166

Flaviviridae - Flavivirus tick-borne encephalitis virus group
Alkhumra hemorrhagic fever virus -
  NC_004355.1: 115..228
Langat virus -
  NC_003690.1: 131..244, 5237..5239
Louping ill virus -
  NC_001809.1: 130..243
Omsk hemorrhagic fever virus -
  NC_005062.1: 133..246
Powassan virus -
  NC_003687.1: 112..219
Tick-borne encephalitis virus -
  NC_001672.1: 133..246

Flaviviridae - Flavivirus Yellow fever virus group
Sepik virus -
  NC_008719.1: 3750..3770
Wesselsbron virus -
  NC_012735.1: 3752..3772
Yellow fever virus -
  NC_002031.1: 149..247

Flaviviridae - Hepacivirus
Hepatitis C virus genotype 2 -
  NC_009823.1: 341..751, 8000..8068, 8639..8905, 9074..9442
Hepatitis C virus genotype 3 -
  NC_009824.1: 340..843, 7822..7827, 7951..8028, 8647..8676, 8989..9189, 9268..9399

Hepatitis C virus genotype 4 -
NC_009825.1: 280..780, 7852..7929, 8113..8118, 8437..8577, 8902..9306
Hepatitis C virus genotype 5 -
NC_009826.1: 280..777, 7870..7941, 8455..8490, 8569..8604, 8761..8766, 8920..9093, 9190..9324
Hepatitis C virus genotype 6 -
NC_009827.1: 343..846, 7951..8025, 8536..8625, 9010..9165, 9268..9402
Hepatitis C virus -
NC_004102.1: 342..839, 7926..7994, 8499..8645, 8973..9143, 9237..9371

Flaviviridae - Pegivirus
GB virus C -
NC_001710.1: 459..620, 1752..1910, 4533..4559, 4728..4796, 4899..4955, 5208..5498, 6762..6770, 6900..7118, 7260..7361, 7629..7700, 7782..7871, 8682..8762, 8973..9080

Flaviviridae - Pestivirus
Border disease virus -
NC_003679.1: 373..420
Bovine viral diarrhea virus 1 -
NC_001461.1: 386..442
Bovine viral diarrhea virus 2 -
NC_002032.1: 365..373
Classical swine fever virus -
NC_002657.1: 374..421
Pestivirus strain Aydin-04-TR -
NC_018713.1: 378..425

Hepeviridae -
Avian hepatitis E virus -
NC_023425.1: 55..252, 4716..4922

Hepeviridae - Hepevirus
Ferret hepatitis E virus -
JN998606.1: 94..255, 4824..4886
Hepatitis E virus -
NC_001434.1: 4..132, 5077..5461, 6278..6364, 7058..7105
Hepatitis E virus rat-R63-DEU-2009 -
GU345042.1: 11..439, 4941..5026, 5207..5209, 6668..6670, 6809..6826

Leviviridae - Allolevivirus
Enterobacteria phage FI sensu lato -
NC_004301.1: 4086..4094

Leviviridae - Levivirus
Enterobacteria phage MS2 -
NC_001417.2: 1803..1823

Luteoviridae - Luteovirus
Barley yellow dwarf virus-GAV -
NC_004666.1: 1130..1170, 2611..2709, 2828..3254
Barley yellow dwarf virus-MAV -
NC_003680.1: 1093..1148, 1299..1304, 2574..2630, 2858..3202
Barley yellow dwarf virus-PAS -
NC_002160.2: 1144..1169, 2604..3325
Barley yellow dwarf virus-PAV -
NC_004750.1: 1138..1205, 1857..1880, 2601..3280

Luteoviridae - Polerovirus
Cucurbit aphid-borne yellows virus -
NC_003688.1: 241..267, 3613..3657, 3733..3753, 3874..3888

Nidovirales - Arteriviridae - Arterivirus
Equine arteritis virus -
NC_002532.2: 10454..10471, 12357..12359
Porcine reproductive and respiratory syndrome virus -
NC_001961.1: 190..273, 4066..4344, 7693..7742, 12076..12261, 12769..12840, 13287..13370, 13806..13883, 14331..14366, 14603..14930

Nidovirales - Coronaviridae - Coronavirinae -
Bat coronavirus BM48-31-BGR-2008 -
NC_014470.1: 11339..11353, 13318..13344, 25947..26164, 26857..26871, 27911..27943

Nidovirales - Coronaviridae - Coronavirinae - Alphacoronavirus
Feline infectious peritonitis virus (possible overprediction due to recombinants) -
NC_002306.3: 338..352, 452..523, 12404..12456, 12580..12639, 13579..13590, 13963..13968, 15577..15900, 15985..16044, 17752..17754, 18205..18207, 18736..18786, 18868..19026, 19114..19647, 19831..19851, 20065..20091, 20182..20199, 20344..20352, 25461..25472, 25566..25574, 25653..25811, 25887..25937, 26936..27073, 27743..27790, 28133..28184, 28392..28457

Nidovirales - Coronaviridae - Coronavirinae - Betacoronavirus
Rousettus bat coronavirus HKU9 -
NC_009021.1: 12949..13004
SARS coronavirus -
NC_004718.3: 11440..11454, 13405..13445, 26036..26263, 26938..26952, 28372..28404

Nidovirales - Coronaviridae - Coronavirinae - Gammacoronavirus
Infectious bronchitis virus (possible overprediction due to recombinants) -
NC_001451.1: 529..591, 796..798, 2329..2334, 5131..5172, 8881..8886, 9052..9054, 9592..9651, 10027..10089, 10195..10212, 10579..10779, 10858..10860, 10981..11055, 11611..11664, 12319..12422, 12534..12548, 12828..12857, 14004..14006, 16197..16238, 16494..16580, 20205..20234, 23800..23838, 24679..24690, 25075..25092, 25688..25819, 26632..26646
Turkey coronavirus (possible overprediction due to recombinants) -
NC_010800.1: 529..591, 5137..5181, 8881..8886, 9592..9645, 10027..10089, 10195..10212, 10579..10776, 10984..11046, 11611..11661, 12319..12416, 12534..12548, 12831..12854, 16197..16235, 16503..16574, 20199..20222, 24805..24816, 25201..25218, 25303..25305, 25878..25991, 26813..26827

Nidovirales - Mesoniviridae -
Hana virus -
NC_020899.1: 18682..18699

Nidovirales - Mesoniviridae - Alphamesonivirus
Cavally virus -
NC_015668.1: 15503..15505, 16115..16117, 18718..18744
Nam Dinh virus -
NC_015874.1: 15513..15515, 16119..16121, 18732..18749

Nodaviridae - Betanodavirus
Barfin flounder nervous necrosis virus -
RNA1 NC_013458.1: 2752..2907
RNA2 NC_013459.1: 306..404, 1064..1075
Barfin flounder virus BF93Hok -
RNA1 NC_011063.1: 2752..2907
RNA2 NC_011064.1: 306..404, 1064..1075
Epinephelus tauvina nervous necrosis virus -
RNA1 NC_004137.1: 2749..2907
RNA2 NC_004136.1: 306..404, 1064..1075
Redspotted grouper nervous necrosis virus -
RNA1 NC_008040.1: 2749..2907
RNA2 NC_008041.1: 306..404, 1064..1075
Striped Jack nervous necrosis virus -
RNA1 NC_003448.1: 2752..2910
RNA2 NC_003449.1: 310..405, 1071..1082
Tiger puffer nervous necrosis virus -

RNA1 NC_013460.1: 2760..2915
RNA2 NC_013461.1: 313..405, 1071..1088

Picornavirales - Dicistroviridae - Aparavirus
Formica exsecta virus 1 -
NC_023021.1: 6757..6795
Israeli acute paralysis virus -
NC_009025.1: 6747..6779
Kashmir bee virus -
NC_004807.1: 6747..6815

Picornavirales - Iflaviridae - Iflavirus
Sacbrood virus -
NC_002066.1: 347..397

Picornavirales - Picornaviridae - Aphthovirus
Foot-and-mouth disease virus - type O -
NC_004004.1: 4134..4430, 5883..5978, 6156..6239, 7557..7616, 7725..8027

Picornavirales - Picornaviridae - Avihepatovirus
Duck hepatitis A virus 1 -
NC_008250.2: 4005..4037

Picornavirales - Picornaviridae - Cardiovirus
Encephalomyocarditis virus -
NC_001479.1: 4005..4178, 6903..6905, 7386..7430, 7506..7511
Human TMEV-like cardiovirus -
NC_010810.1: 956..991, 4097..4171, 6170..6175, 7475..7837
Saffold virus -
NC_009448.2: 1041..1070, 4194..4265, 6264..6269, 7566..7814, 7890..7931
Theilovirus -
NC_001366.1: 1107..1307, 4230..4292, 7638..7655

Picornavirales - Picornaviridae - Cosavirus
Cosavirus A -
NC_012800.1: 1228..1332, 6055..6084, 6928..7035, 7237..7353

Picornavirales - Picornaviridae - Enterovirus
Enterovirus A -
NC_001612.1: 4411..4458
Enterovirus B -
NC_001472.1: 4348..4389
Enterovirus C -
NC_002058.3: 4457..4459, 5948..5953, 6932..7054, 7319..7372
Enterovirus E -
NC_001859.1: 7158..7190
Enterovirus F -
NC_021220.1: 4399..4437, 6454..6498, 7165..7305
Possum enterovirus W1 -
NC_008714.1: 4392..4430, 6447..6491, 7158..7298

Picornavirales - Picornaviridae - Hepatovirus
Hepatitis A virus -
NC_001489.1: 750..803, 5958..6035, 7341..7418

Picornavirales - Picornaviridae - Kobuvirus
Caprine kobuvirus -
NC_023422.1: 684..686
Porcine kobuvirus SH-W-CHN-2010-China -
NC_016769.1: 577..579, 5389..5391

Porcine kobuvirus swine-S-1-HUN-2007-Hungary -
NC_011829.1: 577..579, 5389..5391

Picornavirales - Picornaviridae - Parechovirus
Human parechovirus -
NC_001897.1: 1357..1428, 6862..6906, 7012..7242
Ljungan virus -
NC_003976.2: 7405..7407

Picornavirales - Picornaviridae - Sapelovirus
Simian sapelovirus 1 -
NC_004451.1: 7690..7695

Picornavirales - Picornaviridae - Teschovirus
Porcine teschovirus -
NC_003985.1: 336..536, 6150..6230, 6450..6458, 6711..6860

Picornavirales - Secoviridae -
Black raspberry necrosis virus -
RNA1 NC_008182.1: 504..560

Picornavirales - Secoviridae - Comovirinae - Fabavirus
Broad bean wilt virus 2 -
RNA2 NC_003004.1: 389..403

Picornavirales - Secoviridae - Comovirinae - Nepovirus Subgroup A
Arabis mosaic virus -
RNA1 NC_006057.1: 435..473, 588..617, 780..833
RNA2 NC_006056.1: 398..400, 647..757
Grapevine deformation virus -
RNA1 NC_017939.1: 1296..1298
Grapevine fanleaf virus -
RNA1 NC_003615.1: 474..476, 612..626, 780..866
RNA2 NC_003623.1: 506..712, 791..826
Potato black ringspot virus -
RNA1 NC_022798.1: 1083..1139
Tobacco ringspot virus -
RNA1 NC_005097.1: 998..1066

Potyviridae - Poacevirus
Sugarcane streak mosaic virus -
NC_014037.1: 3233..3289

Potyviridae - Potyvirus
Bean common mosaic virus -
NC_003397.1: 3257..3328, 3410..3421, 9212..9214, 9548..9688
Bean yellow mosaic virus -
NC_003492.1: 2891..3061, 9260..9361
Bidens mosaic virus -
NC_023014.1: 9244..9255
Calla lily latent virus -
NC_021196.1: 1007..1033, 3002..3181, 9344..9382
Chilli veinal mottle virus -
NC_005778.1: 3050..3058, 9155..9178
Hardenbergia mosaic virus -
NC_015394.2: 3083..3085
Leek yellow stripe virus -
NC_004011.1: 691..846, 3169..3207, 9349..9372, 9496..9498
Maize dwarf mosaic virus -
NC_003377.1: 2777..2899

Narcissus late season yellows virus -
NC_023628.1: 9066..9080
Narcissus yellow stripe virus -
NC_011541.1: 9107..9136
Onion yellow dwarf virus -
NC_005029.1: 4047..4055
Papaya ringspot virus -
NC_001785.1: 3644..3709, 10037..10099
Peru tomato mosaic virus -
NC_004573.1: 3082..3099, 9307..9321
Plum pox virus -
NC_001445.1: 219..242, 2967..3077, 3174..3182, 9147..9524
Potato virus V -
NC_004010.1: 3073..3090, 9301..9315
Potato virus Y -
NC_001616.1: 2948..3082, 8894..8947, 9035..9106, 9287..9346
Sorghum mosaic virus -
NC_004035.1: 2790..2939
Soybean mosaic virus -
NC_002634.1: 912..935, 2904..3083, 9192..9242
Sugarcane mosaic virus -
NC_003398.1: 2718..2924, 8880..8882, 9180..9338
Sunflower chlorotic mottle virus -
NC_014038.1: 9649..9675
Sweet potato feathery mottle virus -
NC_001841.1: 10474..10569
Sweet potato virus C -
NC_014742.1: 10472..10570
Turnip mosaic virus -
NC_002509.2: 131..442, 3089..3256, 9158..9625
Watermelon mosaic virus -
NC_006262.1: 3334..3462, 9676..9699
Wild potato mosaic virus -
NC_004426.1: 3052..3069, 9277..9291
Wisteria vein mosaic virus -
NC_007216.1: 942..1004, 2964..3143, 9303..9353
Yam bean mosaic virus -
NC_016441.1: 2974..2988, 3064..3099, 9214..9303
Yam mild mosaic virus -
NC_019412.1: 3036..3110
Zucchini yellow mosaic virus -
NC_003224.1: 2974..3045, 9289..9291

Potyviridae - Tritimovirus
Oat necrotic mottle virus -
NC_005136.1: 176..190, 2771..3094
Wheat streak mosaic virus -
NC_001886.1: 182..187, 2768..3097

Sobemovirus
Rice yellow mottle virus -
NC_001575.2: 642..707, 1989..2393, 3539..3589

Togaviridae - Alphavirus EEEV complex
Eastern equine encephalitis virus -
NC_003899.1: 101..235, 1040..1126, 9947..10018
Madariaga virus -
NC_023812.1: 82..231, 1036..1122, 9899..9973

Togaviridae - Alphavirus SFV complex

Chikungunya virus -
NC_004162.2: 9985..10023
O-nyong-nyong virus -
NC_001512.1: 10085..10123

Togaviridae - Alphavirus VEEV complex
Venezuelan equine encephalitis virus -
NC_001449.1: 60..242, 846..1013, 9944..10027

Togaviridae - Alphavirus WEEV complex
Fort Morgan virus -
NC_013528.1: 140..160, 9834..9887
Highlands J virus -
NC_012561.1: 154..159, 9798..9851
Sindbis virus -
NC_001547.1: 150..278, 7647..7652, 10041..10076
Western equine encephalitis virus -
NC_003908.1: 118..138, 9831..9884
Whataroa virus -
NC_016961.1: 151..279, 7474..7531, 9923..9958

Tombusviridae - Carmovirus
Melon necrotic spot virus -
NC_001504.1: 2347..2349

Tombusviridae - Tombusvirus
Artichoke mottled crinkle virus -
NC_001339.1: 1080..1361, 1590..1616, 3898..4311
Cucumber necrosis virus -
NC_001469.1: 1052..1219, 1301..1333, 1586..1588, 3821..4207
Cymbidium ringspot virus -
NC_003532.1: 1040..1234, 1310..1345, 1571..1597, 3843..3881, 3978..4214
Eggplant mottled crinkle virus -
NC_023339.1: 1068..1355, 1578..1604, 3875..4288
Grapevine Algerian latent virus -
NC_011535.1: 1064..1240, 1316..1351, 1559..1606, 3800..3919, 4010..4213
Lettuce necrotic stunt virus -
NC_018104.1: 1070..1348, 1577..1606, 3884..3919, 4055..4153, 4238..4246
Lisianthus necrosis virus -
NC_007983.1: 1062..1352, 1572..1610, 3866..4285
Moroccan pepper virus -
NC_020073.1: 1070..1234, 1316..1351, 1577..1603, 3887..3919, 4058..4150, 4238..4246
Pear latent virus -
NC_004723.1: 1067..1240, 1316..1351, 1565..1606, 3871..4278
Tomato bushy stunt virus -
NC_001554.1: 1060..1353, 1573..1611, 3877..4296

Tymovirales - Alphaflexiviridae - Potexvirus
Pepino mosaic virus -
NC_004067.1: 5414..5419
Plantago asiatica mosaic virus -
NC_003849.1: 5075..5095
Potato virus X -
NC_011620.1: 4453..4527, 5171..5203

Tymovirales - Betaflexiviridae -
Cherry green ring mottle virus -
NC_001946.1: 6264..6323
Cherry necrotic rusty mottle virus -
NC_002468.1: 6064..6108, 6285..6620, 7654..7665, 7885..7917, 8206..8235

Cherry rusty mottle associated virus -
NC_020996.1: 6014..6073, 6244..6558, 7162..7182, 7618..7866, 8077..8193

Tymovirales - Betaflexiviridae - Capillovirus
Apple stem grooving virus -
NC_001749.2: 132..158, 4806..5777, 5991..6062, 6312..6329

Tymovirales - Betaflexiviridae - Carlavirus
Blueberry scorch virus -
NC_003499.1: 98..106, 8093..8101, 8388..8393
Kalanchoe latent virus -
NC_013006.1: 7243..7245
Potato virus S -
NC_007289.1: 5994..6032, 7103..7180

Tymovirales - Betaflexiviridae - Foveavirus
Apple green crinkle associated virus -
NC_018714.1: 61..138, 214..234, 6610..6723, 7646..7690, 7797..7835, 7924..7932
Apple stem pitting virus -
NC_003462.2: 60..134, 213..230, 6609..6611, 6711..6752, 7678..7725, 7829..7867, 7956..7964
Apricot latent virus -
NC_014821.1: 61..138, 214..231, 7644..7679, 7916..7921
Grapevine rupestris stem pitting-associated virus -
NC_001948.1: 77..124

Tymovirales - Betaflexiviridae - Trichovirus
Apple chlorotic leaf spot virus -
NC_001409.1: 374..427, 5549..5611, 5726..5806, 6606..6632, 6750..7091
Cherry mottle leaf virus -
NC_002500.1: 5788..5790, 6866..6913

Tymovirales - Betaflexiviridae - Vitivirus
Grapevine virus A -
NC_003604.2: 90..125, 5392..5421, 6368..6593, 6840..7244
Grapevine virus B -
NC_003602.1: 6480..6488

Virgaviridae - Tobamovirus
Bell pepper mottle virus -
NC_009642.1: 4710..4763
Cucumber fruit mottle mosaic virus -
NC_002633.1: 4944..4985
Kyuri green mottle mosaic virus -
NC_003610.1: 4951..4992
Maracuja mosaic virus -
NC_008716.1: 4802..4816
Rehmannia mosaic virus -
NC_009041.1: 4731..4784
Ribgrass mosaic virus -
NC_002792.2: 4736..4795, 5590..5670, 5780..5824
Tobacco mosaic virus -
NC_001367.1: 4728..4790
Tomato mosaic virus -
NC_002692.1: 4731..4832
Tomato mottle mosaic virus -
NC_022230.1: 4735..4788
Turnip vein-clearing virus -
NC_001873.1: 4736..4795, 5590..5670, 5798..5824
Wasabi mottle virus -
NC_003355.1: 4720..4782, 5577..5657, 5782..5811

Youcai mosaic virus -
NC_004422.1: 4725..4784, 5582..5662, 5772..5816
Zucchini green mottle mosaic virus -
NC_003878.1: 4953..4994

Double-stranded RNA viruses

Birnaviridae - Avibirnavirus
Infectious bursal disease virus -
Seg-A NC_004178.1: 169..375, 532..534, 2878..2895, 3046..3060
Seg-B NC_004179.1: 1706..1708, 2525..2599

Reoviridae - Sedoreovirinae - Orbivirus
African horse sickness virus -
Seg-10 NC_006009.1: 161..172
Bluetongue virus -
Seg-1 NC_006023.1: 12..80
Seg-10 NC_006015.1: 20..61, 191..259, 677..709
Seg-3 NC_006014.1: 18..59, 2661..2723
Seg-4 NC_006024.2: 9..41, 1902..1943
Seg-5 NC_006025.1: 35..97
Seg-6 NC_006010.1: 30..35, 1566..1610
Seg-7 NC_006022.1: 18..65, 1026..1067
Seg-8 NC_006007.1: 32..52, 1013..1084
Seg-9 NC_006008.1: 31..33, 211..393
Changuinola virus -
Seg-1 NC_022639.1: 13..21
Seg-10 NC_022638.1: 17..37
Seg-4 NC_022640.1: 9..38, 1929..1931
Seg-6 NC_022641.1: 30..47, 1575..1616
Epizootic hemorrhagic disease virus serotype 1 - strain New Jersey -
Seg-1 NC_013396.1: 15..53
Seg-10 NC_013405.1: 21..35
Seg-3 NC_013398.1: 21..56, 2652..2717

Reoviridae - Sedoreovirinae - Rotavirus
Rotavirus A -
Seg-1 NC_011507.2: 19..81
Seg-10 NC_011504.2: 42..68
Seg-11 NC_011505.2: 70..291
Seg-2 NC_011506.2: 17..67
Seg-3 NC_011508.2: 50..97
Seg-4 NC_011510.2: 10..51
Seg-6 NC_011509.2: 1203..1217
Seg-7 NC_011501.2: 26..79
Seg-8 NC_011502.2: 47..112
Seg-9 NC_011503.2: 49..96
Rotavirus C -
Seg-8 NC_007571.1: 49..84

Reoviridae - Spinareovirinae - Aquareovirus
Aquareovirus A -
Seg-8 NC_007588.1: 112..123

Reoviridae - Spinareovirinae - Cypovirus
Cypovirus 1 -
Seg-4 NC_003019.1: 884..886
Seg-6 NC_003021.1: 1678..1689

Reoviridae - Spinareovirinae - Fijivirus Fijivirus group 2
Rice black streaked dwarf virus -
Seg-10 NC_003733.1: 22..90, 403..423, 619..651
Seg-8 NC_003732.1: 28..66
Southern rice black-streaked dwarf virus -
Seg-10 NC_014713.1: 22..90, 403..420, 628..651
Seg-9 NC_014712.1: 83..100

Reoviridae - Spinareovirinae - Orthoreovirus
Avian orthoreovirus -
Seg-L1 NC_015126.1: 21..65
Seg-L2 NC_015127.1: 32..67
Seg-L3 NC_015128.1: 13..60
Seg-M1 NC_015129.1: 13..75
Seg-M2 NC_015130.1: 2043..2060
Seg-M3 NC_015131.1: 28..72
Seg-S2 NC_015133.1: 16..72
Seg-S4 NC_015135.1: 24..95, 1122..1127
Mammalian orthoreovirus 3 -
Seg-M2 NC_004278.1: 2107..2145
Seg-M2 NC_013228.1: 2074..2127
Seg-S1 NC_004277.1: 271..366
Seg-S1 NC_013231.1: 259..354
Seg-S2 NC_004279.1: 19..69
Seg-S2 NC_013232.1: 19..69
Seg-S4 NC_004276.1: 36..53, 156..218
Seg-S4 NC_013234.1: 124..183

Totiviridae -
Tianjin totivirus -
NC_017084.1: 55..75, 4981..5022

Totiviridae - Totivirus
Saccharomyces cerevisiae virus L-A -
NC_003745.1: 1967..2023

Totiviridae - Trichomonasvirus
Trichomonas vaginalis virus 1 -
NC_003824.1: 288..413, 2539..2556, 2833..2850
Trichomonas vaginalis virus 2 -
NC_003873.1: 2422..2439

Single-stranded negative-sense RNA viruses

Bunyaviridae - Hantavirus
Andes virus -
Seg-M NC_003467.2: 52..105
Seg-S NC_003466.1: 43..300
Dobrava-Belgrade virus -
Seg-L NC_005235.1: 71..82
Seg-M NC_005234.1: 41..94
Seg-S NC_005233.1: 60..65
Hantaan virus -
Seg-L NC_005222.1: 74..82
Seg-M NC_005219.1: 41..94
Seg-S NC_005218.1: 37..66
Hantavirus Z10 -
Seg-L NC_006435.1: 74..82
Seg-M NC_006437.1: 41..94

Seg-S NC_006433.1: 37..66
Puumala virus -
Seg-M NC_005223.1: 41..127
Seg-S NC_005224.1: 43..357
Seoul virus -
Seg-L NC_005238.1: 73..81
Seg-M NC_005237.1: 47..94
Seg-S NC_005236.1: 43..75
Sin Nombre virus -
Seg-M NC_005215.1: 52..102
Seg-S NC_005216.1: 43..303
Tula virus -
Seg-M NC_005228.1: 56..127
Seg-S NC_005227.2: 43..324

Bunyaviridae - Orthobunyavirus
Aino virus -
Seg-S NC_018460.1: 59..268
Akabane virus -
Seg-S NC_009896.1: 67..285
Bunyamwera virus -
Seg-S NC_001927.1: 98..373
Cat Que virus -
Seg-S NC_024075.1: 60..284
La Crosse virus -
Seg-M NC_004109.1: 122..124
Seg-S NC_004110.1: 82..363
Oropouche virus -
Seg-S NC_005777.1: 57..293, 726..740
Sathuperi virus -
Seg-S NC_018462.1: 74..295
Shamonda virus -
Seg-S NC_018464.1: 61..282
Simbu virus -
Seg-S NC_018477.1: 58..276

Bunyaviridae - Phlebovirus
Candiru virus -
Seg-L NC_015374.1: 25..42
Rift Valley fever virus -
Seg-S NC_014395.1: 48..95
Sandfly fever Naples virus -
Seg-L NC_006319.1: 19..69
SFTS virus HB29 -
Seg-S NC_018137.1: 67..69, 709..774

Bunyaviridae - Tospovirus
Capsicum chlorosis virus -
Seg-M NC_008303.1: 75..77
Tomato zonate spot virus -
Seg-M NC_010490.1: 67..99

Mononegavirales - Bornaviridae - Bornavirus
Borna disease virus -
NC_001607.1: 2278..2283

Mononegavirales - Paramyxoviridae - Paramyxovirinae -
Beilong virus -
NC_007803.1: 2574..2684, 8226..8234

Mononegavirales - Paramyxoviridae - Paramyxovirinae - Avulavirus
Goose paramyxovirus SF02 -
NC_005036.1: 2391..2579, 9032..9037
Newcastle disease virus B1 -
NC_002617.1: 2385..2573, 9023..9034

Mononegavirales - Paramyxoviridae - Paramyxovirinae - Henipavirus
Hendra virus -
NC_001906.3: 2727..2768, 3660..3665
Nipah virus -
NC_002728.1: 2745..2786, 3684..3689

Mononegavirales - Paramyxoviridae - Paramyxovirinae - Morbillivirus
Canine distemper virus -
NC_001921.1: 2140..2154, 2530..2580
Measles virus -
NC_001498.1: 2005..2007, 2164..2304, 2515..2580
Rinderpest virus strain Kabete O -
NC_006296.2: 2170..2187, 2278..2301

Mononegavirales - Paramyxoviridae - Paramyxovirinae - Respirovirus
Bovine parainfluenza virus 3 -
NC_002161.1: 2039..2377, 2843..2848
Human parainfluenza virus 3 -
NC_001796.2: 2039..2362, 2861..2866
Sendai virus -
NC_001552.1: 2255..2266

Mononegavirales - Paramyxoviridae - Paramyxovirinae - Rubulavirus
Mumps virus -
NC_002200.1: 2493..2636

Mononegavirales - Rhabdoviridae - Lyssavirus
Aravan virus -
NC_020808.1: 71..85
Australian bat lyssavirus -
NC_003243.1: 4..12, 2413..2439
Duvenhage virus -
NC_020810.1: 71..88
European bat lyssavirus 1 -
NC_009527.1: 71..85
European bat lyssavirus 2 -
NC_009528.1: 74..85
Irkut virus -
NC_020809.1: 71..82
Rabies virus -
NC_001542.1: 74..88

Orthomyxoviridae - Influenzavirus A
Influenza A virus A-Puerto Rico-8-1934 H1N1 -
Seg-1 NC_002023.1: 34..81, 2209..2307
Seg-2 NC_002021.1: 25..135, 2236..2298
Seg-3 NC_002022.1: 28..81, 601..768, 2053..2175
Seg-5 NC_002019.1: 46..93, 1396..1542
Seg-7 NC_002016.1: 26..253, 795..1007
Seg-8 NC_002020.1: 45..113, 528..707

Orthomyxoviridae - Influenzavirus B
Influenza B virus -
RNA4 NC_002207.1: 64..66

RNA5 NC_002208.1: 1663..1722

Orthomyxoviridae - Isavirus
Infectious salmon anemia virus -
Seg-3 NC_006502.1: 82..93
Seg-4 NC_006501.1: 29..61, 464..508, 695..805

Tenuivirus
Rice stripe virus -
RNA3 NC_003776.1: 207..227
RNA4 NC_003753.1: 55..120
