# Supplementary material for: Mapping overlapping functional elements embedded within the protein-coding regions of RNA viruses
Source: Nucleic Acids Res. 2014 Oct 17;42(20):12425–39. doi: 10.1093/nar/gku981 (PMC4227794; doi:10.1093/nar/gku981)
Supplement: SUPPLEMENTARY DATA [file supp_gku981_Supplementary_File_S1.zip › SynPlot2/tree.pdf]

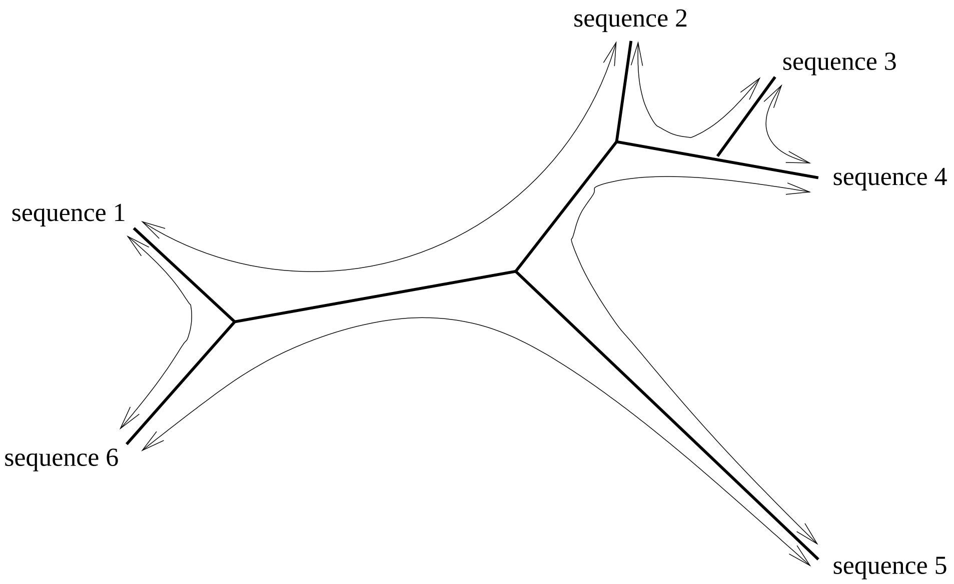

Example phylogenetic tree. The (unrooted) tree is used to select a list of sequence pairs tracing round the outside of the tree. For this tree, the pairs files would be:

```
sequence1 sequence2
sequence2 sequence3
sequence3 sequence4
sequence4 sequence5
sequence5 sequence6
sequence6 sequence1
```

Pairwise conservation scores are calculated for each pair and summed over the tree. Note that this set of pairwise comparisons covers each branch of the tree precisely twice – hence no branch is given more weight than another. In general, the set of pairs selected in this way is not unique, since branches of the tree may be flipped into different places without changing the phylogeny.

It is assumed that the user-input list of sequence pairs is selected in this way (i.e. such that every branch of the assumed phylogenetic tree is covered precisely twice). After calculating the observed and expected number of synonymous nucleotide substitutions in each column of the input alignment (i.e. summed over the given pairwise comparisons), synplot2 divides these numbers by two to obtain, more-or-less, the absolute number of independent synonymous nucleotide substitutions in each column. Obtaining the correct absolute numbers (as opposed to just relative numbers) is important for calculating the p-values.
