# Supplementary figures and images for: Mapping overlapping functional elements embedded within the protein-coding regions of RNA viruses
Source: Nucleic Acids Res. 2014 Oct 17;42(20):12425–39. doi: 10.1093/nar/gku981 (PMC4227794; doi:10.1093/nar/gku981)

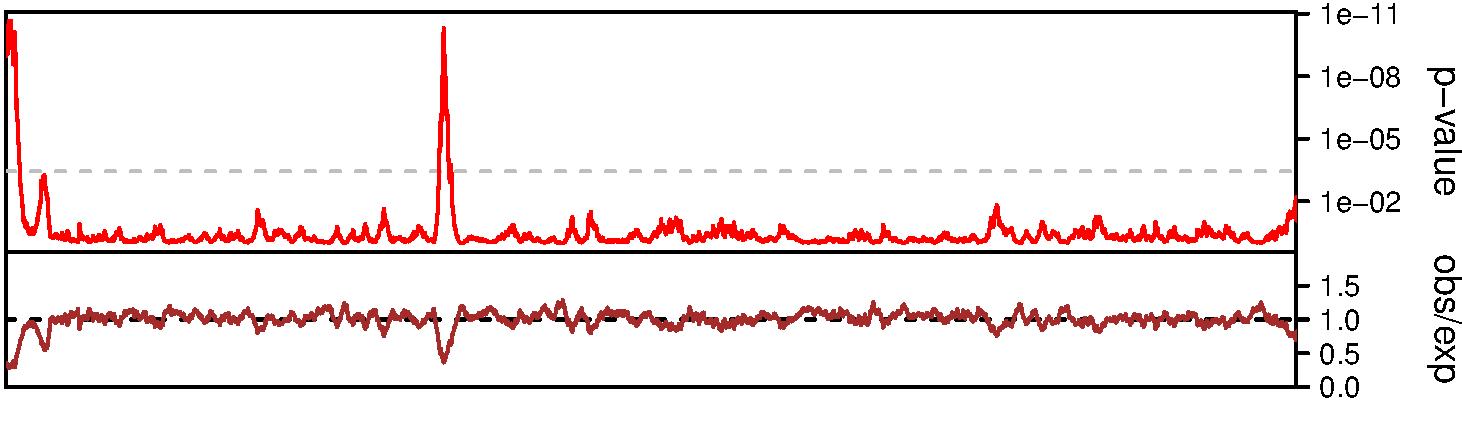

Supplement: SUPPLEMENTARY DATA [file supp_gku981_Supplementary_File_S1.zip › SynPlot2/plot.jpg]
